# Evaluation of dispersion type metal···π arene interaction in arylbismuth compounds – an experimental and theoretical study

**DOI:** 10.3762/bjoc.14.187

**Published:** 2018-08-15

**Authors:** Ana-Maria Preda, Małgorzata Krasowska, Lydia Wrobel, Philipp Kitschke, Phil C Andrews, Jonathan G MacLellan, Lutz Mertens, Marcus Korb, Tobias Rüffer, Heinrich Lang, Alexander A Auer, Michael Mehring

**Affiliations:** 1 Technische Universität Chemnitz, Fakultät für Naturwissenschaften, Institut für Chemie, Professur Koordinationschemie, 09107 Chemnitz, Germany; 2 Max-Planck-Institut für Kohlenforschung, Kaiser-Wilhelm-Platz 1, 45470 Mülheim an der Ruhr, Germany; 3 School of Chemistry, Monash University, Clayton, Melbourne, VIC 3800, Australia; 4 Technische Universität Chemnitz, Fakultät für Naturwissenschaften, Institut für Chemie, Professur Anorganische Chemie, 09107 Chemnitz, Germany

**Keywords:** arylbismuth compounds, DFT-D, dispersion type Bi···π arene interaction, DLPNO-CCSD(T), electronic structure calculations, polymorphism, single crystal X-ray structure

## Abstract

The dispersion type Bi···π arene interaction is one of the important structural features in the assembly process of arylbismuth compounds. Several triarylbismuth compounds and polymorphs are discussed and compared based on the analysis of single crystal X-ray diffraction data and computational studies. First, the crystal structures of polymorphs of Ph_3_Bi (**1**) are described emphasizing on the description of London dispersion type bismuth···π arene interactions and other van der Waals interactions in the solid state and the effect of it on polymorphism. For comparison we have chosen the substituted arylbismuth compounds (C_6_H_4_-CH═CH_2_-4)_3_Bi (**2**), (C_6_H_4_-OMe-4)_3_Bi (**3**), (C_6_H_3_-*t*-Bu_2_-3,5)_3_Bi (**4**) and (C_6_H_3_-*t*-Bu_2_-3,5)_2_BiCl (**5**). The structural analyses revealed that only two of them show London dispersion type bismuth···π arene interactions. One of them is the styryl derivative **2**, for which two polymorphs were isolated. Polymorph **2a** crystallizes in the orthorhombic space group *P*2_1_2_1_2_1_, while polymorph **2b** exhibits the monoclinic space group *P*2_1_/*c*. The general structure of **2a** is similar to the monoclinic *C*2/*c* modification of Ph_3_Bi (**1a**), which leads to the formation of zig-zag Bi–arene_centroid_ ribbons formed as a result of bismuth···π arene interactions and π···π intermolecular contacts. In the crystal structures of the polymorph **2b** as well as for **4** bismuth···π arene interactions are not observed, but both compounds revealed C–H_Ph_···π intermolecular contacts, as likewise observed in all of the three described polymorphs of Ph_3_Bi. For compound **3** intermolecular contacts as a result of coordination of the methoxy group to neighboring bismuth atoms are observed overruling Bi···π arene contacts. Compound **5** shows a combination of donor acceptor Bi···Cl and Bi···π arene interactions, resulting in an intermolecular pincer-type coordination at the bismuth atom. A detailed analysis of three polymorphs of Ph_3_Bi (**1**), which were chosen as model systems, at the DFT-D level of theory supported by DLPNO-CCSD(T) calculations reveals how van der Waals interactions between different structural features balance in order to stabilize molecular arrangements present in the crystal structure. Furthermore, the computational results allow to group this class of compounds into the range of heavy main group element compounds which have been characterized as dispersion energy donors in previous work.

## Introduction

Although known for more than a century, the interest on metal···π arene interaction of main group metals has increased significantly, both experimentally and theoretically in the past decade [1–5]. Especially the development of novel computational tools demonstrated the importance of London dispersion type interactions for structures and functions of molecules [6–8]. With regard to this the high relevance of London dispersion type interactions in molecular organometallic chemistry was recently summarized by Liptrot and Power [9]. It should be noted that in this context and more generally organometallic bismuth compounds are witnessing growing attention since applications in the field of supramolecular chemistry [10–12] and pharmacology are of interest [13–15].

Lately, several studies regarding the metal···π interactions in organometallic compounds of antimony and bismuth [16–19] have been reported including intramolecular [20–22] and intermolecular coordination [23–24]. Special attention was given to bismuth···π arene interaction by us including the formation of dimers and networks [1,25–28], and recently we reported a study on the effect of intermolecular dispersion type interaction on polymorphism and phase transition of compounds of the type Ar_3_Bi (Ar = C_4_H_3_NMe, C_4_H_3_O, C_4_H_3_S, C_4_H_3_Se) [28–29].

Other state of the art examples on the formation of supramolecular assemblies via dispersion type metal···π arene intermolecular interactions [10–11] were summarized by Caracelli et al., and recently Tiekink classified this type of interaction as one of the emerging intermolecular interactions that are of particular interest to coordination chemists with regard to supramolecular chemistry [12]. However, most reports on main group metal···π interactions are based on the description of the single crystal structures and lack a profound description of the theoretical background so far. Rare examples on theoretical work about the pnictogen···π interaction were given by Frontera et al. [30–31]. While analysis of structural parameters like interatomic distances allows to assess the plausibility of certain interactions, this is exceedingly difficult and sometimes misleading for weak intermolecular interactions. Here, the accurate quantification that is possible using computational methods allows to gain a deeper understanding of which interactions are dominating. This way, a given crystal structure can be rationalized, for example, as consisting of strongly interacting dimers which themselves interact weakly with their surroundings based on the actual interaction energies. Elucidation of this is already possible at the DFT-D level of theory, if functionals with established accuracy are used, or at the DLPNO-CCSD(T) level of theory, which yields near-quantitative accuracy from first principles and can be applied to fairly large systems [32–38].

Herein, we report on intermolecular interactions with focus on bismuth···π arene interactions for the crystal structures of three polymorphs of Ph_3_Bi (**1**). For comparison the crystal structures of substituted arylbismuth compounds of the type Ar_3_Bi [Ar = C_6_H_4_-CH═CH_2_-4 (**2a**, **2b**), C_6_H_4_-OMe-4 (**3**)], Ar'_3_Bi (**4**) and Ar'_2_BiCl (**5**, Ar' = C_6_H_3_-*t*-Bu_2_-3,5) were analyzed with regard to their packing in the solid state. Electronic structure calculations were carried out on Ph_3_Bi···C_6_H_6_ and selected polymorphs of Ph_3_Bi (**1**). For this purpose, a series of electronic structure methods are applied for a model compound in order to assess the performance of different methods and to conceptually investigate and quantify the heavy main group element···π interaction present in these type of compounds. In the second part, DFT-D and DLPNO-CCSD(T) calculations are carried out for a series of molecular structures, dimers, trimers and tetramers that have been taken from the crystal structures of three selected polymorphs of compound **1**. This allows to quantify and to rationalize the balance of dispersion type interactions between bismuth and aromatic ligands as well as between the aromatic ligands itself.

## Results and Discussion

### Synthesis

So far, four polymorphs of Ph_3_Bi (**1**) have been reported in the literature [39–45], but none of these reports contains an analysis of dispersion type interactions including bismuth···π interaction in the solid state. This prompted us to have a closer look at these simple organometallic compounds. Noteworthy, the first report on the synthesis of Ph_3_Bi dates back to 1887, which was based on the reaction of sodium alloy and bromobenzene [46–47]. A more convenient synthetic route makes use of the Grignard reagent phenylmagnesium bromide and its reaction with bismuth trichloride [48]. Following this approach with slight modifications provides Ph_3_Bi with a yield of more than 80%. Crystallization from EtOH gave single crystals of the monoclinic *C*2/*c* polymorph **1a**, which was already subject of several studies including the description of its crystal structure [39–43]. Therefore, it is somewhat surprising that the bismuth···π interaction was not noted so far. We obtained polymorph **1a** upon crystallization from solution, but we isolated another polymorph **1b** by crystallization from the melt. Polymorph **1b** was obtained starting from **1a** in a temperature-dependent PXRD experiment (see Supporting Information File 1, Figure S1). The polymorph **1b** was obtained as a microcrystalline material, but Andrews and MacLellan did obtain single crystals on this orthorhombic form **1b** prior to this study [45]. Noteworthy, a phase transition of **1a** to **1b** does not occur before melting.

The latest report on a polymorph of Ph_3_Bi was made by Stammler and Neumann, which submitted the crystallographic data of a monoclinic *P*2_1_/*c* (**1c**) polymorph to the Cambridge Crystallographic Data Base [44]. In addition a monoclinic polymorph **1d** was mentioned in a brief report of Wetzel as early as 1942, but the atomic parameters were not given [39].

Following the Grignard route we were able to develop a straightforward synthetic protocol for (C_6_H_4_-CH═CH_2_-4)_3_Bi (**2**) starting from 4-bromostyrene and isolated compound **2** with 84% yield. The synthesis of **2** with very low yield is mentioned in a patent from 1964 [49], but **2** was neither fully characterized, nor was its crystal structure determined. We were able to crystallize two polymorphs of **2**, an orthorhombic form **2a** and a monoclinic form **2b**, both were obtained from iPrOH solution.

In order to develop a better understanding with regard to the effects of substituents, (C_6_H_4_-OMe-4)_3_Bi (**3**) [50–51] was prepared starting from BiCl_3_ and the corresponding organolithium reagent following a general method as reported by Wang et al. [52]. Compound **3** was obtained as colorless block-shaped crystals in yields of 83%. While our work was in progress, a crystal structure of **3** was reported by Gagnon et al. The authors confirmed the formation of **3** from the corresponding Grignard reagent and BiCl_3_ upon crystallization at 20 °C by diffusion of *n*-hexane into CH_2_Cl_2_ solution [53], but only gave a very brief description of the molecular structure.

The Ar_3_Bi compound (C_6_H_3_-*t*-Bu_2_-3,5)_3_Bi (**4**) was prepared with a yield of 73% following the Grignard route, with the intention to study the influence of very bulky substituents. Finally its chloro derivative (C_6_H_3_-*t*-Bu_2_-3,5)_2_BiCl (**5**) was synthesized in 11% yield using the organolithium derivative (C_6_H_3_-*t*-Bu_2_-3,5)Li and BiCl_3_.

**Scheme 1:**
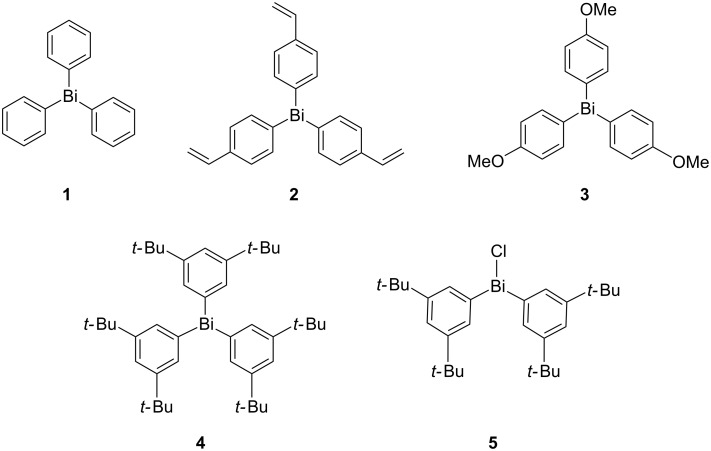
Triarylbismuth compounds, that serve as examples for the investigation of bismuth···π interactions in the solid state.

This series of compounds and polymorphs (Scheme 1) allows to deduce some general trends regarding dispersion type interactions including bismuth···π, π···π and C–H···π interactions in organobismuth compounds and therefore the crystal structures are described and discussed in the following chapter. Please note that the term C–H···π is used as a structure descriptor rather than to describe a special type of bonding. Thus we follow the criticism given by Grimme [54] and Iverson et al. [55] on the unreflected use of terms such as C–H···π, or π···π stacking previously. In most cases, these interactions rely on London dispersion forces rather than special types of bonding due to the π system.

### Crystal structures

In all of the presented compounds, the arrangement at the bismuth atom is best described as a slightly distorted trigonal pyramid, with the C–Bi–C angles significantly smaller than the tetrahedral angle, indicating that the lone pair is of mainly 6s character [41]. The Bi–C distances and C–Bi–C angles correspond to bond lengths and angles as observed for the various modifications of Ph_3_Bi [1,39,41–44] and other Ar_3_Bi compounds (Ar = Mes [56], *p*-Tolyl [57]). The molecular structures of **2a**, **2b**, **4** and **5** are illustrated in Figures S1–S4 (Supporting Information File 1), the selected bond lengths and angles are listed in the corresponding figure captions. Here, we focus mainly on the description of the supramolecular arrangements of these compounds in the solid state.

**Figure 1:**
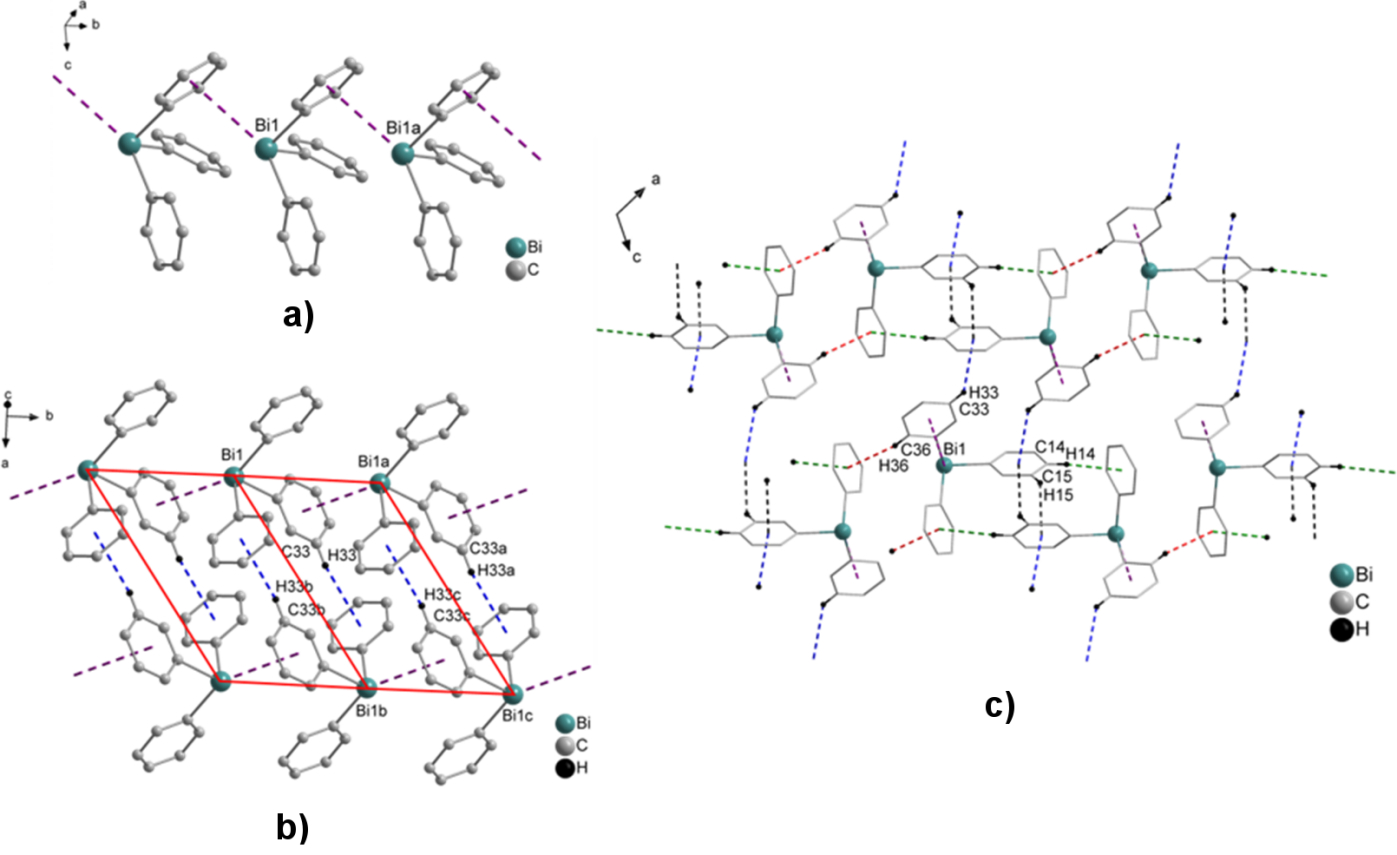
Ball and stick model of a fragment of a) the zig-zag chain of a 1D arrangement of Ph_3_Bi (**1a**). Hydrogen atoms were omitted for clarity. Selected distances [Å]: Bi1–arene_centroid_ 3.763 (violet dash line) [39–43]; b) formation of the two parallelograms connected via one edge, formed via two C–H_Ph_···π (arene_centroid_) intermolecular contacts with C33–H33_Ph_···π (arene_centroid_) 3.030 Å (blue dashed line, γ = 10.9°); c) wire and stick model of 2D and 3D networks formed via C–H_Ph_···π (arene_centroid_) intermolecular contacts C14–H14_Ph_···π (arene_centroid_) distances of 3.042 Å (green dashed line, γ = 19.5°), C15–H15_Ph_···π (arene_centroid_) distances of 2.760 Å (black dashed line, γ = 6.4°) and C36–H36_Ph_···π (arene_centroid_) distances of 2.740 Å (red dashed line, γ = 11.2°), respectively (only hydrogen atoms involved in C–H_Ph_···π (arene_centroid_) contacts are shown). Symmetry transformation: a = *x,* 1 + *y*, *z*; b = ^1^∕_2_ – *x,* ^3^∕_2_ – *y*, –*z*; c = ^1^∕_2_ – *x*, ^5^∕_2_ – *y*, –*z*.

In the literature several reports exist on the monoclinic polymorph of Ph_3_Bi (**1a**), which crystallizes in the space group *C*2/*c* [39–43]. The Bi···π arene interactions range from 3.727–3.856 Å, leading to the formation of 1D ribbons in the solid state due to Bi···π arene interactions (see zig-zag (Bi–arene_centroid_)_∞_ chain in Figure 1a). These chains are further connected via C–H_Ph_···π (arene_centroid_) intermolecular contacts with C33–H33_Ph_···π (arene_centroid_) distances of 3.030 Å (blue dashed line), γ = 10.9°, (two parallelograms connected via one edge in Figure 1b). Furthermore, two C–H_Ph_···π (arene_centroid_) intermolecular contacts are observed with C14–H14_Ph_···π (arene_centroid_) distances of 3.042 Å (green dashed line, γ = 19.5°) and C15–H15_Ph_···π (arene_centroid_) distances of 2.760 Å (black dashed line, γ = 6.4°) to give a 2D network (Figure 1c). Other additional C–H_Ph_···π (arene_centroid_) intermolecular contacts with C36–H36_Ph_···π (arene_centroid_) distances of 2.740 Å (red dashed line, γ = 11.2°) lead to the formation of a 3D network in the solid state (Figure 1c). The C–H_Ph_···arene_centroid_ contacts are shorter than 3.1 Å with an angle *γ* between the normal to the arene ring and the line defined by the H atom and the arene_centroid_ smaller than 30° [58–59].

**Figure 2:**
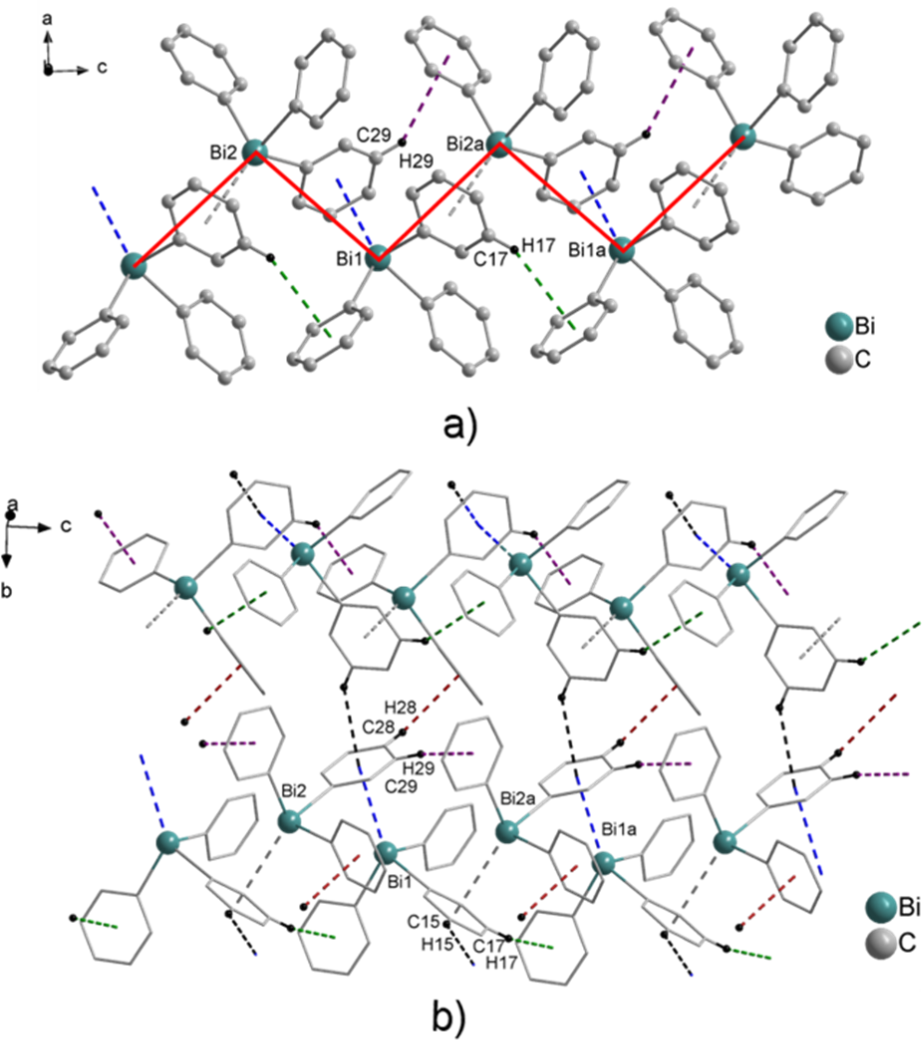
Ball and stick model of a fragment of the zig-zag type arrangement of Ph_3_Bi (**1b**) [45], view along the *b* axis. Hydrogen atoms were omitted for clarity. Selected distances [Å]: Bi1–arene_centroid_ 3.561 (blue dashed line), Bi2–arene_centroid_ 3.468 (grey dash line); a) the formation of dimers via two C–H_Ph_···π (arene_centroid_) intermolecular contacts with C17–H17_Ph_···π (arene_centroid_) 3.083 Å (green dashed line, γ = 9.5°) and C29–H29_Ph_···π (arene_centroid_) 3.097 Å (γ = 16.4°); b) wire and stick model of a 2D network build via additional C–H_Ph_···π (arene_centroid_) intermolecular contacts with C15–H15_Ph_···π (arene_centroid_) 3.034 Å (black dashed line), (γ = 8.4°) and C28–H28_Ph_···π (arene_centroid_) 2.890 Å (brown dashed line, γ = 13.5°), (only hydrogen atoms involved in C–H_Ph_···π (arene_centroid_) contacts are shown). Symmetry transformations: a = *x*, *y*, 1 + *z*.

The polymorph **1b** crystallizes in the orthorhombic space group *Pna*2_1_ [45]. The crystal structure of polymorph **1b** shows two different bismuth atoms in the unit cell, each of them being involved in Bi···π arene intermolecular interactions, with Bi2–arene_centroid_ 3.468 Å (grey dashed line in Figure 2) and Bi1–arene_centroid_ 3.561 Å (blue dashed line in Figure 2), thus resulting in zig-zag type 1D ribbons. In addition C–H_Ph_···π (arene_centroid_) intermolecular contacts with C17–H17_Ph_···π (arene_centroid_) 3.083 Å (γ = 9.5°) and C29–H29_Ph_···π (arene_centroid_) 3.097 Å, (γ = 16.4°, green and purple dashed line in Figure 2a, respectively) complement the structure. The ribbons are connected via two additional C–H_Ph_···π (arene_centroid_) intermolecular contacts with C15–H15_Ph_···π (arene_centroid_) 3.034 Å (black dashed line, γ = 8.4°) and C28–H28_Ph_···π (arene_centroid_) 2.890 Å (brown dashed line, γ = 13.5°) and lead to the formation of a 2D network (Figure 2b).

**Figure 3:**
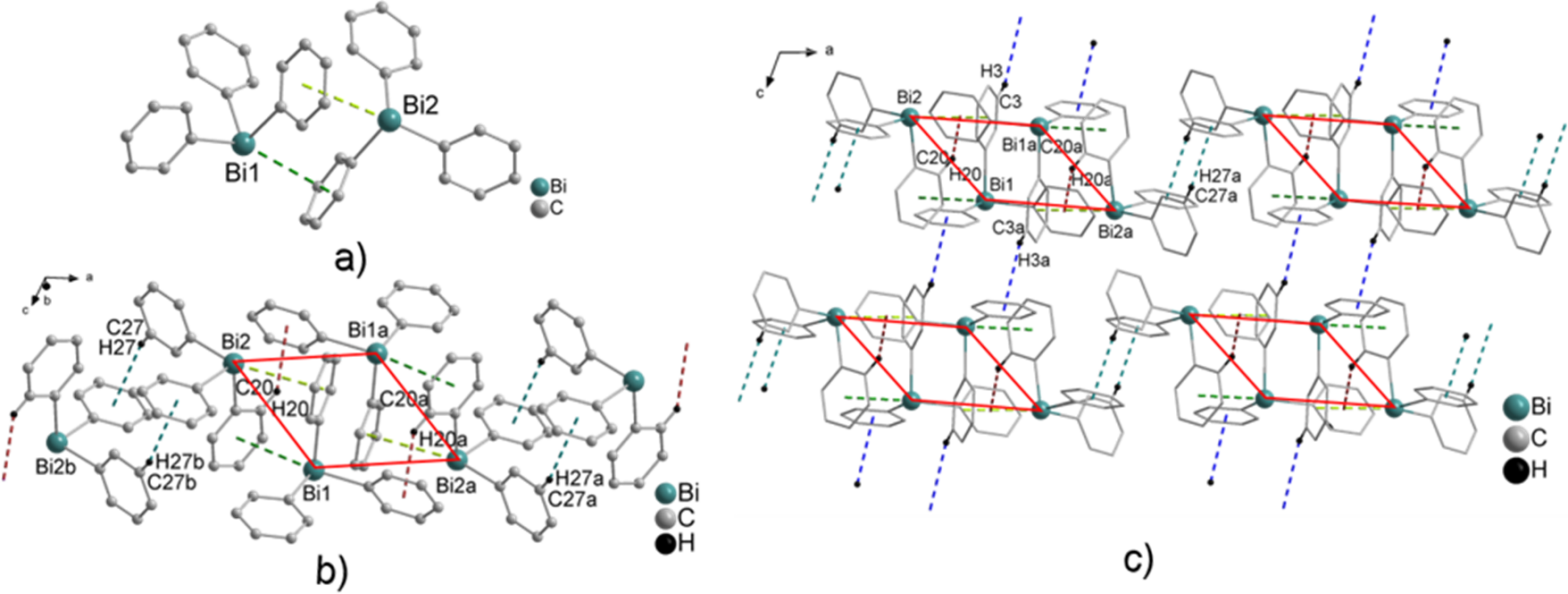
Ball and stick model of Ph_3_Bi (**1c**) showing: a) non-centrosymmetric dimers formed via two Bi···π arene intermolecular contacts. Hydrogen atoms were omitted for clarity. Selected distances [Å]: Bi1–arene_centroid_ 3.787 (green dashed line), Bi2–arene_centroid_ 3.939 (lime dash line) [1,44]; b) the formation of a four-membered ring of a 1D layer build via two C–H_Ph_···π (arene_centroid_) intermolecular contacts with C20–H20_Ph_···π (arene_centroid_) 2.801 Å (dark red dashed line, γ = 12.3°) and C27–H27_Ph_···π (arene_centroid_) 2.763 Å (teal dashed line, γ = 12.6°); c) the formation of a 3D network build via additional C–H_Ph_···π (arene_centroid_) intermolecular contacts with C3–H3_Ph_···π (arene_centroid_) 3.037 Å (blue dashed line, γ = 7.9°), (only hydrogen atoms involved in C–H_Ph_···π (arene_centroid_) contacts are shown). Symmetry transformation: a = 2 – *x,* –*y*, 1 – *z*; b = 1 – *x,* –*y*, 1 – *z*.

Another polymorph of Ph_3_Bi (**1c**) was reported by Neumann and co-workers in a CSD communication. The polymorph **1c** crystallizes in the monoclinic space group *P*2_1_/*c* [1,44]. The crystal structure of polymorph **1c** reveals the formation of non-centrosymmetric dimers in the solid state, which are formed via two Bi···π arene intermolecular contacts (Figure 3a). These distances amount to Bi1–arene_centroid_ 3.787 Å (green dashed line) and Bi2–arene_centroid_ 3.939 Å (lime dashed line). A closer look at the crystal structure of polymorph **1c** reveals that the dimeric units self-assemble via C–H_Ph_···arene_centroid_ contacts, which leads to the formation of centrosymmetric units, based on C–H_Ph_···π (arene_centroid_) intermolecular contacts (four-membered ring in Figure 3b), with C20–H20_Ph_···π (arene_centroid_) 2.801 Å (dark red dashed line, γ = 12.3°) and C27–H27_Ph_···π (arene_centroid_) 2.763 Å (teal dashed line, γ = 12.6°), (1D layers in Figure 3b), respectively. Additionally, the 1D layers are connected via two C–H_Ph_···π (arene_centroid_) intermolecular contacts, with C3–H3_Ph_···π (arene_centroid_) 3.037 Å (blue dashed line, γ = 7.9°) to give a 3D network in the solid state (Figure 3c).

It is worth to note that Wetzel has reported another crystal structure of Ph_3_Bi (**1d**) in 1942, which crystallizes in the triclinic space group 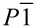 [39]. Unfortunately, the crystal structure of the latter could not be analyzed by us due to the lack of atomic parameters.

#### (C_6_H_4_-CH═CH_2_-4)_3_Bi (**2**)

**Figure 4:**
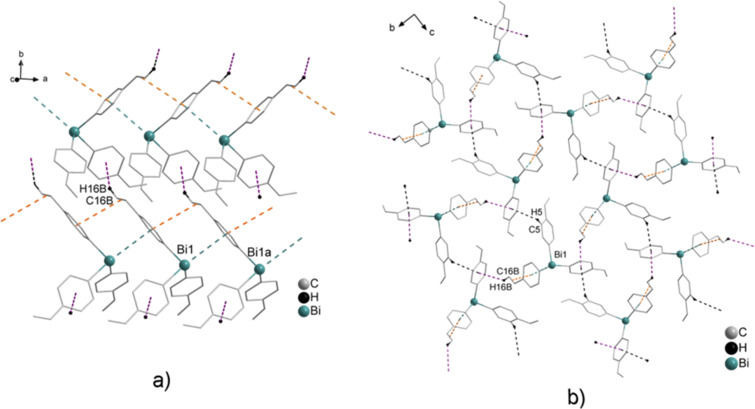
Wire and stick representation of (C_6_H_4_-CH═CH_2_-4)_3_Bi (**2a**) showing: a) zig-zag chains of 1D ribbons formed via Bi···π arene intermolecular contacts (Bi1–arene_centroid_ 3.835 Å), accompanied by π_Ph_···π_vinyl_ contacts of 3.798 Å and the formation of 2D network via C16B–H16B_vinyl_···π (arene_centroid_) 2.980 Å (violet dashed line, γ = 14.5°); b) the formation of the 3D network via C5–H5···π (arene_centroid_) 3.094 Å (black dashed line, γ = 26.0°, only hydrogen atoms involved in C–H_Ph_···π (arene_centroid_) contacts are shown). Symmetry transformations: a = 1 + *x*, *y*, *z*; b = –1 + *x*, *y*, *z*.

**Figure 5:**
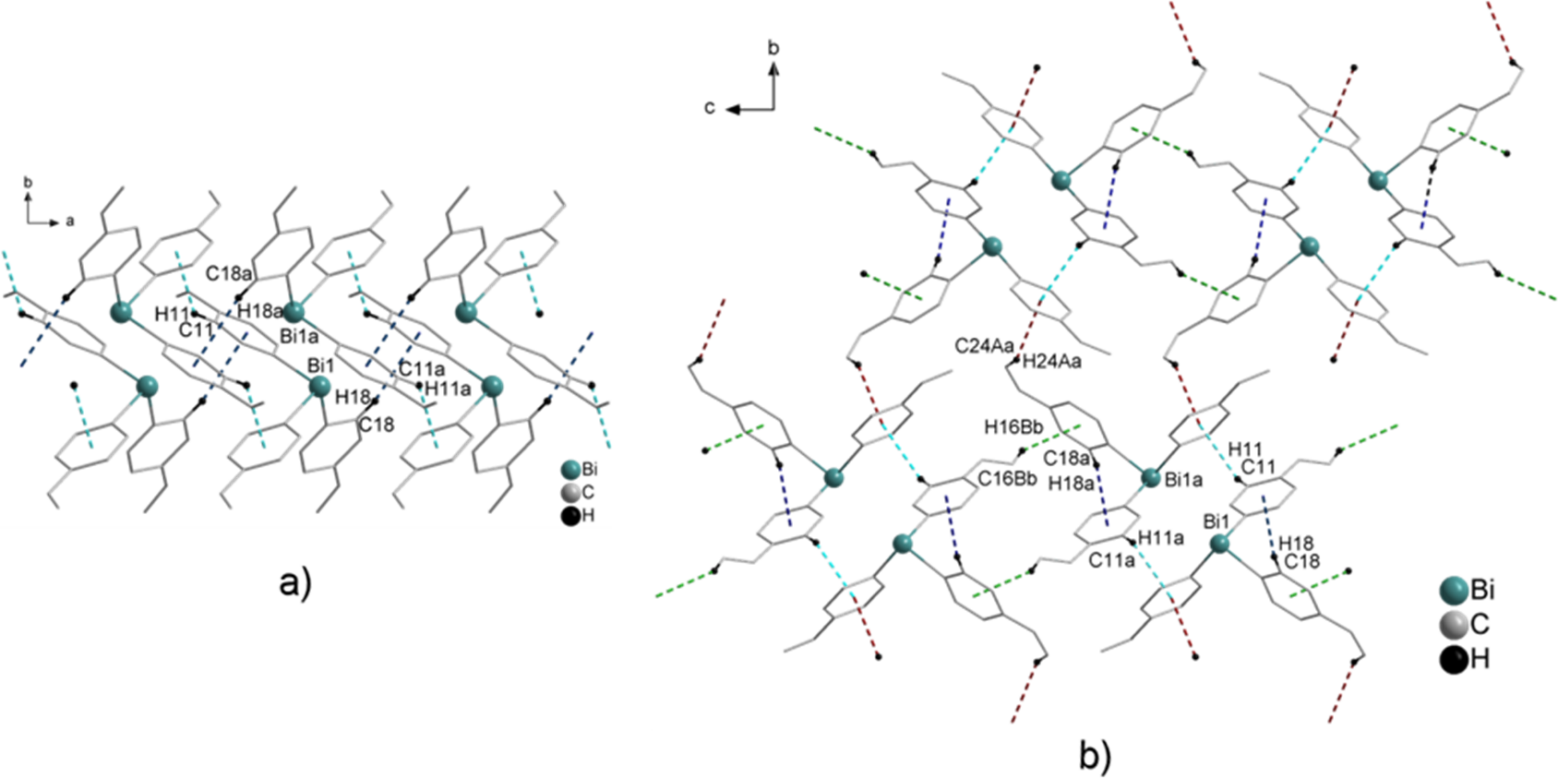
Wire and stick model of (C_6_H_4_-CH═CH_2_-4)_3_Bi (**2b**) showing: a) the formation of 1D ribbons build via two C–H_Ph_···π (arene_centroid_) intermolecular contacts with C11–H11_Ph_···π (arene_centroid_) 2.817 Å (cyan dashed line, γ = 8.5°) and C18–H18_Ph_···π (arene_centroid_) 2.940 Å (dark blue dashed line, γ = 10.0°); b) 2D network formed via C16Bb–H16Bb_vinyl_···π (arene_centroid_) 2.980 Å (γ = 25.0°) and a 3D network formed via C24Ac–H24Ac_vinyl_···π (arene_centroid_) of 2.904 Å (γ = 7.0°), (only hydrogen atoms involved in C–H_Ph_···π (arene_centroid_) contacts are shown). Symmetry transformation: a = 1 – *x*, 1 – *y*, 1 – *z*; b = 1 + *x*, *y*, 1 + *z*; Bi···Bi of 4.046 Å.

Crystallization of (C_6_H_4_-CH═CH_2_-4)_3_Bi (**2**) from iPrOH solution gave pale yellow crystals, which either form needles or rarely a more compact morphology. Both types of crystals of **2** were suitable for single crystal X-ray diffraction analysis and revealed the formation of two polymorphs **2a** (colorless acicular crystals) and **2b** (light yellow block-shaped crystals) in the solid state. Polymorph **2a** crystallizes in the orthorhombic space group *P*2_1_2_1_2_1_ (Figure 4), while the crystal structure analysis of polymorph **2b** revealed the monoclinic space group *P*2_1_/*c* (Figure 5).

For **2a** Bi···π arene interactions between the bismuth atom and the aryl ring of the neighboring molecule are deduced, which leads to the formation of zig-zag Bi–arene_centroid_ chains along the crystallographic axis (1D ribbons in Figure 4a). The Bi–arene_centroid_ distance amounts at 3.835 Å (Σ_vdW_ (Bi–C) = 3.77–4.31 Å), which corresponds to the distances of 3.47 to 3.96 Å, as reported for the polymorphs of Ph_3_Bi [1,39,41–44]. The overall crystal structure of **2a** is very similar to the monoclinic *C*2/*c* modification of Ph_3_Bi (**1a**) [41–43]. Additionally, short π···π distances were observed between one of the vinyl groups and the aryl ligand, with a distance from the centroid of the aromatic ring to the midpoint of the C*═*C double bond of 3.798 Å. The angle to the plane through the aryl ligand of the neighboring molecule amounts at 13.1° and thus a nearly linear arrangement between a bismuth atom, an aryl ligand, and the vinyl group with an angle of 171.8° is observed (Figure 4a).

In **2a** each Bi···π arene contact is accompanied by a π_Ph_···π_vinyl_ contact (orange dashed line in Figure 4a). The 1D chains are connected via C–H_vinyl_···π (arene_centroid_) intermolecular contacts with C16B–H16B_vinyl_···π (arene_centroid_) 2.980 Å (violet dashed line, γ = 14.5°) to form a 2D network (Figure 4a). Additional C–H_Ph_···π (arene_centroid_) intermolecular contacts with C5–H5···π (arene_centroid_) of 3.094 Å (black dashed line, γ = 26.0°) lead to the formation of a 3D network in the solid state (Figure 4b). By contrast, the crystal structure of **2b** did not show any Bi···π arene interaction, and it reveals only the presence of C–H_Ph_···arene_centroid_ contacts. Two sorts of C–H_Ph_···π (arene_centroid_) intermolecular contacts, with C11–H11_Ph_···π (arene_centroid_) 2.817 Å (cyan dashed line, γ = 8.5°) and C18–H18_Ph_···π (arene_centroid_) 2.940 Å (dark blue dashed line, γ = 10.0°) are observed (Figure 5a). Furthermore, additional C–H_vinyl_···π (arene_centroid_) intermolecular contacts with C16Bb–H16Bb_vinyl_···π (arene_centroid_) 2.960 Å (green dashed line, γ = 25.0°) lead to the formation of a 2D network, while other intermolecular contacts with C24Ac–H24Ac_vinyl_···π (arene_centroid_) of 2.904 Å (brown dashed line, γ = 7.0°) result in the formation of a 3D network (Figure 5b). The Bi···Bi contacts are considerably shorter than the sum of the van der Waals radii of bismuth atoms (Bi···Bi contacts of 4.046 Å; Σ*r*_vdW_(Bi, Bi) 4.08–5.14 Å)) [60–62] and are in good agreement with the ones reported recently in a theoretical study by Jansen and co-workers who discussed dispersion type Bi···Bi interactions in the context of structure formation in R_3_Bi compounds (Bi···Bi contacts vary between 4.015 and 4.059 Å) [63].

#### (C_6_H_4_-OMe-4)_3_Bi (**3**)

**Figure 6:**
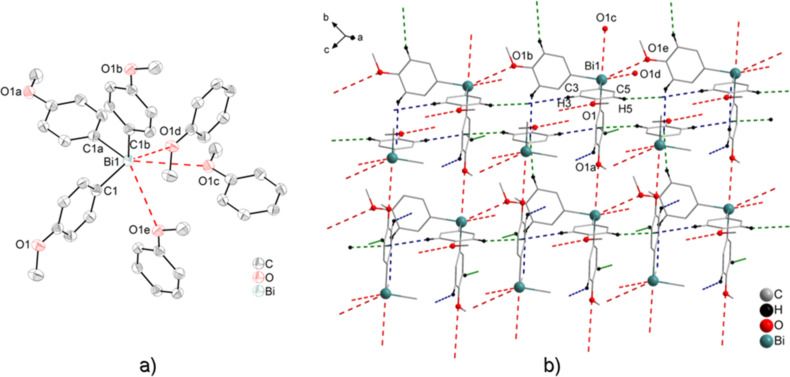
Molecular structure of (C_6_H_4_-OMe-4)_3_Bi (**3**) showing: a) Thermal ellipsoids that are set at 50% probability level. Hydrogen atoms were omitted for clarity. Symmetry transformations: a = –*x* + *y* + *2*, –*x* + 1, *z*; b = – *y* + 1, *x* – *y* – 1, *z*; c = ^7^⁄_3_ – *x* + *y*, ^5^⁄_3_ – *x*, –^1^⁄_3_ + *z*; d = ^2^⁄_3_ + *x*, –^1^⁄_3_ + *y*, –^1^⁄_3_ + *z*; e = ^4^⁄_3_ + *y*, –^4^⁄_3_ + *x* – *y*, –^1^⁄_3_ + *z*. Selected bond lengths [Å]: Bi1–C1 2.248(3), Bi1–C1a 2.248(3), Bi1–C1b 2.248(3), Bi1–O1c 3.781, Bi1–O1d 3.781, Bi1–O1e 3.781. Selected bond angles [°]: C1–Bi1–C1a 93.7(11), C1–Bi1–C1b 93.7(11), C1a–Bi1–C1b 93.7(11), O1c–Bi1–O1d 69.6, O1c–Bi1–O1e 69.6, O1d–Bi1–O1e 69.6; b) wire and stick representation of a 3D network built via Bi···O intermolecular interactions and via two C–H_Phyl_···π (arene_centroid_) intermolecular contacts C3–H3_Ph_···π (arene_centroid_) 3.005 Å (blue dashed line, γ = 14.7°) and C5–H5_Ph_···π (arene_centroid_) 2.845 Å (green dashed line, γ = 11.6°).

(C_6_H_4_-OMe-4)_3_Bi (**3**) crystallized from CHCl_3_ in the form of colorless cube-shaped or block-shaped crystals which were suitable for single crystal X-ray structure analysis. Compound **3** crystallizes in the trigonal space group 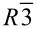 (Figure 6). While our work was in progress, Gagnon and co-workers reported the crystal structure of **3**, which exhibits very similar lattice parameters [64]. However, the packing structure has not been discussed in detail and thus its description is given here.

A closer look at the bismuth environment reveals that for the molecular structure of **3** the bismuth atom might be described as six-coordinated being surrounded by six 4-methoxyphenyl groups. Three of them are bonded covalently to bismuth with Bi–C of 2.248(3) Å and three units are bonded via weaker Bi···O interactions, which have identical values (Bi···O 3.781 Å), finally leading to a [3 + 3] coordination (van der Waals radii Σ_vdW_ (Bi,O) = 3.57–4.09 Å) [60–62]. The 4-methoxyphenyl moieties are pointing via the oxygen atoms to the bismuth atom, which actually hinders the formation of bismuth Bi···π arene interactions. A similar coordination environment was observed in the case of the two polymorphs of (2-C_4_H_3_S)_3_Bi, where three thienyl molecules of the neighboring molecules interact with Ar_3_Bi [29]. The oxygen atoms of the methoxy groups each interact with the bismuth atom of a neighboring molecule in a way that each bismuth atom interacts with three oxygen atoms of different neighbors. In the resulting three-dimensional structure, one molecule of **3** interacts with six other molecules (Figure 6b). Similar Bi···O interactions are also found in tris(2-methoxyphenyl)- and tris(2,4-dimethoxyphenyl)bismuthine [65]. However, the coordination sphere of the bismuth atoms in these compounds is complemented intramolecularly through the methoxy groups in the *ortho* position (Bi···O of 3.05 Å and 3.15 Å) [65].

The crystal structure of **3** did not show any Bi···π arene interaction, but reveals the presence of C–H_Ph_···arene_centroid_ contacts. These intermolecular C–H_Ph_···π (arene_centroid_) contacts amount to C3–H3_Ph_···π (arene_centroid_) 3.005 Å (blue dashed line, γ = 14.7°) and C5–H5_Ph_···π (arene_centroid_) 2.845 Å (green dashed line, γ = 11.6°, Figure 6b).

#### (C_6_H_3_-*t*-Bu_2_-3,5)_3_Bi (**4**) and (C_6_H_3_-*t*-Bu_2_-3,5)_2_BiCl (**5**)

**Figure 7:**
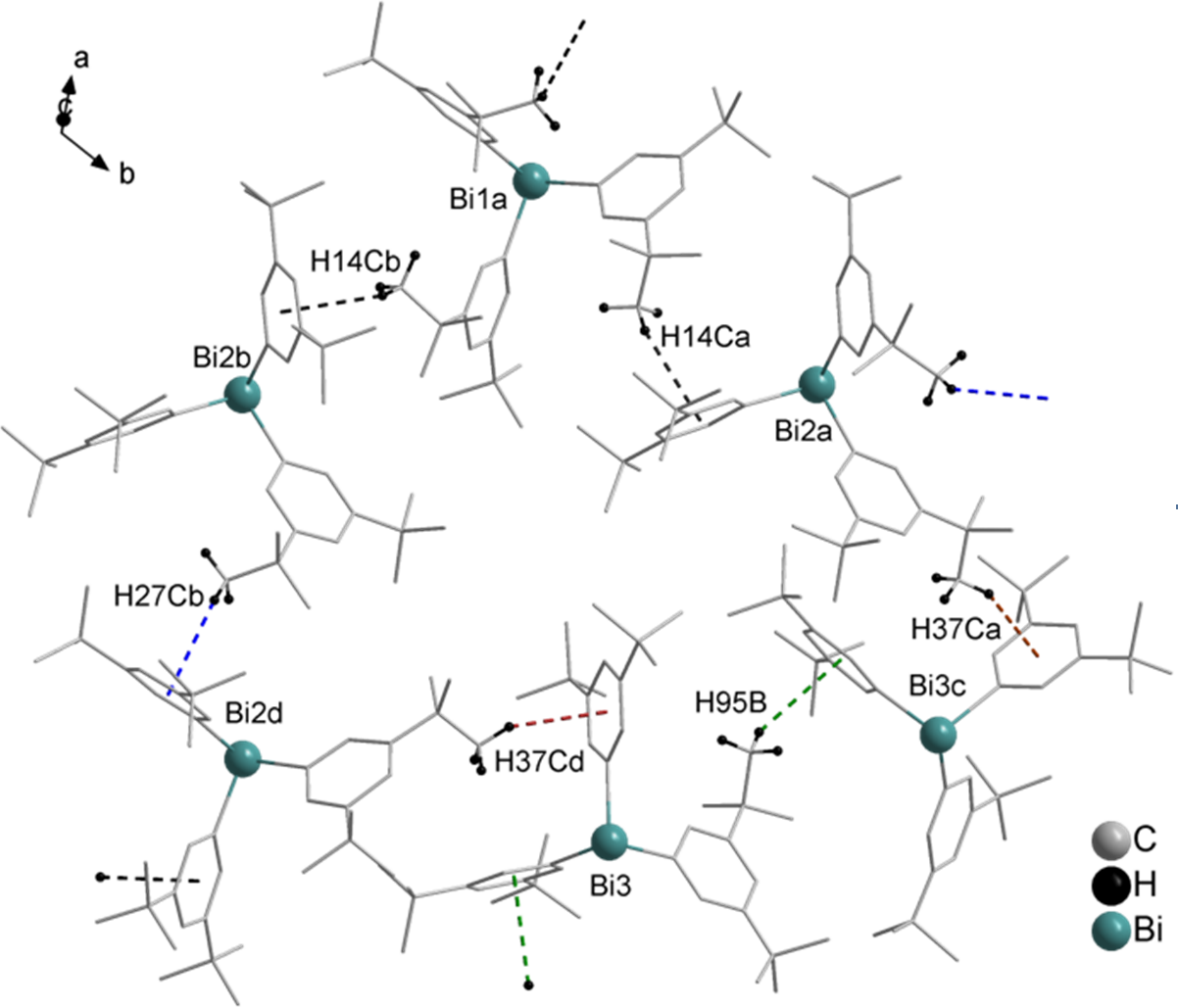
Wire and stick model of (C_6_H_3_-*t*-Bu_2_-3,5)_3_Bi (**4**) showing a 3D network build via four C–H*_t-_*_Bu_···π (arene_centroid_) intermolecular contacts with C14–H14Ca*_t-_*_Bu_···π (arene_centroid_) 2.877 Å (black dashed line, γ = 10.7°), C27–H27Cb*_t-_*_Bu_···π (arene_centroid_) 2.884 Å (blue dashed line, γ = 7.8°), C37–H37Ca*_t-_*_Bu_···π (arene_centroid_) 3.012 Å (brown dashed line, γ = 11.5°) and C95–H95Bc*_t-_*_Bu_···π (arene_centroid_) 3.002 Å (green dashed line, γ = 6.5°), (only hydrogen atoms involved in C–H*_t-_*_Bu_···π (arene_centroid_) contacts are shown). Symmetry transformation: a = *x*, *y*, 1 + *z*; b = 1 – *y*, 1 + *x* – *y*, 1 + *z*; c = 1 + *x* – *y*, 1 + *x*, ^1^⁄_2_ + *z*; d = –1 + *y*, –*x* + *y*, ^1^⁄_2_ + *z*.

Colorless single crystals suitable for X-ray crystallography were isolated upon crystallization from a CH_2_Cl_2_ solution at ambient temperature (for **4**) and at −28 °C (for **5**). Compounds **4** and **5**·2CH_2_Cl_2_ crystallize in the hexagonal space group *P*6_3_ and orthorhombic space group *Pna*2_1_, respectively. The crystal structure of compound **4** does not exhibit any Bi···π arene interactions, but shows four C–H*_t-_*_Bu_···π (arene_centroid_) intermolecular contacts, with C14–H14C1a*_t-_*_Bu_···π (arene_centroid_) 2.877 Å (black dashed line, γ = 10.7°), C27–H27Cb*_t-_*_Bu_···π (arene_centroid_) 2.884 Å (blue dashed line, γ = 7.8°), C37–H37Ca*_t-_*_Bu_···π (arene_centroid_) 3.012 Å (brown dashed line, γ = 11.5°) and C95–H95Bc*_t-_*_Bu_···π (arene_centroid_) 3.002 Å (green dashed line, γ = 6.5°) in Figure 7, respectively. The presence of bismuth···π arene interactions could not be observed most probably due to the bulky *t*-Bu groups attached to the aryl ligands, which hinder the interactions of the bismuth atom with the aryl ligands of the neighboring molecules (Figure 7).

**Figure 8:**
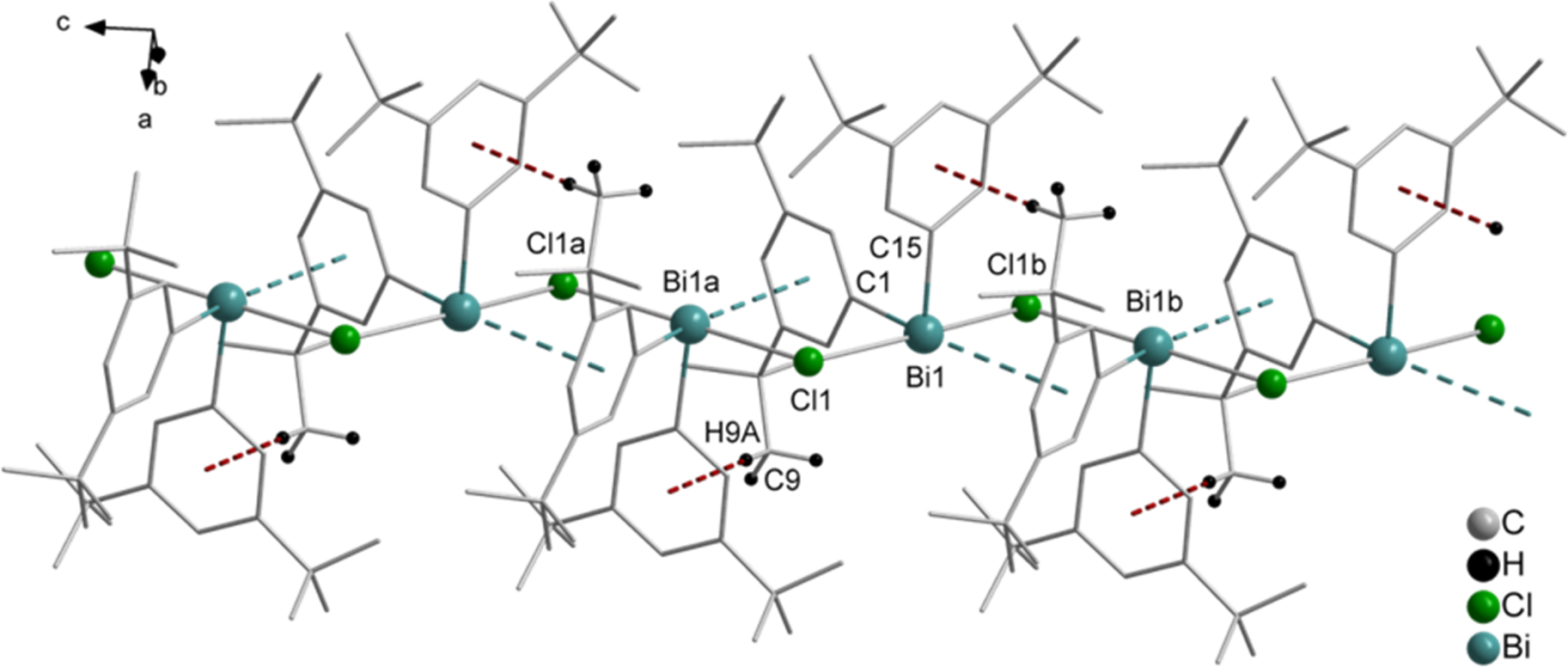
Wire and stick model of (C_6_H_3_-*t*-Bu_2_-3,5)_2_BiCl (**5**) showing a fragment of the 1D arrangement, view along the *b* axis. Symmetry transformations: a = 2 – *x*, –*y*, ^1^∕_2_ + *z*; b = 2 – *x*, –*y*, –^1^∕_2_ + *z*. Selected bond lengths and distances [Å]: Bi1–Cl1 2.811(7), Bi1–Cl1b 2.805(7), Bi1a–Cl1 2.805(7), Bi1–arene_centroid_ 3.725. Selected bond angles [°]: C15–Bi1–Cl1 90.9(10), C15–Bi1–Cl1b 92.7(9), C(15)–Bi(1)–arene_centroid_ 89.7, Cl1–Bi1–Cl1b 171.5(17), Bi1–Cl1–Bi1a 112.6(19). C–H*_t-_*_Bu_···π (arene_centroid_) intermolecular contacts, C9–H9A*_t-_*_Bu_···π (arene_centroid_) 2.662 Å (brown dashed line), (γ = 7.4°).

The crystal structure analysis of **5** revealed intermolecular donor acceptor Bi···Cl interactions of Bi1–Cl1b 2.805(7) Å, which are accompanied by Bi···π arene contacts of Bi1–arene_centroid_ 3.725 Å. This arrangement results in a sort of intermolecular pincer-type coordination of the bismuth atom, and thus in the formation of a 1D chain in the solid state (Figure 8). Due to the Bi···π arene interactions, the local geometry of the bismuth atom becomes distorted square pyramidal with one carbon atom of the (C_6_H_3_-*t*-Bu_2_-3,5) ligand in the axial positions and the two chlorine atoms, another carbon atom of the aryl ligand and the arene_centroid_ placed in the equatorial positions, describing the basal plane. This is reflected in the bond angles of C15–Bi1–arene_centroid_ 89.7°, C15–Bi1–Cl1 90.9(10)°, C15–Bi1–Cl1b 92.7(9)°, and C1–Bi1–C15 92.4(10)°. Besides these contacts, the crystal structure of **5** revealed short C–H*_t-_*_Bu_···π (arene_centroid_) contacts for C9–H9A*_t-_*_Bu_···π (arene_centroid_) 2.662 Å (brown dashed line, γ = 7.4°).

As shown in this section, the crystal structures of **1**–**5** described above revealed the presence of London dispersion type interactions in the solid state, with bismuth acting as dispersion energy donor (DED) only in some cases. In the absence of strong donor acceptor type interactions a competition between the different types of dispersion interactions (Bi···π, π···π or C–H···π) is observed and thus leads to different structural features in the solid state (Table 1). Here the question arises how important and how large these interactions are and whether any type of interaction is dominating. For this reason computational studies have been performed with the focus on the crystal structures of three polymorphs of Ph_3_Bi (**1**), which revealed either the formation of non-centrosymmetric dimers as basic building block, or the formation of 1D ribbons (i.e., zig-zag type). Both are based on bismuth···π arene interactions: the formation of 2D networks is built up via C–H_Ph_···π intermolecular contacts of T-shape. Noteworthy, polymorph **2a** showed bismuth···π and π···π interactions leading to 1D ribbons in the solid state, while **2b** did not reveal Bi···π interactions. Thus, it is concluded that Bi···π, π···π and C–H···π interactions must be of similar strength. Similar to the situation of **2b**, compound **4** did not show any bismuth···π arene interactions, but also exhibits C–H*_t-_*_Bu_···π intermolecular contacts. In compounds **3** and **5** intermolecular Bi···O and Bi···Cl bonds are dominating.

**Table 1:** Various intermolecular distances observed in the crystal structures of arylbismuth compounds.

	Bi···π intermolecular distances	π···π intermolecular distances	C–H···π intermolecular distances	Bi···O/Cl intermolecular distances	structural features

Ph_3_Bi (**1**)					
polymorph **1a**	3.763 Å		3.030 Å 3.042 Å 2.760 Å 2.740 Å		3D network
polymorph **1b**	3.468 Å 3.561 Å		3.083 Å 3.097 Å 3.034 Å 2.890 Å		2D network
polymorph **1c**	3.787 Å 3.939 Å		2.801 Å 2.763 Å 3.037 Å		3D network
(C_6_H_4_-CH═CH_2_-4)_3_Bi (**2**)					
polymorph **2a**	3.835 Å	3.798 Å			1D ribbons
polymorph **2b**			2.817 Å 2.940 Å		1D ribbons
(C_6_H_4_-OMe-4)_3_Bi (**3**)			3.005 Å 2.845 Å	3.781 Å	3D network
(C_6_H_3_-*t*-Bu_2_-3,5)_3_Bi (**4**)			2.877 Å 2.884 Å 3.012 Å 3.002 Å		3D network
(C_6_H_3_-*t*-Bu_2_-3,5)_2_BiCl (**5**)	3.725 Å		2.662 Å	2.811 Å 2.805 Å	1D ribbons

### Electronic structure calculations on selected polymorphs of Ph_3_Bi

In order to assess the role of dispersion interactions for the existence of structural features in compounds including Bi···π interactions, we focus our study on the wealth of structural information for BiPh_3_ (compound **1**). Note that various structural motifs present in the polymorphs of compound **1** can be also found in polymorphs of compound **2**.

We will proceed as follows: First, an idealized model compound is studied such that the basic Bi···π interaction can be classified in comparison to other systems previously studied. Then, we will turn to the crystal structures of polymorph **1a**, **1b**, and **1c** and investigate each polymorph in terms of intermolecular interactions to assess which influences are decisive for structure formation. For this purpose, several tetrameric units have been extracted from the crystal structure for each polymorph. This way, all relevant intermolecular interactions in the solid state can be studied based on monomer distortion, intermolecular interactions of representative dimers and the interaction of one molecule with several of its neighbors.

### Distance scan for the idealized BiPh_3_···benzene complex

**Figure 9:**
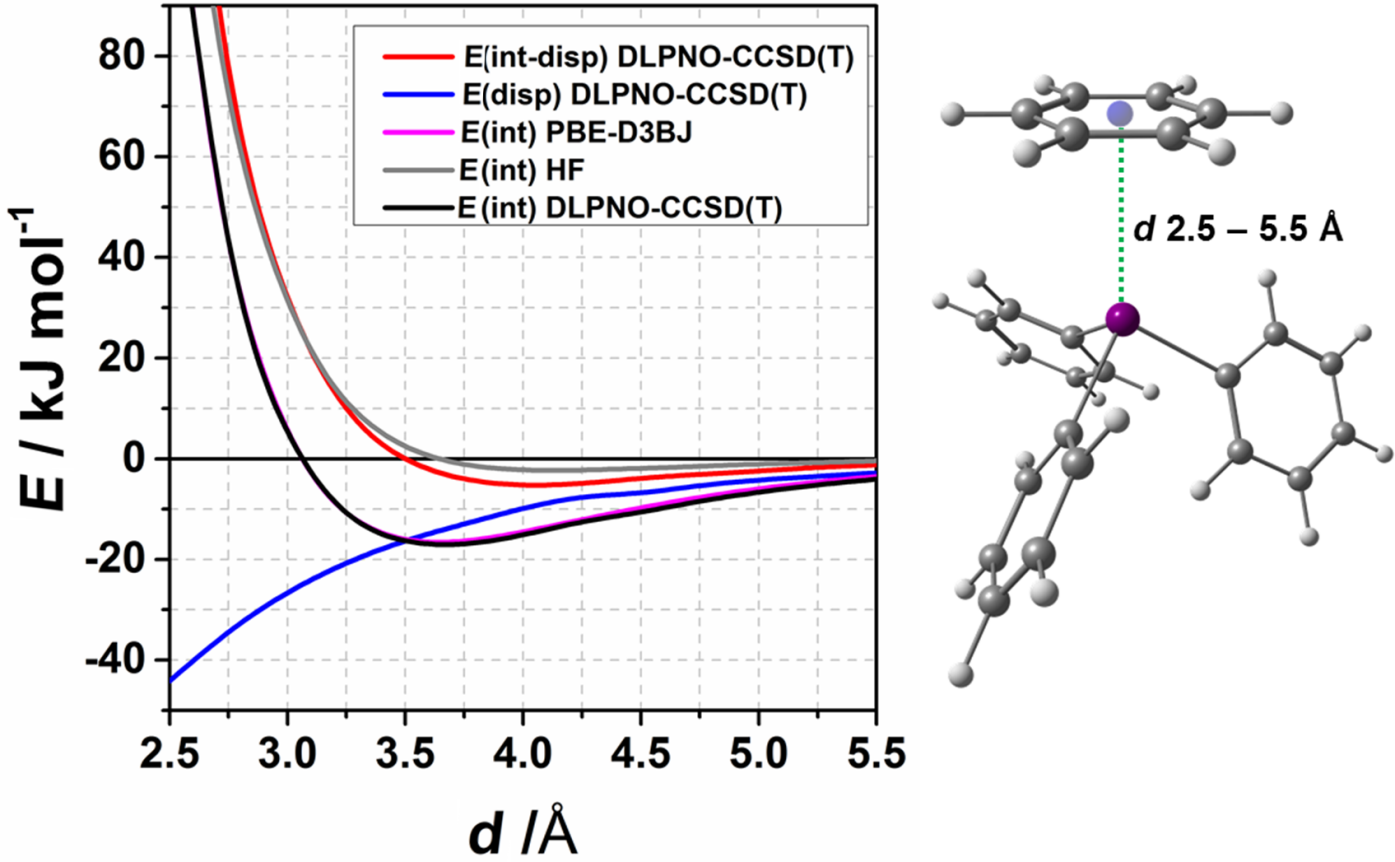
Computed interaction potentials of the distance scan for the idealized BiPh_3_–benzene complex. *E*(int) denotes the interaction energy obtained at the particular level of theory, *E*(disp) denotes the dispersion energy, *E*(int-disp) – the interaction energy without the dispersion contribution.

In a previous study on the nature of Bi···π arene interactions in various benzene complexes with BiR_3_ (with R = Me, OMe, and Cl), we found that interaction energies for this type of compounds range from −10 kJ mol^−1^ to −40 kJ mol^−1^. The character of the interaction varies from purely dispersive for BiMe_3_ to dispersive with pronounced donor–acceptor character in case of Bi(OMe)_3_ and BiCl_3_ complexes [66–67]. In order to assess the nature of the interaction in the BiPh_3_···π complexes (compound **1**), rigid potential energy surface scans for the idealized BiPh_3_–benzene complex were performed at the PBE-D3 and DLPNO-CCSD(T) level of theory. The idealized structure was constructed in order to disentangle pure Bi···π arene interaction from the influence of substituents. The interaction potential curve is shown in Figure 9. The distance scans obtained at the PBE-D3 level of theory are in good agreement with the DLPNO-CCSD(T) results which shows that using the PBE-D3 functional is a cost efficient alternative to the DLPNO-CCSD(T) method. The minimum on the DLPNO-CCSD(T) potential energy curve estimated by interpolation corresponds to a distance of 3.66 Å and to −17 kJ mol^−1^. Note that the Bi···π arene contact minimum distance is shorter than for **1a**, **1c**, and **2** but slightly longer than for **1b** (see Table 1). The curve for the interaction energy without dispersion contribution (Figure 9, *E*(int-disp)) is slightly attractive.

**Figure 10:**
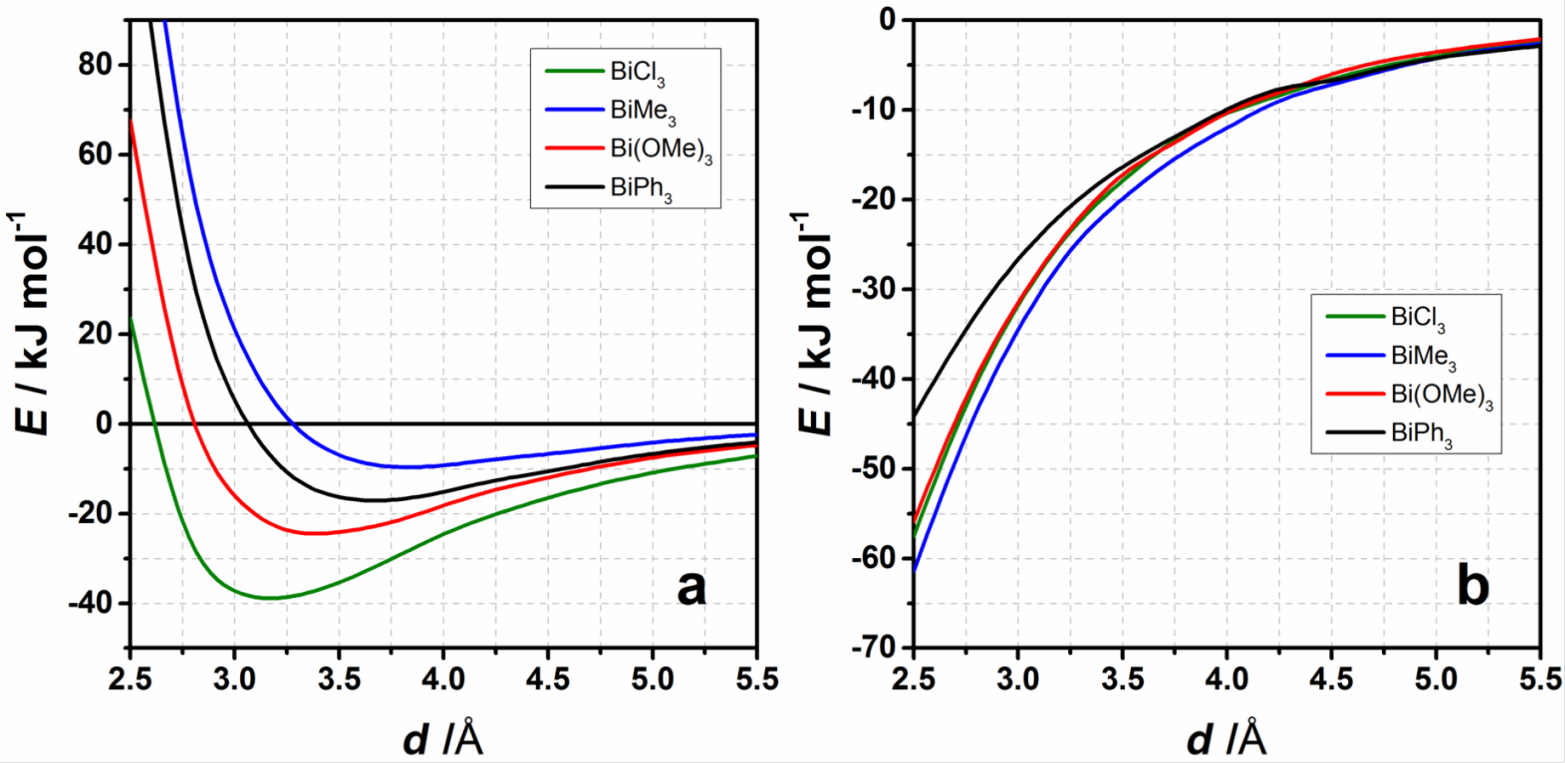
a) The BiPh_3_ potential energy curve for idealized interaction structures compared to the interaction energy curves for the series of compounds BiR_3_···C_6_H_6_ with R = Me, OMe, Cl obtained at the DLPNO-CCSD(T) level of theory. b) Dispersion energy contributions according to LED (DLPNO-CCSD(T)) for the distance scans shown in Figure 9.

The interaction energy of the BiPh_3_ complex is higher than the interaction energy obtained for BiMe_3_ but smaller than for Bi(OMe)_3_ (see Figure 10a), however, the dispersion contribution to the interaction energy in the BiPh_3_ complex (see Figure 10b) is comparable to the dispersion contributions in other BiR_3_–benzene complexes. This implies that the character of the interaction in the BiPh_3_–benzene complex is closer to that of BiMe_3_ rather than to Bi(OMe)_3_ and that the interaction is dominated by dispersion with minor contribution of donor–acceptor character.

### Computational description of the polymorphs of BiPh_3_

**Figure 11:**
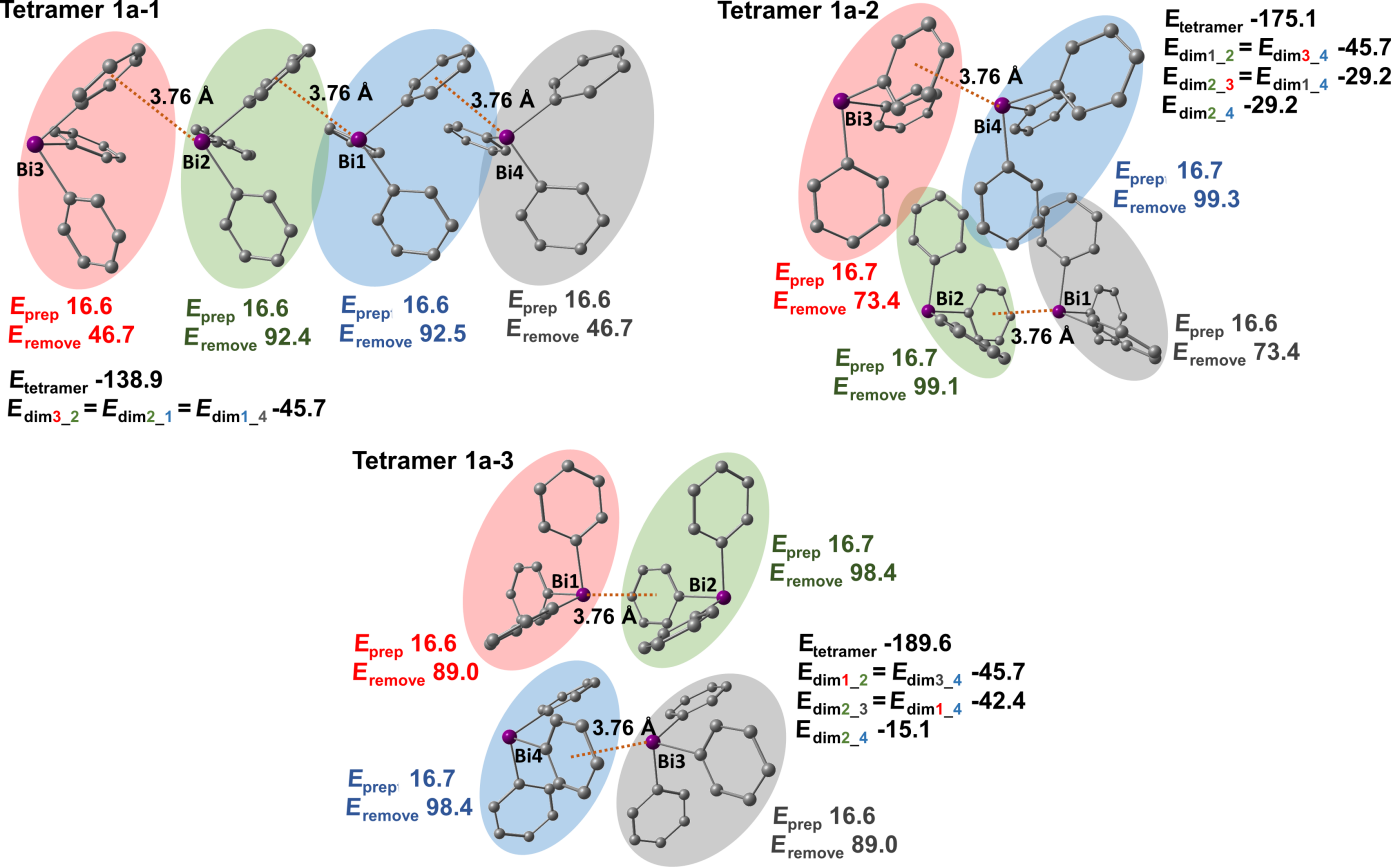
Structures of studied BiPh_3_ tetramers extracted from the crystal structure of polymorph **1a**. *E*_tetramer_ indicates the interaction energy computed for a given tetramer; *E*_dim_ is the interaction energy computed for a specific dimer; *E*_prep_ is the distortion energy of a monomer in the crystal structure; *E*_remove_ denotes the energy required to remove a monomer from the tetramer. All energies were computed at the PBE-D3/def2-TZVP and are given in kJ mol^−1^. Numbering of monomers is the same as numbering of bismuth atoms within the specific tetramer. Hydrogen atoms in tetramers were omitted for the clarity.

In this section we address the question which factors influence the structure in solid state for BiPh_3_ and what possible intermolecular interactions within the polymorphs of compound **1** are. As mentioned already earlier several tetrameric units of polymorphs of compound **1** were chosen in order to obtain a simplified description of the crystal structure. These tetramers contain the information on the different intermolecular interactions present in the solid state of BiPh_3_. The structures of the studied tetramers are shown in Figures 11, 13, and 15. Subsequently, tetramers were divided into dimeric units that represent specific intermonomer interactions, and can be considered as building blocks of the bulk. Monomer distortion energies (geometry preparation; denoted as *E*_prep_ in Figures 11, 13, and 15) were computed in order to gain knowledge on crystal packing effects. The distortion energy is obtained as the difference between the energy of a single relaxed molecule and the energy of an unrelaxed molecule in the crystal structure geometry. Important information on the intermolecular interaction strength within the tetramer is obtained when one monomer is removed from the system. This energy (depicted as *E*_remove_ in Figures 11, 13, and 15) contains the interactions with neighboring molecules and also possible long-range interactions within the tetramer. This energy is determined as a difference between the interaction energies of a tetramer and trimer formed after removing the appropriate monomer.

**Figure 12:**
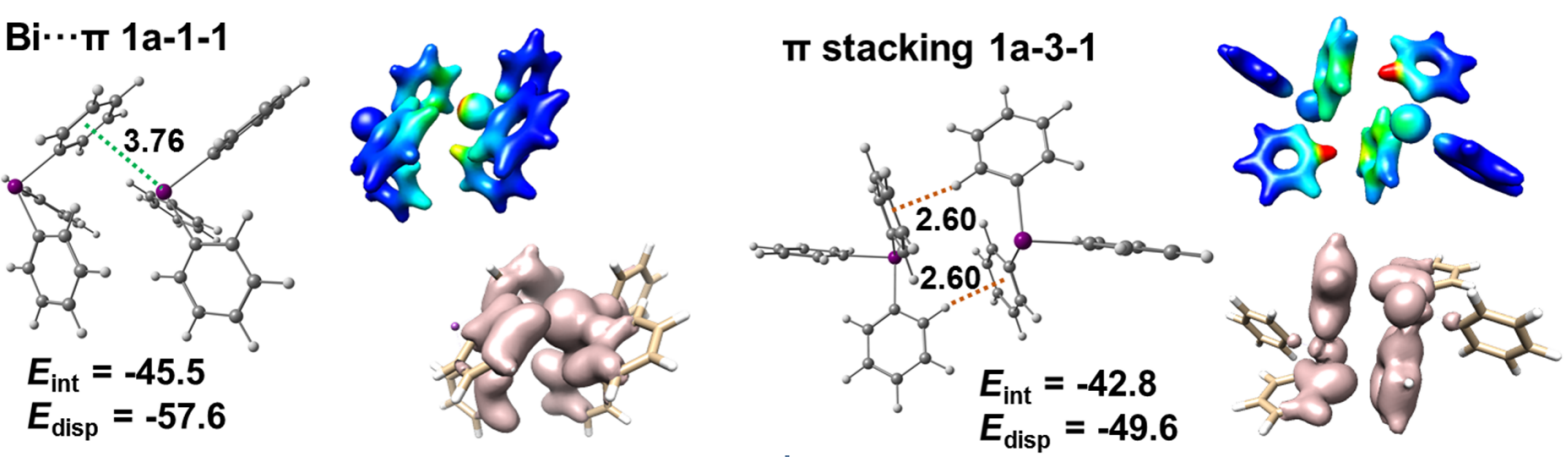
Detailed structures of selected dimers extracted from the crystal structure of polymorph **1a** and dispersion energy plots. *E*_int_ denotes the interaction energy of a given dimer and *E*_disp_ is the dispersion energy obtained from LED analysis computed at the DLPNO-CCSD(T)/cc-pVQZ (cc-pwCVQZ-PP for Bi, TightPNO settings) level of theory. The distances are given in Å and energies are in kJ mol^−1^.

**Figure 13:**
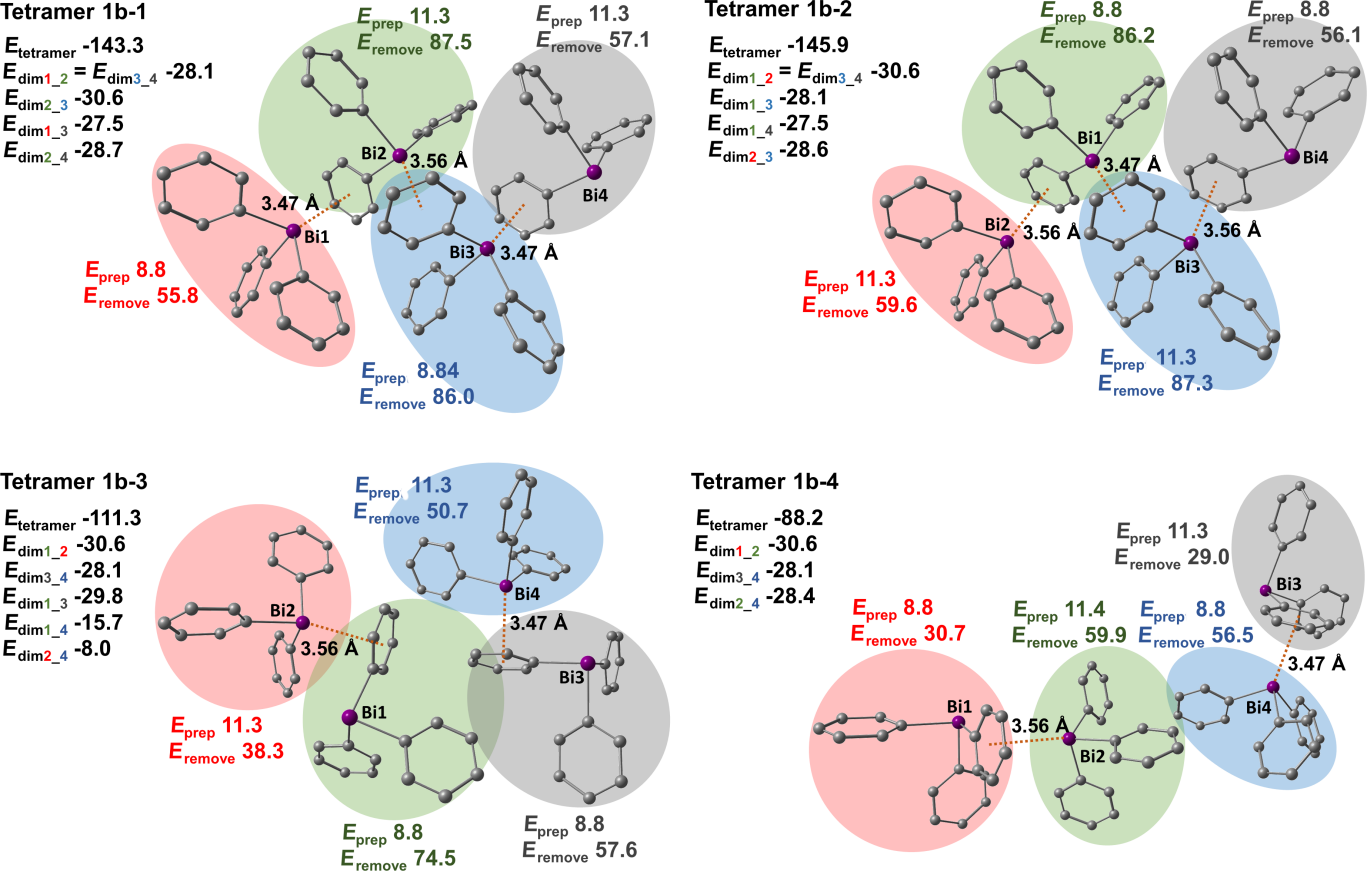
Structures of studied BiPh_3_ tetramers extracted from the crystal structure of polymorph **1b**. See Figure 11 for details.

**Figure 14:**
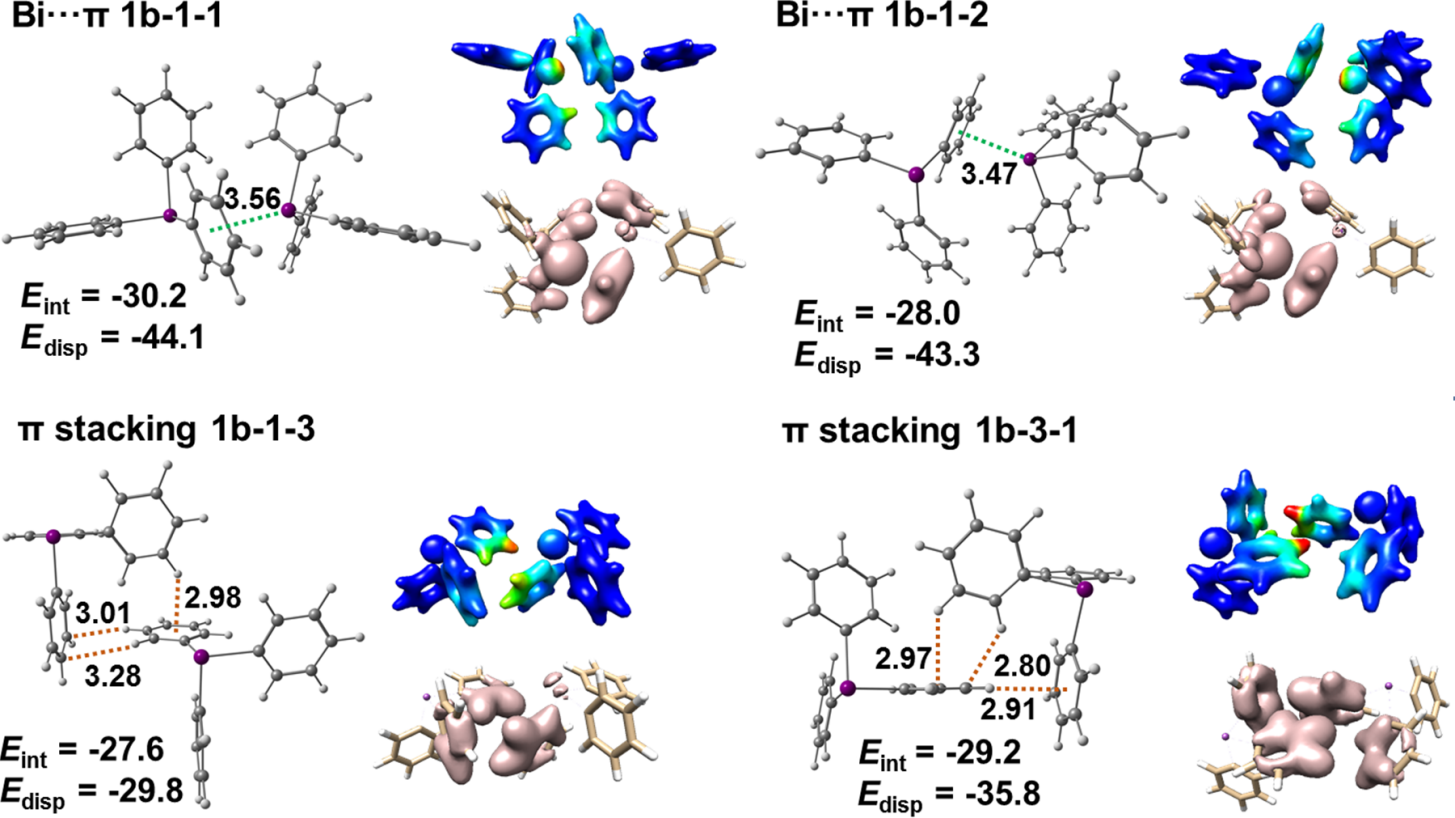
Detailed structures of selected dimers extracted from the crystal structure of polymorph **1b** and dispersion energy plots. See Figure 12 for details.

**Figure 15:**
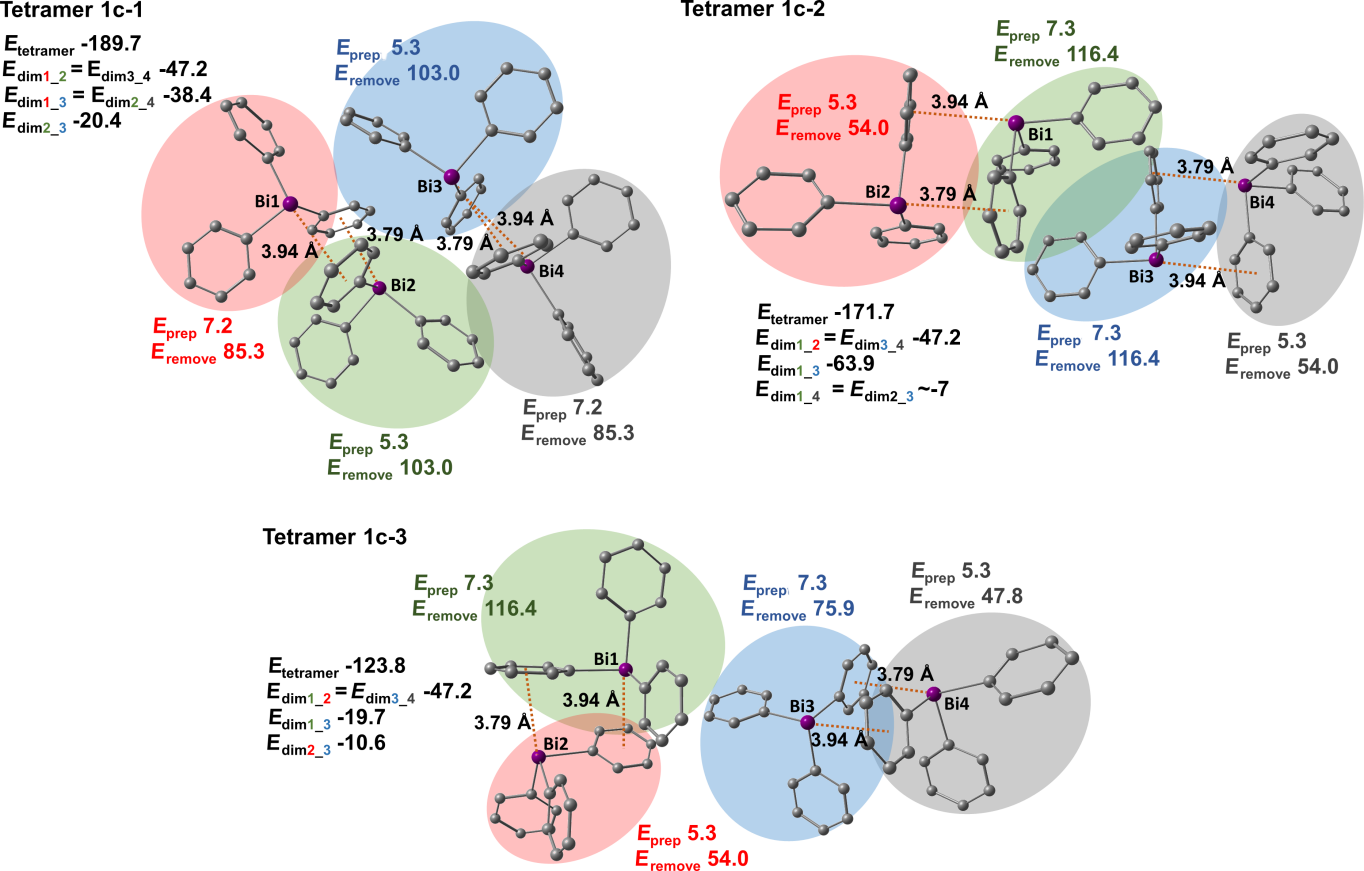
Structures of studied BiPh_3_ tetramers extracted from the crystal structure of polymorph **1c**. See Figure 11 for details.

**Figure 16:**
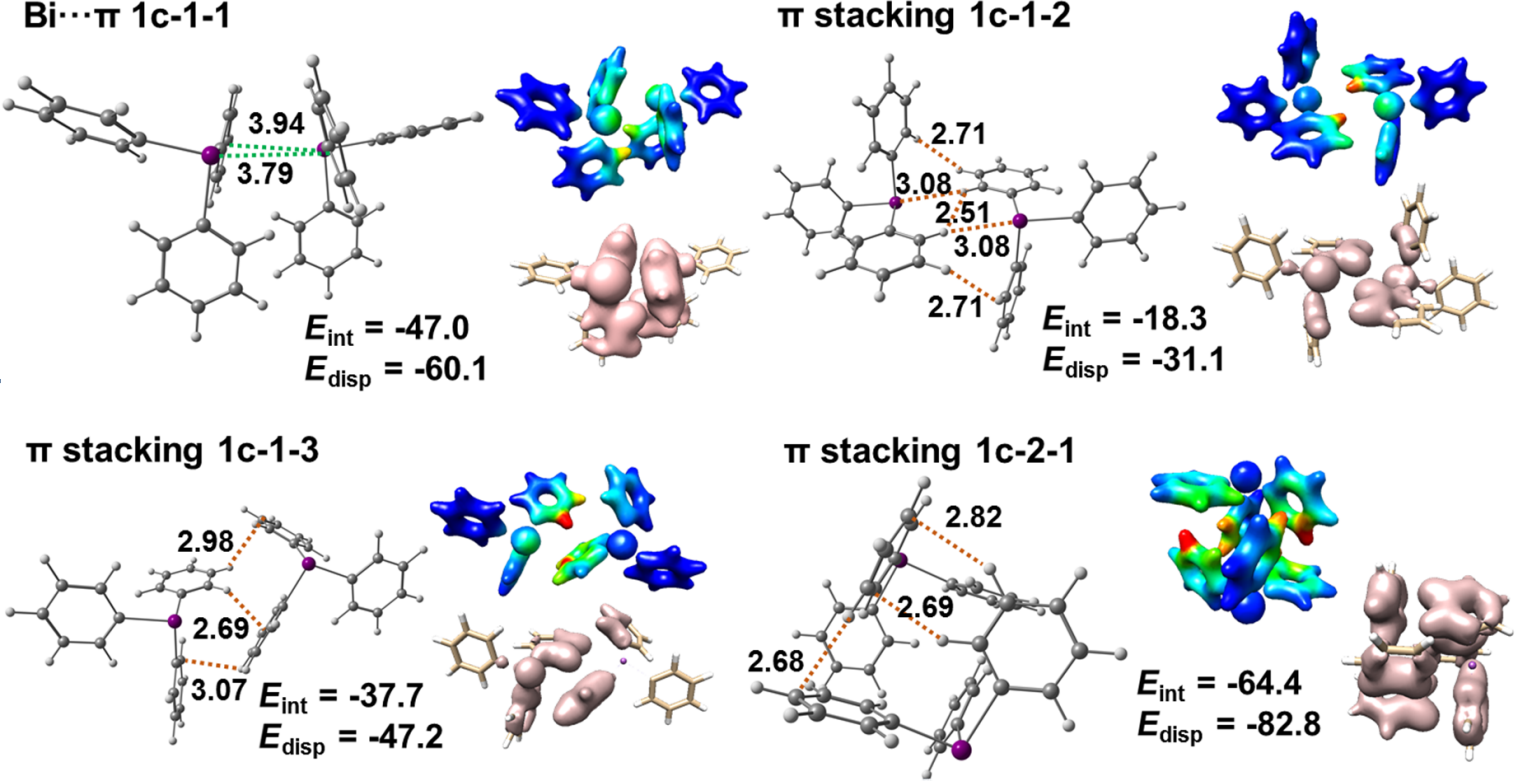
Detailed structures of selected dimers extracted from the crystal structure of the polymorph **1c** and dispersion energy plots. See Figure 12 for details.

The interaction energies of all tetramers and dimers were computed at the PBE-D3/def2-TZVP level of theory and are depicted in Figures 11, 13, and 15 as *E*_tetramer_ and *E*_dim_, respectively. A color code is introduced in Figures 11, 13, and 15 to facilitate the understanding of the construction of tetramers and dimers. A different color is ascribed to each monomer within the tetramer. The interaction energies were computed with reference to the sum of the energies of all unrelaxed monomers (crystal structure geometry) included in the tetramer or dimer. Interaction energies of tetrameric, trimeric, and dimeric structures can yield information about the additivity of intermolecular interactions. Additionally, interaction energies of all Bi···π arene type dimers and selected π-stacking dimers were computed at the DLPNO-CCSD(T)/cc-pVQZ (cc-pwCVQZ-PP for Bi) level of theory with TightPNO settings (see Figures 12, 14, and 16). Local energy decomposition analysis was performed in order to obtain the dispersion energy contributions to the interaction energies. The dispersion energies of the specific dimers were then visualized as DED plots and are shown in Figures 12, 14, and 16. The structures and interaction energies of all studied π-stacking dimers are given in Supporting Information File 1 (see Figures S12–S14 and Table S4 in Supporting Information File 1). Please note that the positions of the hydrogen atoms in the tetrameric and dimeric structures were optimized at the PBE-D3/def2-TZVP level of theory. Hence, the intermolecular distances involving C–H groups may vary from the crystallographic data given in the previous sections.

#### Polymorph **1a**

In case of polymorph **1a** three different tetramers were chosen that are shown in Figure 11. The simplest tetramer **1a-1** consists of linear chains of BiPh_3_ molecules belonging to one layer. It is built from three equivalent Bi···phenyl dimers with an interaction energy of −46 kJ mol^−1^ (computed at the PBE-D3/def2-TZVP level of theory, depicted in Figure 11 as *E*_dim3_2_). Tetramers **1a-2** and **1a-3** are constructed from two Bi···π arene dimers of two different layers of BiPh_3_ molecules. Within these two tetramers not only Bi···π arene interactions are present but also π-stacking contacts of monomers between two layers. In tetramer **1a-2** three C–H_Ph_···π stacking interactions can be found. Two equal interactions between monomers 2 (green; numbering of the monomers is the same as the numbering of the Bi atoms within a specific tetramer as depicted in Figure 11) and 3 (red), and monomers 1 (grey) and 4 (blue). There is also a C–H_Ph_···π type interaction between monomers 2 (green) and 4 (blue). All of these dimers have interaction energies of −29 kJ mol^−1^ (depicted in Figure 11 as *E*_dim2_3_ and E_dim2_4_) indicating that they are interacting fairly strongly. Similarly, in tetramer **1a-3** three π-stacking interactions between monomers can be found. Two of them (monomers 1 and 4, and 2 and 3) are equivalent and their interaction energy amounts to −42 kJ mol^−1^ indicating strong interactions between the layers. The next neighbor interaction between monomers 2 (green) and 4 (blue) in tetramer **1a-3** is much weaker and amounts to −15 kJ mol^−1^.

The distortion energies (*E*_prep_) of the monomers in polymorph **1a** are quite large and amount to almost 17 kJ mol^−1^. This indicates that, although the interactions between specific monomers to form dimeric structures by Bi···π arene are strong, the crystal packing effects are significant in this case.

Another factor that is useful in describing the energetics within the tetramers is the energy required to remove one of the monomers from the corresponding tetramer. This quantity is depicted in Figure 11 as *E*_remove_. By removing one of the molecules from the tetramer the interactions with this specific monomer are broken, which can involve contacts between neighboring molecules or long-range interactions. In tetramer **1a-1** the energy needed to remove one of the outer monomers (3 and 4) is almost equal to the interaction energy of one Bi···π arene dimer. This indicates that only one dimer breaks. To remove one of the inner monomers (2 or 1) an energy of 92 kJ mol^−1^ is required. This energy is very close to the sum of the interaction energies of two dimers. For example, *E*_remove_ of monomer 2 in tetramer **1a-1** is roughly the sum of the interaction energies of two Bi···π arene dimers (91.4 kJ mol^−1^). This simple example shows the additivity of the dimeric intermolecular interactions within the tetramer. The situation is more complicated for tetramers **1a-2** and **1a-3**. In general, the energies to remove one of the molecules from these tetramers are higher than for tetramer **1a-1** as they contain interactions between the chains and each of the monomers has more contacts on average within the tetramer. The energy needed to remove one of the monomers from tetramer **1a-2** or **1a-3** is roughly the sum of the interaction energies involving this monomer (deviating by at most 5 kJ mol^−1^). Another important aspect is that the interaction energy computed for tetramers **1a-1**–**1a-3** can be also expressed as a sum of energies of particular dimeric interactions present in the specific tetramer. For instance, the sum of the interaction energies of specific dimers in tetramer **1a-2** amounts to −179 kJ mol^−1^ which is higher by 4 kJ mol^−1^ than the interaction energy of the whole tetramer (−175.1 kJ mol^−1^). For tetramer **1a-3** this sum is −191.3 kJ mol^−1^ which is very similar to the computed value (−189.6 kJ mol^−1^). This shows that the interactions in the crystal structure of polymorph **1a** are pairwise neighbor interactions. Long-range interactions are probably mostly weak dipolar interactions that do not contribute significantly. The analysis of specific dimeric interactions in tetramers **1a** shows that not only Bi···π arene interactions are important structure building factors but also several C–H_Ph_···π arene contacts play a crucial role in structure formation. The strength of the π-stacking interactions depends on the number of contacts and distances between interacting molecules.

Figure 12 depicts structures of Bi···π arene dimers and one of the (strongest) C–H_Ph_···π arene dimers in polymorph **1a**. The C–H_Ph_···π arene dimer (depicted as **1a-3-1** in Figure 12) has two very short (2.6 Å) contacts between a C–H group and a phenyl ring. The interaction and dispersion energies given in Figure 12 were obtained at the DLPNO-CCSD(T)/cc-pVQZ (cc-pwCVQZ-PP for Bi and TightPNO settings) level of theory. Note that PBE-D3 and DLPNO-CCSD(T) give very similar results. Inspection of dispersion energies obtained from the LED analysis reveals that both types of dimers (Bi···π arene and π-stacking) are exclusively dispersive. Figure 12 depicts two complementary graphical interpretations of the dispersion energy density that can be plotted either as an isosurface (coral plots) or mapped on an isodensity surface (color gradient) of the electron density. Both plots display the regions with the highest contributions to the dispersion interaction present in the complex.

In summary, polymorph **1a** is formed by one dimensional chains consisting of strong Bi···π arene contacts with interaction energies of −46 kJ mol^−1^ (see tetramer **1a-1** in Figure 11). These 1D chains are bound strongly by the π-type interactions present between BiPh_3_ molecules belonging to different layers. The energies of such contacts range from −15 to −40 kJ mol^−1^. The distortion energies of monomers (17 kJ mol^−1^) suggest that crystal packing effects are large in polymorph **1a**.

#### Polymorph **1b**

In case of polymorph **1b** four tetrameric units were identified and are depicted in Figure 13. Tetramers **1b-1** and **1b-2** consist of zig-zag chains formed by Bi···π arene interactions between one layer of BiPh_3_ molecules. The difference between these tetramers is that tetramer **1b-1** contains two Bi···π arene contacts with a distance of 3.47 Å between the Bi atom and the phenyl ring centroid and one Bi···π arene contact with a distance of 3.56 Å. In tetramer **1b-2** there are two 3.56 Å Bi···π arene contacts and one 3.47 Å contact. Both types of Bi···π arene dimers have very similar interaction energies of −28 kJ mol^−1^ (*E*_dimer1_2_ of tetramer **1b-1**) and −31 kJ mol^−1^ (*E*_dimer2_3_ of tetramer **1b-1**). In tetramers **1b-1** and **1b-2** π-stacking interactions are also present. We only discuss the interactions in tetramer **1b-1** as they are the same as in tetramer **1b-2**. For example, the C–H_Ph_···π arene-type interactions between monomers 1 (red) and 3 (blue), and monomers 2 (green) and 4 (grey) are as strong as the Bi···π arene interactions in polymorph **1b** and amount to −28 kJ mol^−1^ and −29 kJ mol^−1^, respectively. Tetramers **1b-3** and **1b-4** on the other hand, exhibit interactions between neighboring zig-zag chains. In tetramer **1b-3** two Bi···π arene interactions and three other stacking interactions are present that vary in energy and contact area between the BiPh_3_ molecules. For example, the strongest C–H_Ph_···π arene dimer of tetramer **1b-3** with an interaction energy of −30 kJ mol^−1^ is formed between monomers 1 (green) and 3 (grey). The other two stacking interactions between monomers 1 (green) and 4 (blue), and monomers 2 (red) and 4 (blue) are much weaker due to the smaller contact area between the molecules. Their interaction energies amount to −16 kJ mol^−1^ and −8 kJ mol^−1^, respectively. In tetramer **1b-4** only one additional π-stacking interaction between molecules 2 (green) and 4 (blue) is present but is quite strong (*E*_dim2_4_ = −28 kJ mol^−1^).

The distortion energies of the monomers (*E*_prep_) are much lower than that for polymorph **1a** and range from 9 to 11 kJ mol^−1^. This implies that crystal packing effects are less pronounced in this polymorph.

Energies required to remove one of the monomers from the tetramer in case of polymorph **1b** also depend on the number of interactions and the energy value is roughly the sum of the energies of these interactions. For instance, one of the lowest energies needed to remove a molecule is found for monomer 2 (red) in tetramer **1b-3** that amounts to 38 kJ mol^−1^. This energy is simply the sum of the already discussed dimer energies (*E*_dim1_2_ and *E*_dim2_4_). Another example is when monomer 3 (blue) is removed from tetramer **1b-1** which requires an energy of 86 kJ mol^−1^. This energy is a sum of the dimer energies that are formed including this monomer (*E*_dim1_3_, *E*_dim2_3_, and *E*_dim3_4_). This again demonstrates the additivity of intermolecular interactions and that the intermolecular interactions in the bulk can be described as a sum of dimer interactions. Adding up energies of all the dimeric units of tetramer **1b-3** results in an energy of −112.2 kJ mol^−1^ which is roughly equal to the computed interaction energy of this tetramer (−111.3 kJ mol^−1^).

Figure 14 depicts Bi···π arene dimers and selected π-stacking dimers that are present in the structures of tetramers **1b-1**–**1b-4** and their dispersion energy density plots. It is concluded that the dispersion energies are a few kJ mol^−1^ higher for Bi···π arene dimers than for π-stacking dimers but in general all of these interactions are purely dispersive. Note that typically the dispersion contribution is larger than the overall interaction energy as it compensates the monomer preparation.

Summarizing, polymorph **1b** is built from zig-zag chains of BiPh_3_ molecules consisting of Bi···π arene contacts with interaction energies of about −30 kJ mol^−1^. The preparation energy is notably smaller than in polymorph **1a**. The contacts between zig-zag chains are of π-stacking type and their interaction energies amount also to about −30 kJ mol^−1^.

#### Polymorph **1c**

For polymorph **1c** we identified three tetramers that are depicted in Figure 15. Each of them contains two Bi···π dimers that consist of two Bi···π arene contacts. The interaction energy of these dimers is high and amounts to −47 kJ mol^−1^. Tetramer **1c-1** represents Bi···π arene dimers belonging to the same layer. There are two identical C–H_Ph_···π arene dimers in tetramer **1c-1** with an interaction energy of −38 kJ mol^−1^. These π-stacking dimers are formed by monomers 2 (green) and 4 (grey), and monomers 1 (red) and 3 (blue). The detailed structure of this dimer is shown in Figure 16 (depicted as **1c-1-1**). Monomers 2 (green) and 3 (blue) also interact to form a dimer that is based on CH···CH and CH···phenyl interactions (see Figure 16, **1c-1-2**). The interaction energy of this dimeric unit is moderate and amounts to −20 kJ mol^−1^. Tetramers **1c-2** and **1c-3** include the Bi···π arene dimers from two different molecular layers. In tetramer **1c-2** one very strongly bound π-stacking dimer is formed between monomers 1 (green) and 3 (blue). Its interaction energy amounts to −64 kJ mol^−1^ and is the highest among all studied dimers, including Bi···π arene dimers. Its structure resembles the structure of a cube with multiple short C–H_Ph_···π arene contacts (see Figure 16, dimer **1c-2-1**). In case of tetramer **1c-3** the π-stacking interaction between the molecules are weaker than in the other two tetramers. The interaction between monomers 1 (green) and 3 (blue) is of moderate strength and the interaction energy is −20 kJ mol^−1^. The second π-stacking interaction present in the tetramer involves monomers 2 (red) and 3 (blue) and is a rather weak interaction with −11 kJ mol^−1^.

The distortion energies (*E*_prep_) of the monomers of polymorph **1c** are very low and range from 5 to 7 kJ mol^−1^. This means that in this case packing effects are not very strong compared to the other two polymorphs of BiPh_3_.

In case of polymorph **1c** energies required to remove one molecule from the tetramer are significantly higher and are roughly the sum of the interactions that involve a specific monomer. Note that the sum of interaction energies of dimers in tetramer **1c-2** amounts to −158.3 kJ mol^−1^ which is much smaller than the interaction energy of the tetramer (*E*_tetramer_ = −171.7 kJ mol^−1^). Most probably there are two possible interactions between slightly remote monomers 1 (green) and 4 (grey), and 2 (red) and 3 (blue) each accounting for about −7 kJ mol^−1^. In case of the other two tetramers of polymorph **1c** all interaction energies of dimers add up to roughly the interaction energy of the tetramer.

Figure 16 depicts the most important dimers found in the structure of polymorph **1c**, for which the interaction and dispersion energies are given. The dispersion energy plots show the spatial distribution of the dispersion interaction within each dimer. An especially high dispersion energy contribution is observed for dimer **1c-2-1** (−83 kJ mol^−1^). By looking at the distribution of the dispersion energy (dispersion energy density plots) for this dimer it is noticed that almost the entire monomers contribute to the overall dispersion from π···π interactions.

In case of polymorph **1c** the results of the quantification of the interaction energies reveal that this structure is actually not dominated by Bi···π arene interaction, but rather consists of dimers connected by strong π···π interaction (Figure 16, **1c-2-1** with −64 kJ mol^−1^) which are connected by two weaker Bi···π arene contacts (Figure 16, **1c-1-1**, −47 kJ mol^−1^).

**Figure 17:**
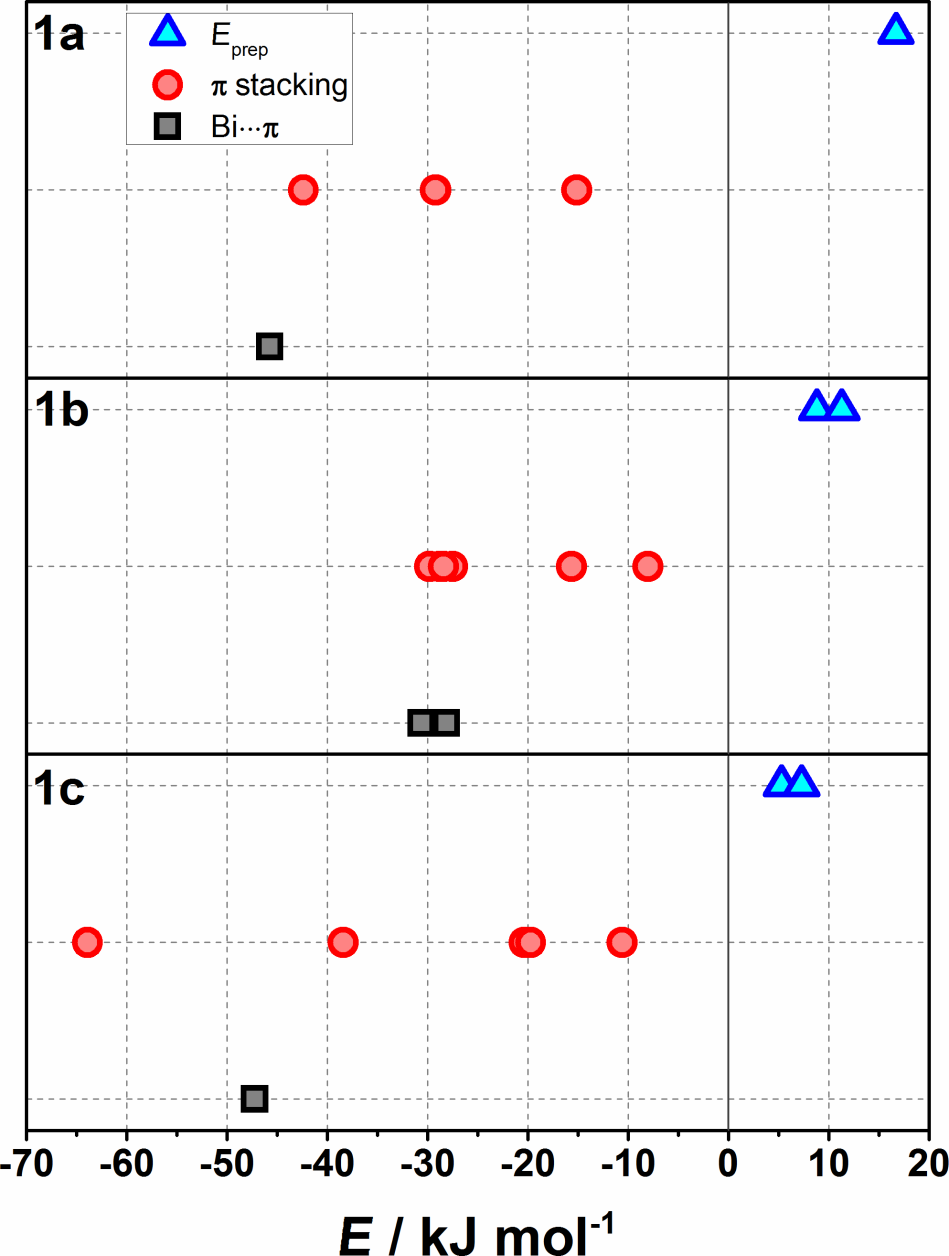
Distortion energies of monomers (*E*_prep_, blue triangles) and interaction energies of Bi···π and π-stacking dimers (grey squares and red dots, accordingly) computed at the PBE-D3/def2-TZVP level of theory for polymorphs **1a**, **1b**, and **1c** of compound **1**. Note that every point denotes a distortion energy or dimer interaction energy obtained for the given polymorph.

Figure 17 shows the comparison of the distortion and the interaction energies of Bi···π arene and π stacking dimers. As for BiPh_3_, Bi···π arene and π···π interactions are of comparable magnitude and the existence of the different polymorphs can be explained by a balance of two competing interactions. While in case of polymorph **1a** the Bi···π arene interaction dominates as a structure building factor, for polymorph **1b** Bi···π arene and π···π interactions are in the same energy range. In case of polymorph **1c** the π-stacking interaction dominates. Figure 17 also demonstrates the differences between distortion energies of monomers (*E*_prep_) as found for a specific polymorph and shows how packing effects decrease from polymorph **1a** to **1c**.

## Conclusion

Herein, we have shown that the dispersion type Bi···π arene interactions provide an important contribution to the structure formation of arylbismuth compounds. In the absence of stronger donors such as -OR and -Cl, the dispersion type bismuth···π arene interaction is supplemented by other weak interactions such as π···π or C–H_Ph_···π. Each Bi···π arene contact with bismuth as a strong dispersion energy donor (DED) provides a higher interaction energy than a single C–H_Ph_···π contact, but most often several of the latter compete with the single Bi···π arene contact. In case of multiple C–H_Ph_···π contacts these can become dominating. As a result, triorganobismuth compounds show a diversity of polymorphs.

In compounds with bulky ligands the formation of Bi···π arene contacts is hindered and multiple C–H_Ph_···π contacts dominate. In the presence of strong structure directing donor–acceptor bonds the role of bismuth as DED can usually be neglected. However, compounds of the type Ar_2_BiX (Ar = C_6_H_3_-*t*-Bu_2_-3,5, X = halide) show some special features. In these compounds the formation of a one dimensional ribbon as a result of Bi···X···Bi coordination is typical, which is supplemented by Bi···π arene interactions between two neighboring bismuth atoms in the chain. Thus, a sort of X, π-pincer system is obtained. In order to strengthen the Bi···π arene interaction and to induce directionality in structure formation it is important to introduce electron-withdrawing substituents. Otherwise, a subtle interplay between Bi···π arene and the dispersion type forces must be considered.

Analysis of the Bi···π arene interaction in the BiPh_3_–benzene complex shows that it is of moderate strength (−17 kJ mol^−1^). Comparing the BiPh_3_–benzene complex with other BiR_3_-benzene systems (with R = Me, OMe, Cl) exhibits that the nature of this complex is mainly dispersive with small addition of donor–acceptor character which brings it closer to the BiMe_3_ rather than to Bi(OMe)_3_ as a dispersion energy donor. The weak donor–acceptor character of BiPh_3_ causes that the Bi···π arene interactions compete with π···π and C–H_Ph_···π interactions. Inspection of the intermolecular interactions in polymorphs **1a**, **1b**, and **1c** of BiPh_3_ (**1**) confirms that Bi···π arene interactions are very important building blocks of the bulk. These are rather strong with interaction energies in the range from −28 kJ mol^−1^ to −47 kJ mol^−1^ and are purely dispersive. These energies are much higher than the interaction energy obtained for the model BiPh_3_···benzene system. An analysis of selected tetramer units reveals that also π-stacking interactions and contacts between layers of BiPh_3_ molecules are crucial in the formation of the crystal structures. The interaction energies of the π-stacking dimers are as high as interaction energies of Bi···π arene complexes or even larger (−64 kJ mol^−1^). The energy of such dimers depends strongly on the distance and the contact area between two monomers. Both types of dimers are exclusively dispersive as shown by LED analysis performed at the DLPNO-CCSD(T) level of theory. Analysis of tetrameric units also reveals that the interaction energy of tetramers is additive and can be described as a sum of interaction energies of particular dimers.

In the polymorphs of compound **1** the energetics of interactions is balanced between Bi···π arene and π···π interactions that are of comparable strength. In case of polymorph **1a** the Bi···π arene interaction dominates, in case of polymorph **1b** the Bi···π arene and the π···π interactions are of similar magnitude. For polymorph **1c**, π···π interactions dominate the intermolecular interactions.

Overall, the compounds and structures discussed in this work demonstrate that a broad range of intermolecular interaction motifs are accessible by tuning the donor–acceptor properties of bismuth as a dispersion energy donor. Using electronic structure theory, these interactions can be quantified and studied in detail.

## Experimental

### Crystallographic studies

Crystal data, data collection and refinement parameters for Ph_3_Bi (polymorphs **1a**, **1b**, **1c**, **1d**), **1b**, **2a**, **2b** and **3, 4**, **5**·2CH_2_Cl_2_ are given in Table S1, Table S2 and Table S3 (in Supporting Information File 1), respectively. All data for the new structures were collected with an Oxford Gemini S diffractometer at 123 K (**1b**), 120 K (**2a**, **3**), 115 K (**4**, **5**·2CH_2_Cl_2_) and 100 K (**2b**) using Cu Kα radiation (λ = 1.54184 Å) for **2a** and Mo Kα radiation (λ = 0.71073 Å) for **1b**, **2b**, **3**, **4**, **5**·2CH_2_Cl_2_. The structures were solved by direct methods using SHELXS-2013 [68–69] and refined by full-matrix least-square procedures on *F*^2^ using SHELXL-2014 [68,70] and SHELXL-2016/6 [71]. All non-hydrogen atoms were refined anisotropically. All hydrogen atoms were geometrically placed and refined isotropically in riding modes using default parameters. The drawings were created with the Diamond program [72]. The identity of Ph_3_Bi (polymorphs **1a**, **1b**), **2a**, **2b**, and **3** was confirmed by PXRD analyses. The simulated diffraction patterns of three polymorphs of Ph_3_Bi (**1a**, **1b**, **1c**) are illustrated in Figure S6 (see Supporting Information File 1). The diffraction patterns of the measured diffractograms are in good agreement with those simulated from the single crystal X-ray crystallographic data (see Supporting Information File 1, Figures S7–S11). The crystal structure of **2b** shows one disordered aryl ring over the whole aryl ligand with an occupancy ratio of 0.689:0.311 (69:31%). CCDC 1828668 (**1b**), 1824685 (**2a**), 1824684 (**2b**), 1824683 (**3**), 1824221 (**4**), 1824222 (**5**).

## Supporting Information

Synthesis of compounds **1**–**5**. Molecular structures of **2a**, **2b**, **4**, and **5** (Figures S1–S4). Temperature dependent PXRD of Ph_3_Bi (**1a**, Figure S5), PXRD pattern of the three Ph_3_Bi polymorphs (Figure S6), PXRD pattern of **1a**, **1b**, **2a**, **2b**, and **3** (Figures S7–S11). Crystallographic data and structure refinement details for (**1a**), [42] (**1b**), [45] (**1c**) [44] and (**1d**) [39], **1b**, **2a**, **2b** and **3**–**5**, respectively (Tables S1, S2, and S3). Computational details. Structures of π-stacking dimers found for polymorph **1a**, **1b** and **1c** of Ph_3_Bi (Figures S12–S14). Interaction energies (with respect to BiPh_3_ in crystal geometry) and total energies (with respect to fully relaxed BiPh_3_) in kJ mol^−1^ of π-stacking dimers (Table S4). Cartesian coordinates.

File 1 Additional material.
