# Supplementary material for: Evaluation of dispersion type metal···π arene interaction in arylbismuth compounds – an experimental and theoretical study
Source: Beilstein J Org Chem. 2018 Aug 15;14:2125–45. doi: 10.3762/bjoc.14.187 (PMC6122404; doi:10.3762/bjoc.14.187)
Supplement: File 1 — Additional material. [file Beilstein_J_Org_Chem-14-2125-s001.pdf]

Supporting Information

for

Evaluation of dispersion type metal···π arene interaction in arylbismuth compounds – an experimental and theoretical study

Ana-Maria Preda[1], Małgorzata Krasowska[2], Lydia Wrobel[1], Philipp Kitschke[1], Phil C. Andrews[3], Jonathan G. MacLellan[3], Lutz Mertens[1], Marcus Korb[4], Tobias Rüffer[4], Heinrich Lang[4], Alexander A. Auer*[2] and Michael Mehring*[1]

Address: [1]Technische Universität Chemnitz, Fakultät für Naturwissenschaften, Institut für Chemie, Professur Koordinationschemie, 09107 Chemnitz, Germany, [2]Max-Planck-Institut für Kohlenforschung, Kaiser-Wilhelm-Platz 1, 45470 Mülheim an der Ruhr, Germany, [3]School of Chemistry, Monash University, Clayton, Melbourne, VIC 3800, Australia and [4]Technische Universität Chemnitz, Fakultät für Naturwissenschaften, Institut für Chemie, Professur Anorganische Chemie, 09107 Chemnitz, Germany

Email: Alexander A. Auer* - alexander.auer@kofo.mpg.de; Michael Mehring* - michael.mehring@chemie.tu-chemnitz.de

* Corresponding author

Additional material

Table of contents:

Experimental - General procedure S2
Computational details S3
Synthesis of compounds **1–5** S3

Figure S1 Molecular structure of $(C_6H_4\text{-}CH{=}CH_2\text{-}4)_3Bi$ (**2a**) S7
Figure S2 Molecular structure of $(C_6H_4\text{-}CH{=}CH_2\text{-}4)_3Bi$ (**2b**) S7
Figure S3 Molecular structure of $(C_6H_3\text{-}t\text{-}Bu_2\text{-}3,5)_3Bi$ (**4**) S8
Figure S4 Molecular structure of $(C_6H_3\text{-}t\text{-}Bu_2\text{-}3,5)_2BiCl$ (**5**) S8
Figure S5 Temperature dependent PXRD of Ph_3Bi (**1a**) S9
Figure S6 The PXRD pattern of the Ph_3Bi polymorphs **1a**, **1b**, **1c** S9
Figure S7 Powder X-ray diffraction pattern of Ph_3Bi polymorph **1a** S10
Figure S8 Powder X-ray diffraction pattern of Ph_3Bi polymorph **1b** S10
Figure S9 Powder X-ray diffraction pattern of polymorph $(C_6H_4\text{-}CH{=}CH_2\text{-}4)_3Bi$ (**2a**) S11
Figure S10 Powder X-ray diffraction pattern of polymorph $(C_6H_4\text{-}CH{=}CH_2\text{-}4)_3Bi$ (**2b**) S11
Figure S11 Powder X-ray diffraction pattern of $(C_6H_4\text{-}OMe\text{-}4)_3Bi$ (**3**) S12
Table S1 Crystallographic data and structure refinement details for **1a**, **1b**, **1c**, **1d** S12
Table S2 Crystallographic data and structure refinement details for **1b**, **2a**, **2b** S13
Table S3 Crystallographic data and structure refinement details for **3–5** S14
Figure S12 Structures of π stacking dimers found for polymorph **1a** of $BiPh_3$ S15
Figure S13 Structures of π stacking dimers found for polymorph **1b** of $BiPh_3$ S16
Figure S14 Structures of π stacking dimers found for polymorph **1c** of $BiPh_3$ S17
Table S4. Interaction energies and total energies (kJ mol^{-1}) of π stacking dimers of Ph_3Bi S18
Cartesian coordinates S18
References S38

Experimental

General procedure

All procedures were carried out under anhydrous nitrogen or argon using standard Schlenk techniques. Solvents were freshly distilled over appropriate drying reagents immediately prior to use. Reagents such as bromobenzene, 4-bromostyrene, 4-bromoanisole and 1-bromo-3,5-di-*tert*-butylbenzene were purchased from commercial suppliers and used as received. Ph_3Bi was prepared, referring to a modified literature procedure [1,2] from the Grignard reaction of PhMgBr (synthesized from bromobenzene and Mg turnings) with sublimed and anhydrous $BiCl_3$ in dried THF. The precipitate obtained after pouring the reaction solution onto ice was recrystallized from EtOH. ATR-FTIR spectra were recorded with a Bio Rad FTS-165 spectrometer with a golden gate sample adapter. ^1H (500.30 MHz) and $^{13}C\{^1H\}$ NMR (125.81 MHz) spectra were recorded with a Bruker spectrometer (Avance III 500) at ambient temperature. The ^1H and $^{13}C\{^1H\}$ NMR chemical shifts are reported in δ units (ppm) relative to the residual peak of the deuterated solvent (ref. $CDCl_3$: ^1H, 7.26 ppm, ^{13}C, 77.16 ppm. The NMR spectra were processed using the MestReNova software [3]. The CHN-analyses were carried out with a CHN-Analyser Type FlashEA 1112 (Thermo Fisher Scientific). The melting points of compounds were measured with Melting Point B-540 apparatus (Büchi). PXRD studies were carried out with a STOE-Stadi P device using Cu Kα (λ = 1.54184 Å) radiation.

Computational details

All calculations were performed using the development version of Orca 4.0.1 [4,5]. Geometries were optimized using the PBE [6, 7] density functional in conjunction with the def2-TZVP [8] basis set and the default effective core potential (ECP) for Bi. Fine integration grids (grid 4) were applied. The dispersion correction by Grimme [9] with Becke-Johnson damping (D3BJ) [10] as well as the resolution of identity approximation [11] with appropriate auxiliary basis set were used in all DFT calculations. Stationary points were confirmed by subsequent normal mode analysis. The energies were refined by employing the DLPNO-CCSD(T) method [12-18]. The cc-pVQZ [19] basis set was used for lighter atoms and the cc-pwCVQZ-PP [20] basis set with a SK-MCDHF-RSC effective core potential was employed for Bi. TightPNO [21,22] settings were used in all DLPNO-CCSD(T) computations. Additionally, local energy decomposition (LED) [23,24] analysis was performed in order to obtain dispersion energy contributions to the total interaction energy at the DLPNO-CCSD(T) level of theory. The dispersion interaction obtained from LED can be visualized using a mapping scheme based on the pair energies contributions [25,26]. The dispersion energy contribution can be displayed as an isosurface or can be mapped onto the isosurface of the electron density. The dispersion energy was visualized using the Chimera program [27]. The Chemcraft program was used to display molecular geometries [28]. The geometries of dimers, trimers, and tetramers were extracted from the crystal structures of $BiPh_3$ polymorphs and only the positions of hydrogen atoms were relaxed at the PBE/def2-TZVP as described above.

*Synthesis of Ph_3Bi (**1b**) – triphenylbismuthine* [1, 2]

A solution of 7.5 mL bromobenzene (11.260 g, 78.20 mmol, 3.3 equiv) in THF (50-55 mL) was added dropwise under stirring, to magnesium turnings (1.900 g, 78.20 mmol, 3.3 equiv) until the reaction started. The dropping rate was adjusted with any ice cooling so that the reaction mixture boils gently. After completion of the addition, the mixture was stirred at ambient temperature for 15–20 min and at 60–70 °C for additional 30 min. The Grignard solution was added dropwise at 0 °C to a solution of $BiCl_3$ (7.540 g, 23.90 mmol, 1 equiv) in THF (120 mL). After complete addition, the reaction mixture was warmed slowly to ambient temperature, stirred overnight and then poured onto about 700 mL of ice. When the ice had melted, the precipitate was filtered off, dried under ambient conditions and then suspended in 600 mL of EtOH. The suspension was heated to reflux and filtered hot. After concentration of the filtrate by heating or evaporation Ph_3Bi crystallized as colorless long needles, which were filtered and dried under ambient conditions. From the filtrate of the crystals, further product was obtained. Yield: 8.580 g (82% based on $BiCl_3$). M.p. = 78 °C (lit. [1] 77–78 °C).

Elemental analysis calcd. (%) for $C_{18}H_{15}Bi$ (440.29 g mol^{-1}): C, 49.10; H, 3.43. Found: C, 49.22; H, 3.33. ATR FTIR (cm^{-1}): $\tilde{\nu}$ = 3057 (m), 3045 (m), 3037 (m), 3018 (w), 3003 (w), 2976 (w), 2941 (vw), 2891 (vw), 2115 (vw), 2073 (vw), 2030 (vw), 1980 (vw), 1961 (w), 1945 (w), 1876 (w, br), 1864 (sh), 1829 (sh), 1814 (w, br), 1764 (vw, br), 1706 (vw), 1656 (vw), 1640 (vw), 1629 (vw), 1582 (vw), 1567 (s), 1474 (s), 1451 (vw), 1424 (vs), 1374 (w), 1328 (m), 1301 (m), 1262 (w), 1181 (m), 1158 (m), 1096 (vw), 1058 (s), 1015 (s), 996 (s), 965 (w), 899 (w), 884 (vw), 853 (w), 807 (vw, br), 718 (vs), 691 (vs, br), 645 (m), 618 (m), 560 (sh), 548 (w, br), 529 (sh), 490 (vw), 448 (vs), 432 (s). ^{1}H-NMR (500 MHz, $CDCl_3$): δ (ppm) 7.33 (tt, 3H, H_{para}, $^3J_{H\text{-}H}$ = 7.3, $^4J_{H\text{-}H}$ = 1.3 Hz, C_6H_5), 7.40 (t, 6H, H_{meta}, $^3J_{H\text{-}H}$ = 7.3 Hz, C_6H_5), 7.76 (dd, 6H, H_{ortho}, $^3J_{H\text{-}H}$ = 7.9, $^4J_{H\text{-}H}$ = 1.3 Hz, C_6H_5). ^{13}C{^{1}H}-NMR (125 MHz, $CDCl_3$): δ (ppm) 127.9 (s, C_{para}), 130.6 (s, C_{meta}), 137.7 (s, C_{ortho}), 155.3 (s, C_{ipso}).

*Synthesis of (C_6H_4-CH=CH_2-4)$_3$Bi (**2**) - tris(4-vinylphenyl)bismuthine*

A solution of 6.2 mL of 4-bromostyrene (8.670 g, 47.37 mmol, 3 equiv) in THF (35 mL) was added to magnesium turnings (1.270 g, 52.12 mmol, 3.3 equiv) and catalytic amounts (≤0.1 mg) of iodine in THF (15 mL) at 0 °C. After complete addition, the mixture was stirred for a further hour at 0 °C and then at ambient temperature until the magnesium was consumed. The precipitated solid was solubilized by addition of THF (20 mL). The Grignard solution was added dropwise to $BiCl_3$ (5.000 g, 15.79 mmol, 1 equiv) in THF (70 mL) at −20 °C. After warming to ambient temperature, the reaction mixture was stirred overnight, then poured onto about 500 mL of ice and stirred until the ice had melted. The resulting precipitate was filtered off, dried under ambient conditions and suspended in 500 ml of iPrOH. The suspension was heated to boiling and filtered hot. Concentration of the filtrate by heating or evaporation yielded **2** as a mixture of colorless needles and yellow block-shaped crystals, which were filtered off and dried under ambient conditions. From the filtrate of the crystals further product was obtained. Both crystal types of **2** were suitable for single crystal X-ray diffraction analysis. Yield: 6.890 g (84% based on $BiCl_3$). M.p. = 74 °C. Elemental analysis calcd. (%) for $C_{24}H_{21}Bi$ (518.40 g mol^{-1}): C, 55.60; H, 4.08. Found: C, 55.87; H, 4.09. ATR FTIR (cm^{-1}): $\tilde{\nu}$ = 3083 (w), 3056 (m), 3044 (m), 3002 (m), 2983 (m), 2952 (w), 2111 (vw), 2062 (vw), 1956 (vw), 1912 (w), 1896 (vw), 1831 (sh), 1814 (w, br), 1775 (vw), 1671 (vw), 1655 (vw), 1624 (s), 1582 (m), 1572 (vw), 1545 (m), 1536 (vw), 1486 (s), 1449 (vw), 1418 (m), 1385 (vs), 1343 (vw), 1318 (w), 1293 (m), 1264 (vw), 1204 (vw), 1183 (m), 1107 (m), 1055 (m), 1021 (s), 1007 (s), 984 (vs), 947 (w), 905 (vs), 845 (m), 822 (vs), 778 (s), 727 (vs), 633 (m), 575 (s), 475 (m), 446 (vs). ^{1}H-NMR (500 MHz, $CDCl_3$): δ (ppm) 5.25 (d, 3H, $^3J_{H\text{-}H}$ = 10.6 Hz, =$CH_{cis}H_{trans}$), 5.77 (d, 3H, $^3J_{H\text{-}H}$ = 17.6 Hz, =$CH_{cis}H_{trans}$), 6.70 (dd, 3H, $^3J_{H\text{-}H}$ = 17.5, 10.6 Hz, –C*H*=), 7.43 (d, 6H, H_3, $^3J_{H\text{-}H}$ = 7.4 Hz, C_6H_4), 7.72 (d, 6H, H_2, $^3J_{H\text{-}H}$ = 7.5 Hz, C_6H_4).

$^{13}C\{^1H\}$-NMR (125 MHz, $CDCl_3$): δ (ppm) 114.1 (s, $=CH_2$), 128.4 (s, C_3), 137.0 (s, C_4), 137.2 (s, –*C*H=), 137.9 (s, C_2), 155.0 (s, C_1).

Synthesis of $(C_6H_4\text{-}OMe\text{-}4)_3Bi$ *(**3**) - tris(4-methoxyphenyl)bismuthine*

A solution of 4.7 mL (11.72 mmol, 2.5 M in *n*-hexane, 3.17 equiv) of *n*-BuLi was added dropwise to a solution of 1.46 mL of 4-bromoanisole (2.180 g, 11.65 mmol, 3.15 equiv) in THF (35 mL) at −78 °C. Afterwards, the mixture was stirred for 15–30 min and then a solution of $BiCl_3$ (1.170 g, 3.70 mmol, 1 equiv) in THF (15 mL) was added dropwise at −78 °C. The reaction mixture was stirred for 1 h at −78 °C, then slowly warmed to ambient temperature and further stirred overnight. After removal of the solvent in vacuum, the residue was taken up in 60 mL of $CHCl_3$, filtered off and the filtrate was concentrated until a precipitate formed. The latter was redissolved by heating. On cooling, colorless block-shaped crystals of **3** were obtained, which were dried in vacuum after decanting off the mother liquor and washing with $CHCl_3$ (2 mL). Concentration of the mother solution in vacuum provided further product. Yield: 1.630 g (83% based on $BiCl_3$). M.p. = 193 °C. Elemental analysis calcd. (%) for $C_{21}H_{21}BiO_3$ (530.37 g mol^{-1}): C, 47.56; H, 3.99. Found: C, 46.99; H, 3.89. ATR FTIR (cm^{-1}): $\tilde{\nu}$ = 3081 (vw), 3056 (w), 3039 (w), 3010 (m), 2967 (m), 2933 (m, br), 2909 (sh), 2888 (sh), 2863 (vw), 2836 (m), 2801 (vw), 2109 (vw), 2078 (vw), 2060 (vw), 2041 (w), 2024 (vw), 2010 (vw), 1981 (vw), 1964 (vw), 1900 (w), 1875 (vw), 1856 (vw), 1814 (vw), 1794 (vw), 1787 (vw), 1754 (vw), 1702 (vw), 1665 (vw), 1655 (vw), 1638 (w), 1603 (vw), 1574 (vs), 1561 (s), 1528 (w), 1486 (vs), 1459 (s), 1451 (s), 1435 (vs), 1391 (s), 1364 (w), 1350 (vw), 1339 (w), 1306 (m), 1277 (vs), 1231 (vs), 1175 (vs), 1115 (m), 1100 (s), 1055 (vs), 1040 (s), 1024 (vs), 999 (s), 967 (m), 940 (m), 918 (sh), 901 (sh), 876 (w), 837 (s), 830 (vs), 816 (vs), 783 (vs), 708 (m), 670 (vw), 627 (m), 577 (s), 540 (w), 517 (vs), 467 (s). ^1H-NMR (500 MHz, $CDCl_3$): δ (ppm) 3.80 (s, 9H, OCH_3), 6.92 (d, 6H, H_3, $^3J_{H\text{-}H}$ = 8.5 Hz, C_6H_4), 7.62 (d, 6H, H_2, $^3J_{H\text{-}H}$ = 8.5 Hz, C_6H_4). $^{13}C\{^1H\}$-NMR (125 MHz, $CDCl_3$): δ (ppm) 55.2 (s, OCH_3), 116.5 (s, C_3), 138.9 (s, C_2), 145.3 (s, C_1), 159.5 (s, C_4).

Synthesis of $(C_6H_3\text{-}t\text{-}Bu_2\text{-}3,5)_3Bi$ *(**4**) - tris(3,5-di-tert-butylphenyl)bismuthine*

A solution of 1-bromo-3,5-di-tert-butylbenzene (3.380 g, 12.55 mmol, 3 equiv) in THF (25 mL) was added dropwise under stirring to magnesium turnings (0.366 g, 15.05 mmol, 3.2 equiv), activated with iodine (≤0.1 mg). The reaction was stirred for 4 h under reflux, then cooled to ambient temperature and the unreacted Mg was filtered off. The Grignard solution was added dropwise to a solution of $BiCl_3$ (1.312 g, 4.16 mmol, 1 equiv) in THF (20 mL) at 0 °C, and the reaction mixture was stirred overnight at ambient temperature. The solvent was removed under vacuum and the green residue was extracted with *n*-pentane (3 × 20 mL).

After the removal of the solvent, **4** was isolated a colorless solid. Yield: 2.362 g (73% based on $BiCl_3$). Single crystals suitable for X-ray analyses were grown from CH_2Cl_2 solution. M.p. = 144-146 °C. Elemental analysis calcd. (%) for $C_{42}H_{63}Bi$ (776.93 g mol^{-1}): C, 64.93; H, 8.17. Found: C, 64.35; H, 8.14. ATR FTIR (cm^{-1}): $\tilde{\nu}$ = 3044 (w), 2961 (vs), 2903 (m), 2867 (m), 2362 (w), 2345 (w), 2327 (w), 1587 (m), 1559 (m), 1476 (m), 1458 (m), 1415 (m), 1393 (m), 1361 (s), 1314 (w), 1285 (m), 1263 (m), 1245 (s), 1202 (m), 1115 (m, br), 1018 (m, br), 996 (m), 924 (w), 892 (m), 863 (vs), 838 (w), 802 (s, br), 712 (vs), 632 (w), 542 (m, br), 488 (w), 456 (w). ^{1}H-NMR (500 MHz, $CDCl_3$): δ (ppm) 1.21 [s, 54H, C(CH_3)$_3$], 7.33 [t, 3H, H_4, $^{4}J_{H\text{-}H}$ = 1.9 Hz, C_6H_3], 7.53 [d, 6H, $H_{2,6}$, $^{4}J_{H\text{-}H}$ = 1.9 Hz, C_6H_3]. ^{13}C{^{1}H}-NMR (125 MHz, $CDCl_3$): δ (ppm) 31.59 [s, C(CH$_3$)$_3$], 35.09 [s, C(CH$_3$)$_3$], 121.58 (s, C_4), 131.80 (s, $C_{2,6}$), 152.27 (s, $C_{3,5}$), 155.28 (s, C_1).

*Synthesis of (C_6H_3-t-Bu_2-3,5)$_2$BiCl (**5**) – chloro-bis(3,5-di-tert-butylphenyl)bismuthine*

A solution of $BiCl_3$ (0.803 g, 2.54 mmol, 1 equiv) in Et_2O (30 mL) was added dropwise to a solution of (C_6H_3-*t*-Bu_2-3,5)Li (prepared from1-bromo-3,5-di-tert-butylbenzene and *n*-BuLi) (1.500 g, 7.64 mmol, 3 equiv) in Et_2O (20 mL) at 0 °C. The resulting brown suspension was stirred for 24 h, filtered off and then the solvent was removed in vacuum. The crude product was extracted with toluene and filtered off to give **5** as a yellow crystalline product. Single crystals suitable for X-ray analyses were grown from a CH_2Cl_2 solution at −28 °C. Yield: 0.179 g (11% based on $BiCl_3$). M.p. = 207-209 °C. Elemental analysis calcd. (%) for $C_{28}H_{42}BiCl$ (623.07 g mol^{-1}): C, 53.97; H, 6.79. Found: C, 53.64; H, 6.88. ATR FTIR (cm^{-1}): $\tilde{\nu}$ = 2952 (s), 2904 (m), 2864 (m), 1789 (w), 1586 (m), 1561 (m), 1471 (m, br), 1424 (m), 1393 (m), 1357 (vs), 1315 (w), 1285 (m), 1250 (s), 1202 (m), 1021 (w), 991 (m), 931 (w), 895 (m), 865 (s), 835 (w), 733 (w), 703 (vs), 631 (w), 535 (m, br), 457 (w), 427 (w), 409 (w). ^{1}H-NMR (500 MHz, $CDCl_3$): δ (ppm) 1.31 [s, 36H, C(CH_3)$_3$], 7.41 [t, 2H, H_4, $^{4}J_{H\text{-}H}$ = 1.8 Hz, C_6H_3], 8.06 [d, 4H, $H_{2,6}$, $^{4}J_{H\text{-}H}$ = 1.8 Hz, C_6H_3]. ^{13}C{^{1}H}-NMR (125 MHz, $CDCl_3$): δ 31.62 [s, C(CH$_3$)$_3$], 35.71 [s, C(CH$_3$)$_3$], 123.33 (s, C_4), 130.90 (s, $C_{2,6}$), 154.15 (s, $C_{3,5}$), C_1 could not be detected.

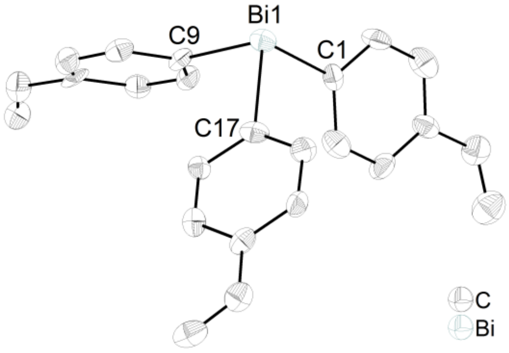

Figure S1. Molecular structure of $(C_6H_4\text{-}CH{=}CH_2\text{-}4)_3Bi$ (**2a**) showing the thermal ellipsoids that are set at 50% probability level. Hydrogen atoms were omitted for clarity. Selected bond lengths and distances [Å]: Bi1–C1 2.205(14), Bi1–C9 2.239(15), Bi1–C17 2.264(15). Selected bond angles [°]: C1–Bi1–C9 94.6(5), C1–Bi1–C17 94.5(5), C9–Bi1–C17 94.8(5).

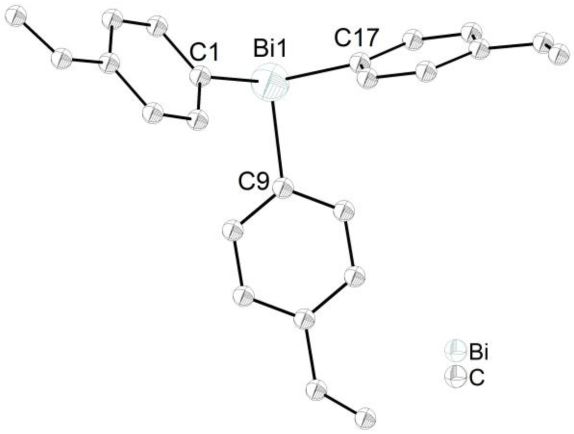

Figure S2. Molecular structure of $(C_6H_4\text{-}CH{=}CH_2\text{-}4)_3Bi$ (**2b**) showing the thermal ellipsoids that are set at 50% probability level. Hydrogen atoms were omitted for clarity. Selected bond lengths and distances [Å]: Bi1–C1 2.242(4), Bi1–C9 2.231(5), Bi1–C17 2.249(4). Selected bond angles [°]: C1–Bi1–C9 97.11(18), C1–Bi1–C17 98.19(15), C9–Bi1–C17 94.0(2).

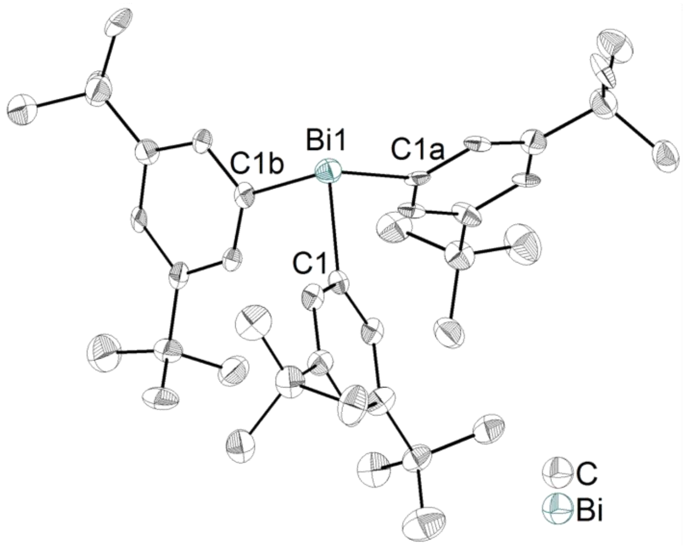

Figure S3. Molecular structure of $(C_6H_3$-*t*-Bu_2-3,5$)_3$Bi (**4**) showing the thermal ellipsoids that are set at 50% probability level. Hydrogen atoms were omitted for clarity. Symmetry transformations: a = –*y* + 1, *x* – *y* + 1, *z*; b = –*x* + *y*, –*x* + 1, *z*. Selected bond lengths [Å]: Bi1–C1 2.27(2), Bi1–C1a 2.27(2), Bi1–C1b 2.27(2). Selected bond angles [°]: C1–Bi1–C1a 94.8(8), C1–Bi1–C1b 94.8(8), C1a–Bi1–C1b 94.8(8).

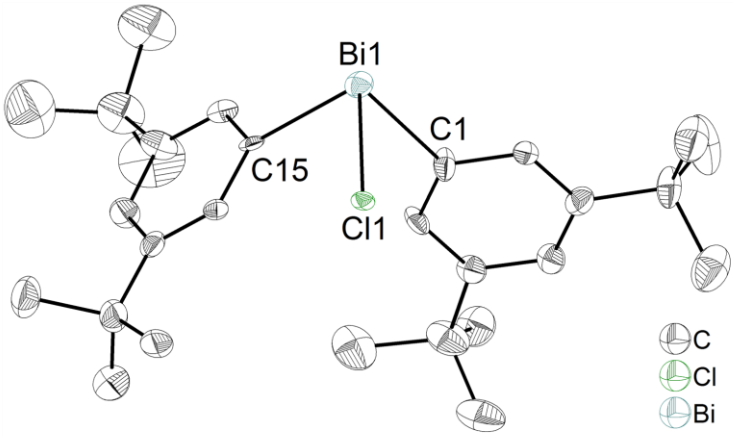

Figure S4. Molecular structure of $(C_6H_3$-*t*-Bu_2-3,5$)_2$BiCl (**5**) showing the thermal ellipsoids that are set at 50% probability level. Hydrogen atoms were omitted for clarity. Selected bond lengths and distances [Å]: Bi1–C1 2.268(16), Bi1–C15 2.280(19), Bi1–Cl1 2.811(7). Selected bond angles [°]: C1–Bi1–C15 92.4(10), C1–Bi1–Cl1 85.5(8), C1–Bi1–C1b 86.6(8), C15–Bi1–Cl1 90.9(10).

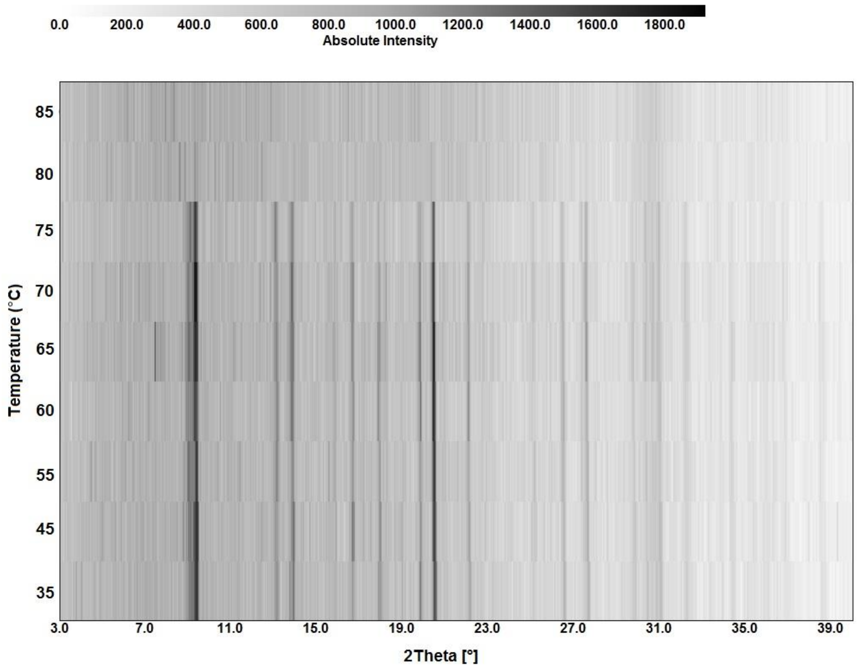

Figure S5. Temperature dependent PXRD of Ph_3Bi (**1a**) showing no phase transition up to 353 K.

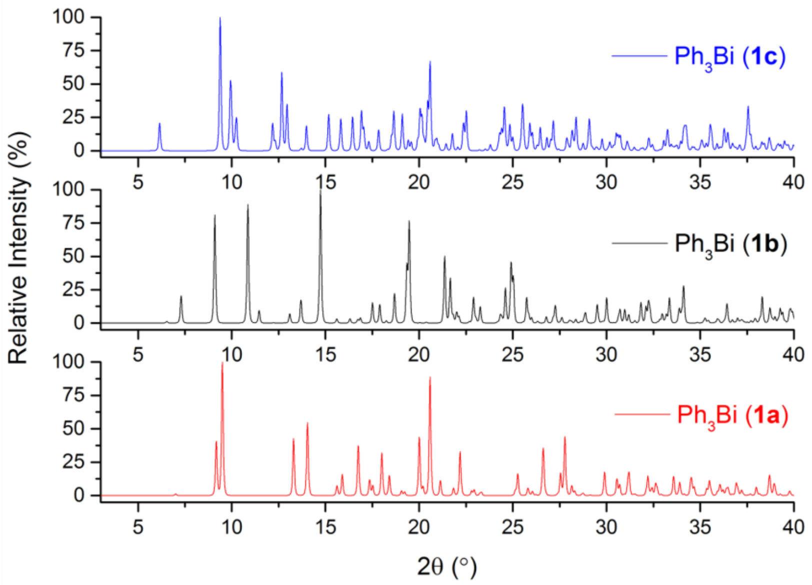

Figure S6. The PXRD pattern of the three Ph_3Bi polymorphs, calculated from the single crystal X-ray data: polymorph **1a** [29-33], polymorph **1b** [34] and polymorph **1c** [35].

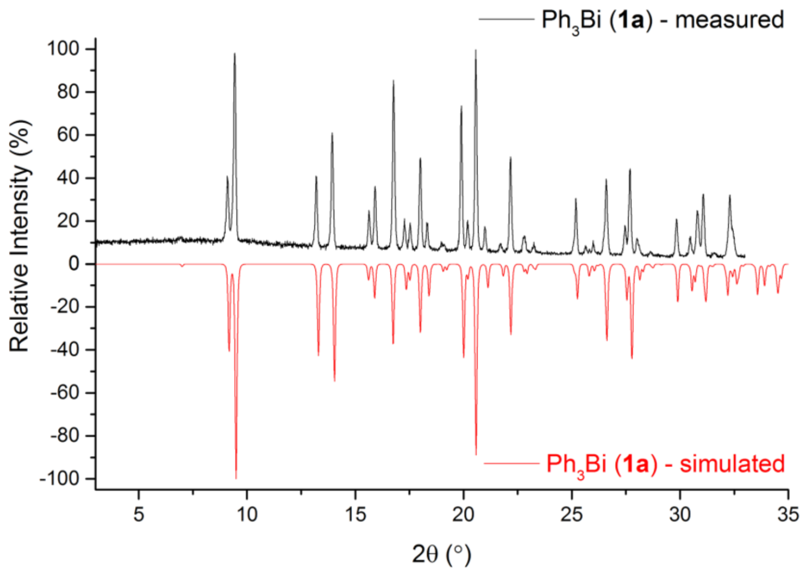

Figure S7. Powder X-ray diffraction pattern of Ph_3Bi polymorph **1a** at 293 K, measured for isolated material (black) and calculated from single crystal X-ray data measured at 143 K (red) [32].

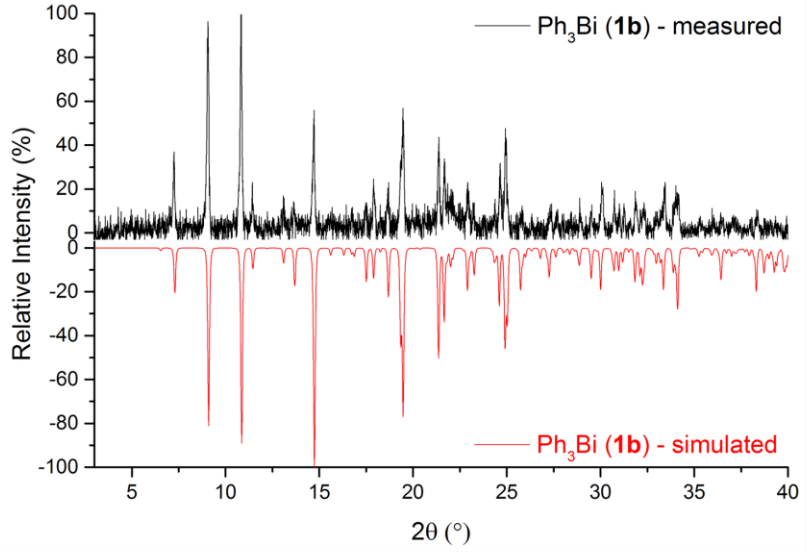

Figure S8. Powder X-ray diffraction pattern of Ph_3Bi polymorph **1b** at 103 K, measured for isolated material (black) and calculated from single crystal X-ray data measured at 123 K (red) [34].

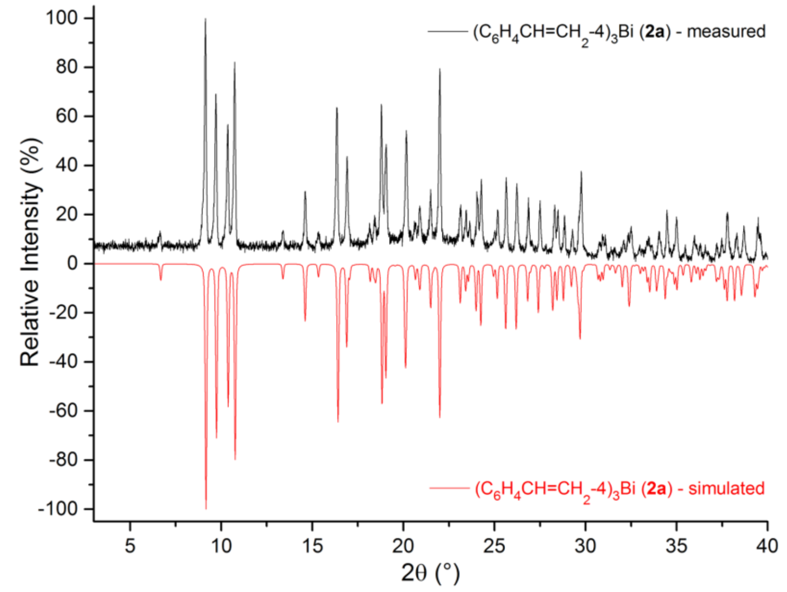

Figure S9. Powder X-ray diffraction pattern of polymorph $(C_6H_4\text{-}CH{=}CH_2\text{-}4)_3Bi$ (**2a**) at 120 K, measured for isolated material (black) and calculated from single crystal X-ray data measured at 120 K (red).

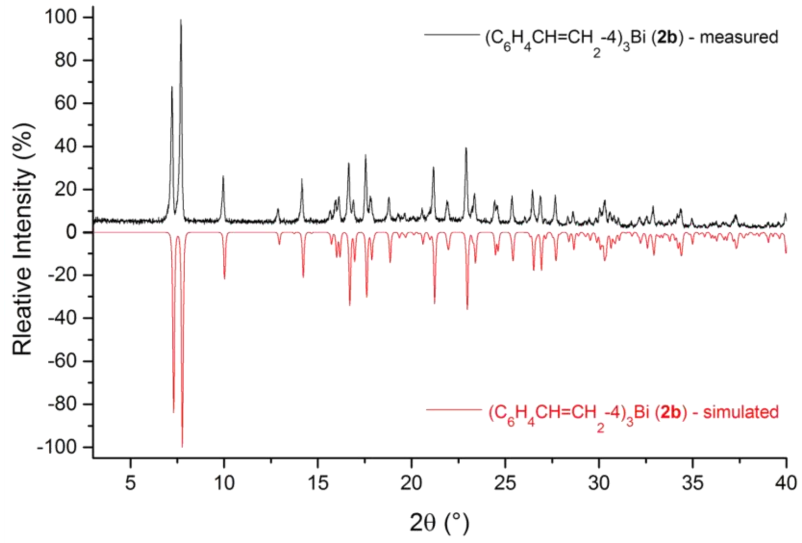

Figure S10. Powder X-ray diffraction pattern of polymorph $(C_6H_4\text{-}CH{=}CH_2\text{-}4)_3Bi$ (**2b**) at 100 K, measured for isolated material (black) and calculated from single crystal X-ray data measured at 100 K (red).

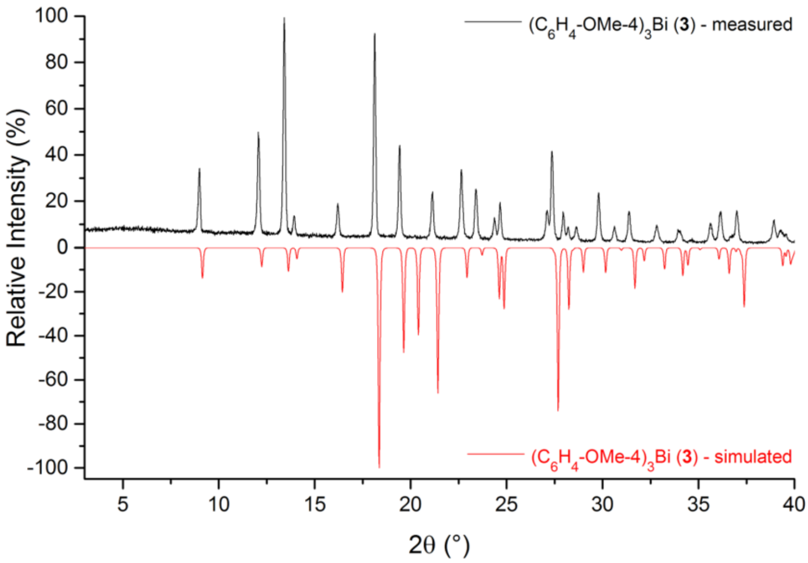

Figure S11. Powder X-ray diffraction pattern of $(C_6H_4\text{-OMe-4})_3Bi$ (**3**) at 293 K, measured for isolated material (black) and calculated from single crystal X-ray data measured at 120 K (red).

Table S1. Crystallographic data and structure refinement details for four polymorphs of Ph_3Bi (**1a**) [32], (**1b**) [34], (**1c**) [35] and (**1d**) [29].

	**1a**	**1b**	**1c**	**1d**
empirical formula	$C_{18}H_{15}Bi$	$C_{18}H_{15}Bi$	$C_{18}H_{15}Bi$	$C_{18}H_{15}Bi$
M (g mol^{-1})	440.29	440.29	440.29	440.29
temperature (K)	143	123	100	295
crystal system	monoclinic	orthorhombic	monoclinic	triclinic
space group	*C*2/*c*	*Pna*2$_1$	*P*2$_1$/*c*	$P\bar{1}$
a (Å)	27.515(6)	24.215(5)	15.3458(2)	19.4300
b (Å)	5.7278(15)	16.283(3)	11.2421(1)	17.7200
c (Å)	20.273(5)	7.643(2)	18.4304(2)	11.1400
α (deg)	90	90	90	80.15
β (deg)	114.30(3)	90	110.6431(14)	128.47
γ (deg)	90	90	90	99.97
V (Å^3)	2912.1(12)	3013.4(10)	2975.43(7)	2947.59
Z	8	8	8	8
D_{calc} (g cm^{-3})	2.008	1.941	1.966	1.984

Table S2. Crystallographic data and structure refinement details for Ph_3Bi (**1b**) [34], (C_6H_4-CH=CH_2-4)$_3$Bi (**2a**) and (C_6H_4-CH=CH_2-4)$_3$Bi (**2b**).

	**1b**	**2a**	**2b**
empirical formula	$C_{18}H_{15}Bi$	$C_{24}H_{21}Bi$	$C_{24}H_{21}Bi$
M (g mol^{-1})	440.28	518.39	518.39
temperature (K)	123	120	100
λ (Å)	0.71073	1.54184	0.71073
crystal system	orthorhombic	orthorhombic	monoclinic
space group	$Pna2_1$	$P2_12_12_1$	$P2_1/c$
a (Å)	24.215(5)	5.6530(1)	6.2446(7)
b (Å)	16.283(3)	18.0950(4)	24.188(3)
c (Å)	7.643(2)	19.2161(4)	12.9283(15)
α (deg)	90	90	90
β (deg)	90	90	94.620(3)
γ (deg)	90	90	90
V (Å^3)	3013.4(10)	1965.63(7)	1946.4(4)
Z	8	4	4
D_{calc} (g cm^{-3})	1.941	1.752	1.769
μ (mm^{-1})	11.686	17.597	9.061
F(000)	1648	992	992
crystal size (mm)	0.25 x 0.06 x 0.06	0.28 x 0.28 x 0.02	0.40x 0.20 x 0.20
reflections collected	8768	6071	20432
independent reflections	6952	3254	3420
R_{int}	0.0428	0.0594	0.0374
number of data / restraints / parameters	6952 / 1 / 343	3254 / 0 / 226	3420 / 114 / 239
goodness-of-fit on F^2	1.012	1.036	1.031
final R indices [$I > 2\sigma(I)$]			
R_1	0.0365	0.0489	0.0221
wR_2	0.0553	0.1238	0.0481
R indices (all data)			
R_1	0.0586	0.0506	0.0281
wR_2	0.0605	0.1255	0.0496
flack x parameter[36]	−0.003(6)	−0.029(19)	
$\Delta\rho_{min}$ and $\Delta\rho_{max}$ (e Å^{-3})	−1.523 and 1.557	−1.116 and 2.855	−1.188 and 1.453

Table S3. Crystallographic data and structure refinement details for $(C_6H_4\text{-OMe-4})_3Bi$ (**3**), $(C_6H_3\text{-}t\text{-Bu}_2\text{-3,5})_3Bi$ (**4**) and $(C_6H_3\text{-}t\text{-Bu}_2\text{-3,5})_2BiCl$ (**5**)·$2CH_2Cl_2$.

	**3**	**4**	**5**·$2CH_2Cl_2$
empirical formula	$C_{21}H_{21}BiO_3$	$C_{42}H_{63}Bi$	$C_{30}H_{46}BiCl_5$
M (g mol^{-1})	530.36	776.90	792.90
temperature (K)	120	115	115
λ (Å)	0.71073	0.71073	0.71073
crystal system	trigonal	hexagonal	orthorhombic
space group	$R\overline{3}$	$P6_3$	$Pna2_1$
a (Å)	12.9834(3)	41.741(2)	21.581(2)
b (Å)	12.9834(3)	41.741(2)	17.1008(10)
c (Å)	18.8685(6)	9.1220(5)	9.3162(5)
α (deg)	90	90	90
β (deg)	90	90	90
γ (deg)	120	120	90
V (Å^3)	2754.51(15)	13763.9(18)	3438.2(5)
Z	6	14	4
D_{calc} (g cm^{-3})	1.918	1.312	1.532
μ (mm^{-1})	9.586	4.508	5.534
F(000)	1524	5572	1576
crystal size (mm)	0.40 x 0.40 x 0.40	0.20 x 0.20 x 0.20	0.40 x 0.02 x 0.01
reflections collected	3293	52507	11032
independent reflections	1079	16028	11023
R_{int}	0.0179	0.1436	0.0985
number of data / restraints / parameters	1079 / 0 / 77	16028 / 841 / 928	11023 / 385 / 332
goodness-of-fit on F^2	1.079	1.072	0.958
final R indices [$I > 2\sigma(I)$]			
R_1	0.0190	0.0971	0.0688
wR_2	0.0470	0.2044	0.1481
R indices (all data)			
R_1	0.0198	0.1428	0.1329
wR_2	0.0473	0.2318	0.1753
flack x parameter[36]	-	−0.014(13)	0.34(2)
$\Delta\rho_{min}$ and $\Delta\rho_{max}$ (e Å^{-3})	−0.531 and 0.740	−2.064 and 5.617	−2.129 and 2.006

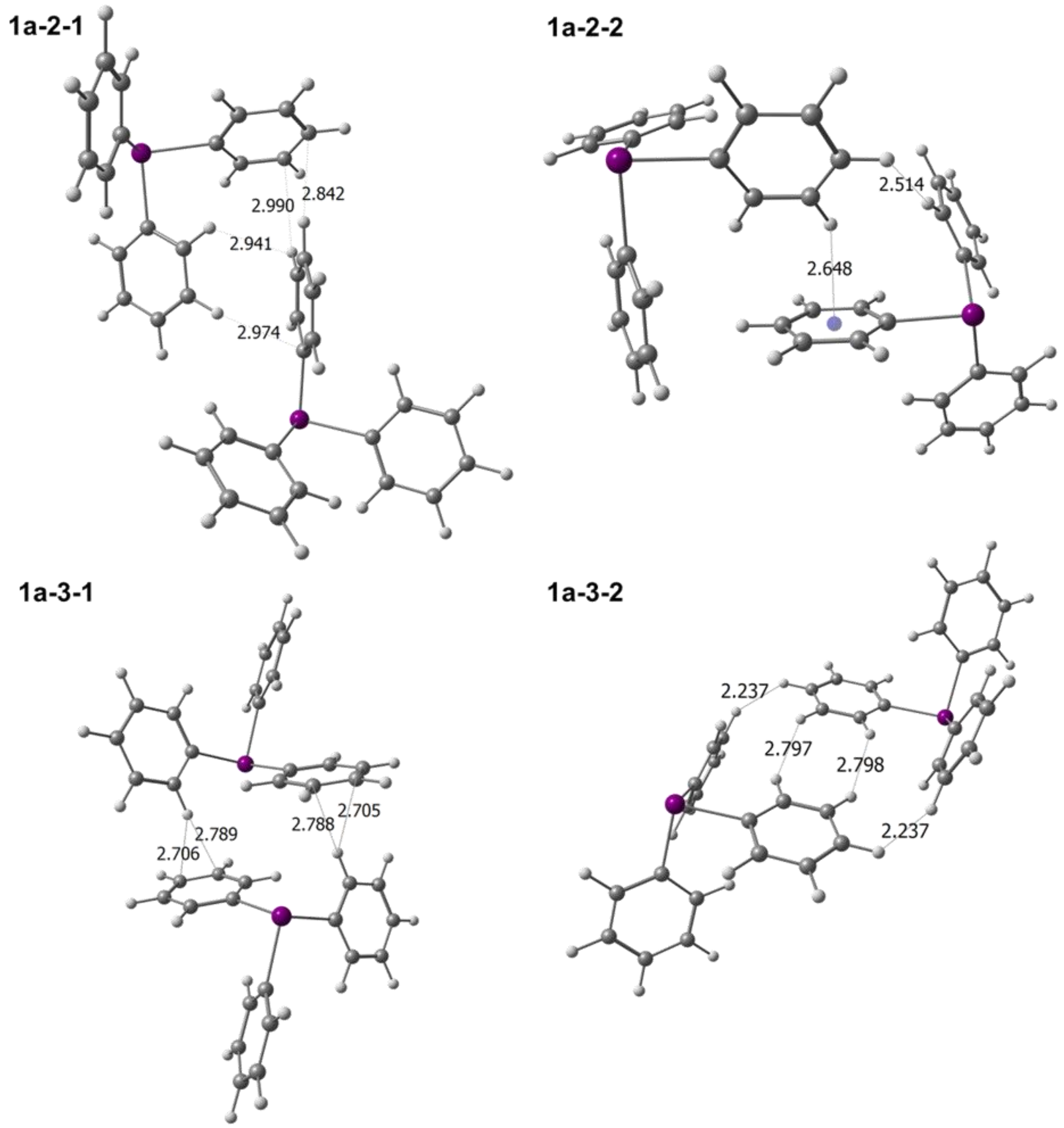

Figure S12. Structures of π stacking dimers found for polymorph **1a** of $BiPh_3$.

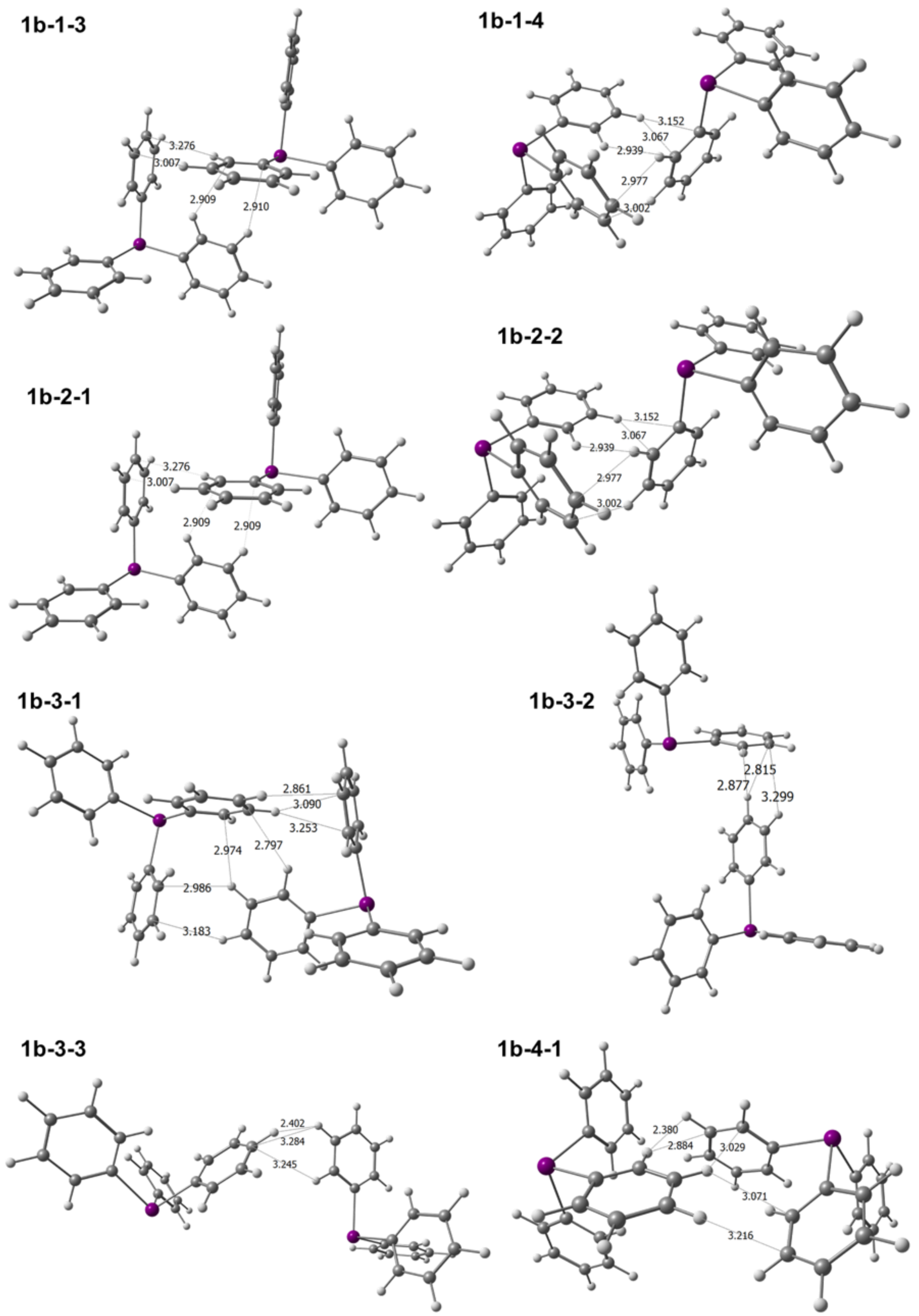

Figure S13. Structures of π stacking dimers found for polymorph **1b** of $BiPh_3$.

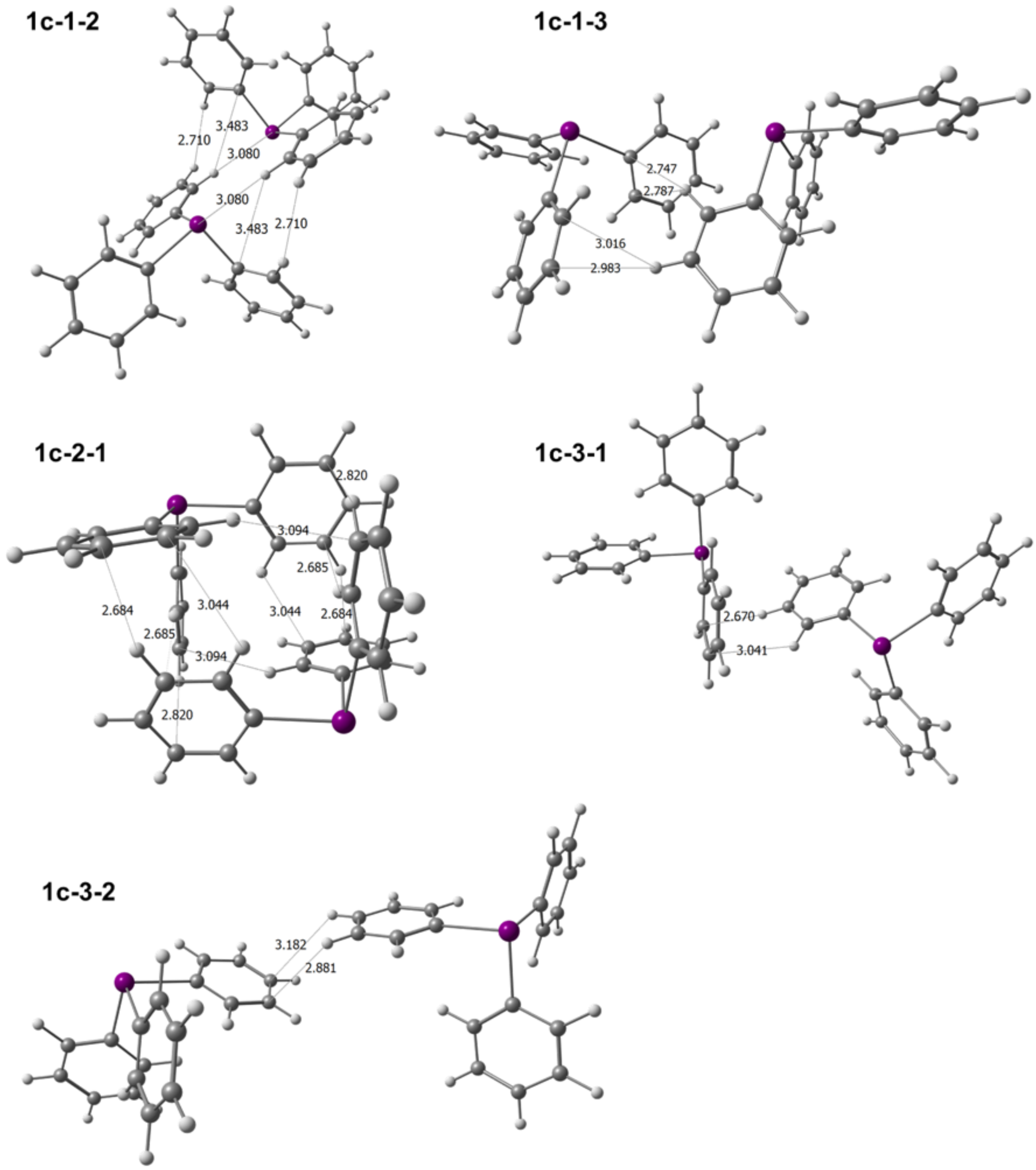

Figure S14. Structures of π stacking dimers found for polymorph **1c** of $BiPh_3$.

Table S4. Interaction energies (with respect to $BiPh_3$ in crystal geometry) and total energies (with respect to fully relaxed $BiPh_3$) in kJ mol^{-1} of π stacking dimers computed at the PBE-D3/def2-TZVP and DLPNO-CCSD(T)/cc-pVQZ (cc-pwCVQZ for Bi, TightPNO settings) level of theory.

Dimer	E(int) PBE-D3	E(tot) PBE-D3	E(int) DLPNO-CCSD(T)	E(tot) DLPNO-CCSD(T)	E(disp) DLPNO-CCSD(T)
**1a-2-1**	-29.19	4.10			
**1a-2-2**	-29.20	4.20			
**1a-3-1**	-42.38	-9.12	-42.83	-15.86	-49.63
**1a-3-2**	-15.11	18.23			
**1b-1-3**	-27.48	-9.83	-27.64	-16.17	-29.79
**1b-1-4**	-28.74	-6.15			
**1b-2-1**	-27.48	-9.84			
**1b-2-2**	-28.75	-6.12			
**1b-3-1**	-29.80	-9.65	-29.19	-14.90	-35.82
**1b-3-2**	-15.65	1.97			
**1b-3-3**	-8.02	12.07			
**1b-4-1**	-28.40	-8.20			
**1c-1-2**	-20.36	-9.70	-18.27	-13.89	-31.08
**1c-1-3**	-38.42	-25.88	-37.74	-31.54	-47.22
**1c-2-1**	-63.90	-49.31	-64.37	-56.35	-82.75
**1c-3-1**	-19.71	-5.27			
**1c-3-2**	-10.61	1.92			

Cartesian coordinates

C_6H_6

```
C      -0.000007000      1.397058000      0.000000000
C      -0.000007000      0.698534000      1.209865000
C       0.000000000     -0.698501000      1.209905000
C       0.000007000     -1.397059000      0.000000000
C       0.000007000     -0.698535000     -1.209865000
C       0.000000000      0.698501000     -1.209905000
H      -0.000013000      2.488482000      0.000093000
H      -0.000013000      1.244246000      2.155040000
H       0.000000000     -1.244111000      2.155164000
H       0.000013000     -2.488482000     -0.000092000
H       0.000013000     -1.244246000     -2.155039000
H       0.000000000      1.244111000     -2.155163000
```

$BiPh_3$

```
Bi      3.500000000      0.000000000      0.000000000
C       4.719687000     -1.913701000     -0.001821000
C       4.307770000     -2.971301000      0.819578000
C       5.042549000     -4.159973000      0.868315000
C       6.193168000     -4.301103000      0.089307000
C       6.606111000     -3.252337000     -0.735603000
C       5.872850000     -2.062229000     -0.781188000
C       4.720728000      0.956527000     -1.656843000
C       4.325665000      0.746344000     -2.984492000
C       5.062011000      1.293758000     -4.039510000
C       6.197709000      2.061901000     -3.772933000
C       6.593784000      2.279554000     -2.451393000
C       5.858852000      1.728739000     -1.396493000
C       4.717431000      0.957036000      1.658310000
C       5.843154000      0.336810000      2.212365000
C       6.571356000      0.971437000      3.224016000
```

C	6.181387000	2.230136000	3.687106000
C	5.058946000	2.854466000	3.138354000
C	4.328734000	2.217646000	2.130354000
H	3.452754000	2.720927000	1.711235000
H	4.749280000	3.838574000	3.495894000
H	6.751375000	2.724505000	4.475687000
H	7.448287000	0.479415000	3.649939000
H	6.160845000	-0.644804000	1.853864000
H	6.180946000	1.901006000	-0.367141000
H	7.481019000	2.879287000	-2.237819000
H	6.773417000	2.490972000	-4.594901000
H	4.747322000	1.120358000	-5.070607000
H	3.439330000	0.147061000	-3.211667000
H	6.207009000	-1.246988000	-1.426606000
H	7.504843000	-3.358522000	-1.346748000
H	6.767121000	-5.228735000	0.124699000
H	4.714452000	-4.976579000	1.514622000
H	3.409155000	-2.877622000	1.436059000

Tetramer **1a-1**

Bi	6.684281000	4.367542000	8.709206000
C	5.012351000	4.613491000	7.243799000
C	4.056788000	3.624383000	7.105140000
C	3.041715000	3.729878000	6.147980000
C	2.996296000	4.826779000	5.316540000
C	4.947676000	5.743044000	6.412359000
C	8.117651000	5.596120000	7.441709000
C	8.668177000	1.053925000	7.885097000
C	9.548474000	1.756669000	7.081415000
C	9.872592000	1.285268000	5.821305000
C	9.331510000	0.087073000	5.374107000
C	8.460437000	5.110978000	6.188990000
C	6.033512000	0.386238000	10.002372000
C	4.791258000	0.970003000	9.889598000
C	4.421963000	1.998727000	10.713668000
C	5.314186000	2.484923000	11.650512000
C	6.554939000	1.901157000	11.790996000
C	6.912142000	0.852385000	10.963235000
C	9.331985000	5.814873000	5.374228000
C	3.924101000	5.826199000	5.449658000
C	8.117176000	-0.131680000	7.441588000
C	6.033987000	6.114038000	10.002493000
C	4.791732000	6.697803000	9.889720000
C	6.912617000	6.580185000	10.963357000
C	4.422437000	7.726527000	10.713789000
C	6.555414000	7.628957000	11.791117000
C	5.314661000	8.212723000	11.650634000
C	8.668652000	6.781725000	7.885218000
C	8.459962000	-0.616822000	6.188869000
Bi	6.683807000	-1.360258000	8.709085000
C	9.873067000	7.013068000	5.821426000
C	9.548949000	7.484469000	7.081537000
C	5.011876000	-1.114309000	7.243678000
C	4.056313000	-2.103417000	7.105019000
C	4.947201000	0.015244000	6.412238000
C	3.041240000	-1.997922000	6.147859000
C	3.923626000	0.098399000	5.449537000
C	2.995822000	-0.901021000	5.316419000
C	4.057262000	9.352183000	7.105261000
C	3.042190000	9.457678000	6.148101000
C	2.996771000	10.554579000	5.316662000
C	3.924576000	11.553999000	5.449779000
C	4.948150000	11.470834000	6.412480000
C	3.923152000	-5.629401000	5.449416000
C	5.012826000	10.341291000	7.243920000
C	2.995347000	-6.628821000	5.316298000
C	4.946726000	-5.712556000	6.412117000
Bi	6.684756000	10.095342000	8.709327000
C	3.040765000	-7.725722000	6.147738000
C	5.011402000	-6.842109000	7.243557000
C	4.055838000	-7.831217000	7.104897000
C	8.118125000	11.323920000	7.441830000
C	6.034462000	11.841838000	10.002614000
Bi	6.683332000	-7.088058000	8.708963000
C	8.116701000	-5.859480000	7.441467000

C	6.033038000	-5.341562000	10.002251000
C	8.667702000	-4.673875000	7.884976000
C	8.459487000	-6.344622000	6.188748000
C	4.790783000	-4.757796000	9.889477000
C	6.911668000	-4.875415000	10.963114000
C	9.331035000	-5.640727000	5.373986000
C	4.421488000	-3.729073000	10.713546000
C	9.872117000	-4.442532000	5.821184000
C	5.313711000	-3.242877000	11.650391000
C	8.669127000	12.509525000	7.885339000
C	8.460912000	10.838778000	6.189111000
C	9.548000000	-3.971131000	7.081294000
C	9.332460000	11.542663000	5.374349000
C	9.873542000	12.740868000	5.821548000
C	4.792207000	12.425603000	9.889841000
C	6.913092000	12.307985000	10.963478000
C	6.554464000	-3.826643000	11.790875000
C	4.422912000	13.454327000	10.713910000
C	5.315135000	13.940523000	11.650755000
C	6.555888000	13.356757000	11.791238000
C	9.549424000	13.212269000	7.081658000
H	4.090090000	2.733376000	7.738154000
H	2.304833000	2.929044000	6.057769000
H	2.213802000	4.906033000	4.558306000
H	5.686467000	6.541887000	6.490943000
H	8.404735000	1.455583000	8.866300000
H	9.971370000	2.701177000	7.432183000
H	10.556424000	1.851282000	5.185590000
H	9.584062000	-0.295946000	4.382787000
H	8.034649000	4.172529000	5.821762000
H	4.086670000	0.606001000	9.135745000
H	3.434968000	2.456729000	10.613344000
H	5.030133000	3.323522000	12.289682000
H	7.257578000	2.275788000	12.538691000
H	7.898003000	0.392471000	11.086133000
H	9.584597000	5.431709000	4.382956000
H	3.883912000	6.704633000	4.800959000
H	4.087343000	6.333978000	9.135589000
H	7.898582000	6.120359000	11.085946000
H	3.435829000	8.185236000	10.612915000
H	7.258293000	8.004154000	12.538326000
H	5.031070000	9.052174000	12.288857000
H	8.404815000	7.183725000	8.866158000
H	8.033657000	-1.554944000	5.821372000
H	10.556708000	7.579273000	5.185620000
H	9.971434000	8.429222000	7.432147000
H	4.089126000	-2.993989000	7.738678000
H	5.686105000	0.813986000	6.490740000
H	2.303718000	-2.798223000	6.058100000
H	3.883426000	0.976759000	4.800758000
H	2.213225000	-0.821713000	4.558334000
H	4.090933000	8.460911000	7.737892000
H	2.305495000	8.656720000	6.057346000
H	2.214028000	10.633929000	4.558757000
H	3.883668000	12.429470000	4.796808000
H	5.688117000	12.268581000	6.492923000
H	3.882713000	-4.750554000	4.801463000
H	2.213307000	-6.550565000	4.557794000
H	5.684907000	-4.913273000	6.491430000
H	2.300781000	-8.523423000	6.056382000
H	4.083246000	-8.720648000	7.742038000
H	8.405102000	-4.272842000	8.866538000
H	8.029616000	-7.280600000	5.819054000
H	4.085403000	-5.121690000	9.136486000
H	7.897465000	-5.334671000	11.087166000
H	9.584592000	-6.024485000	4.383529000
H	3.434233000	-3.272014000	10.613057000
H	10.556713000	-3.877365000	5.185732000
H	5.029224000	-2.405017000	12.290270000
H	8.399091000	12.915059000	8.863582000
H	8.035980000	9.899711000	5.822421000
H	9.970636000	-3.027067000	7.433084000
H	9.585385000	11.159802000	4.383012000
H	10.559087000	13.303830000	5.184912000
H	4.088767000	12.065846000	9.133425000
H	7.898212000	11.846565000	11.088109000
H	7.257054000	-3.453485000	12.539116000
H	3.434003000	13.908350000	10.615193000

H	5.027284000	14.771373000	12.298692000
H	7.257906000	13.727620000	12.541620000
H	9.976202000	14.153511000	7.436856000

Tetramer **1a-2**

H	11.276220000	-0.390240000	7.392009000
H	10.775907000	-1.459387000	9.617262000
H	9.198438000	-2.697828000	11.043480000
H	8.615640000	-1.848370000	13.295782000
H	9.591852000	0.294755000	14.098690000
H	11.140326000	1.553736000	12.660355000
C	12.261232000	-0.851645000	7.517069000
C	10.505258000	-1.053719000	10.595207000
C	9.624966000	-1.756596000	11.398769000
C	9.300882000	-1.285421000	12.658982000
C	9.841993000	-0.087316000	13.106388000
C	10.713525000	0.616714000	12.291736000
C	13.139889000	-0.385688000	8.478000000
C	12.618401000	-1.900272000	6.689110000
H	15.085506000	-0.609920000	9.347157000
H	15.740103000	-2.452314000	7.865335000
H	14.146933000	-3.314755000	6.181359000
H	11.916341000	-2.271013000	5.938805000
C	11.056288000	0.131796000	11.038921000
Bi	12.489640000	1.360582000	9.771619000
C	14.382137000	-0.969494000	8.590640000
C	13.859148000	-2.484083000	6.829461000
C	14.751398000	-1.998073000	7.766376000
H	11.276114000	5.335561000	7.393782000
H	10.769957000	4.272147000	9.615741000
H	9.204789000	3.025921000	11.049505000
H	8.619940000	3.875722000	13.297390000
H	9.587481000	6.023603000	14.098008000
H	11.146695000	7.278581000	12.664086000
C	12.261799000	4.876154000	7.518237000
C	10.505825000	4.674081000	10.596375000
C	9.625533000	3.971204000	11.399937000
C	9.301449000	4.442379000	12.660150000
C	9.842560000	5.640484000	13.107556000
C	10.714092000	6.344514000	12.292904000
C	13.140456000	5.342112000	8.479167000
C	12.618968000	3.827528000	6.690278000
H	15.086957000	5.120641000	9.346537000
H	15.738296000	3.271237000	7.869516000
H	14.143133000	2.404331000	6.192409000
H	11.916212000	3.454059000	5.942413000
C	11.056855000	5.859596000	11.040089000
Bi	12.490207000	7.088382000	9.772787000
C	14.382704000	4.758306000	8.591808000
C	13.859715000	3.243716000	6.830628000
C	14.751965000	3.729727000	7.767543000
C	14.162174000	6.842138000	11.238114000
C	15.117746000	7.831205000	11.376924000
C	14.226849000	5.712432000	12.069347000
H	15.086566000	8.723899000	10.744641000
C	16.132837000	7.725519000	12.334054000
C	15.250442000	5.629084000	13.032011000
H	13.486247000	4.915263000	12.003451000
H	16.863910000	8.529033000	12.436869000
C	16.178255000	6.628465000	13.165292000
H	15.279049000	4.756235000	13.687969000
H	16.956175000	6.565191000	13.929803000
H	15.079770000	2.997043000	10.746805000
H	16.862079000	2.802352000	12.434552000
H	16.957073000	0.833356000	13.926342000
H	13.485192000	-0.812222000	11.988823000
C	15.117179000	2.103405000	11.375757000
C	16.132270000	1.997720000	12.332886000
C	14.161607000	1.114338000	11.236947000
C	14.226282000	-0.015368000	12.068179000
C	15.249875000	-0.098715000	13.030843000
C	16.177688000	0.900666000	13.164124000
H	15.288625000	-0.971775000	13.687109000
H	13.477995000	7.619499000	14.029315000
H	13.477391000	1.894417000	14.023963000

C	13.508789000	8.492820000	14.684365000
H	13.678220000	5.859788000	16.968556000
H	11.895403000	5.665672000	15.283930000
H	11.801959000	3.700879000	13.786487000
H	15.271742000	2.050084000	15.715294000
C	13.508222000	2.765020000	14.683197000
C	12.581173000	9.492439000	14.551491000
C	14.532398000	8.575572000	15.647063000
H	13.669566000	11.584249000	16.976635000
H	11.888187000	11.388213000	15.292209000
H	11.800441000	9.418344000	13.790346000
H	15.270399000	7.776286000	15.715523000
C	13.641353000	4.966439000	16.339171000
C	12.626241000	4.861345000	15.382009000
C	12.580606000	3.764639000	14.550323000
C	14.531831000	2.847773000	15.645895000
C	12.626808000	10.589145000	15.383177000
C	14.597297000	9.704926000	16.478756000
C	13.641920000	10.694239000	16.340339000
C	14.596730000	3.977127000	16.477588000
Bi	16.269312000	9.950242000	17.944183000
Bi	16.268745000	4.222442000	17.943015000
C	17.702421000	8.721689000	16.676380000
C	15.618727000	8.203573000	19.237091000
C	17.701854000	2.993889000	16.675212000
C	15.618160000	2.475773000	19.235923000
C	18.253226000	7.535884000	17.119610000
C	18.045280000	9.207050000	15.423763000
C	14.376354000	7.620059000	19.124212000
C	16.497282000	7.737050000	20.197831000
C	18.252659000	1.808084000	17.118443000
C	18.044713000	3.479250000	15.422595000
C	14.375787000	1.892259000	19.123044000
C	16.496716000	2.009250000	20.196663000
H	17.989431000	7.133716000	18.100260000
C	19.133369000	6.833161000	16.315753000
C	18.916673000	8.503180000	14.608824000
H	17.614045000	10.142282000	15.053841000
H	13.672060000	7.982619000	18.369674000
C	14.006889000	6.591217000	19.948057000
C	16.139905000	6.688157000	21.025363000
H	17.483012000	8.196280000	20.322624000
H	17.981916000	1.402173000	18.096218000
C	19.132802000	1.105361000	16.314585000
C	18.916106000	2.775380000	14.607656000
H	17.616986000	4.416480000	15.055520000
H	13.673088000	2.251597000	18.365800000
C	14.006322000	0.863418000	19.946889000
C	16.139338000	0.960357000	21.024195000
H	17.481600000	2.470817000	20.322422000
H	19.554919000	5.888286000	16.666406000
C	19.457556000	7.304785000	15.055742000
H	19.174789000	8.887246000	13.619511000
H	13.020451000	6.133028000	19.845915000
C	14.899053000	6.104648000	20.884774000
H	16.842523000	6.314364000	21.773192000
H	19.558872000	0.163501000	16.668623000
C	19.456989000	1.576986000	15.054574000
H	19.171280000	3.157983000	13.616692000
H	13.017695000	0.409209000	19.847331000
C	14.898486000	0.376848000	20.883606000
H	16.841268000	0.589257000	21.774440000
H	20.140444000	6.739024000	14.419046000
H	14.615384000	5.265180000	21.522771000
H	20.140178000	1.013321000	14.416304000
H	14.610585000	-0.454090000	21.531295000

Tetramer **1a-3**

Bi	6.681388000	4.372274000	8.705861000
C	5.008378000	4.616682000	7.241429000
C	4.053452000	3.626846000	7.103572000
C	3.037714000	3.731390000	6.147014000
C	2.990984000	4.828076000	5.315363000
C	4.942366000	5.746006000	6.409782000
C	8.113079000	5.601622000	7.437216000

C	8.667197000	1.059927000	7.881255000
C	9.546486000	1.763138000	7.076879000
C	9.870174000	1.291700000	5.816673000
C	9.329693000	0.093011000	5.370068000
C	8.455450000	5.116457000	6.184391000
C	6.034323000	0.390779000	10.000295000
C	4.791573000	0.973612000	9.888158000
C	4.422033000	2.002245000	10.712229000
C	5.314476000	2.489297000	11.648419000
C	6.555741000	1.906469000	11.788267000
C	6.913202000	0.857777000	10.960516000
C	9.325983000	5.820810000	5.368940000
C	3.918140000	5.828203000	5.447693000
C	8.116790000	-0.126177000	7.438343000
C	6.030613000	6.118577000	9.999167000
C	4.787863000	6.701410000	9.887030000
C	6.909492000	6.585576000	10.959388000
C	4.418323000	7.730044000	10.711102000
C	6.552031000	7.634267000	11.787140000
C	5.310766000	8.217096000	11.647292000
C	8.663487000	6.787726000	7.880127000
C	8.459160000	-0.611342000	6.185519000
Bi	6.685098000	-1.355525000	8.706989000
C	9.866464000	7.019498000	5.815545000
C	9.542776000	7.490937000	7.075751000
C	5.012088000	-1.111117000	7.242556000
C	4.057162000	-2.100953000	7.104700000
C	4.946076000	0.018207000	6.410910000
C	3.041425000	-1.996409000	6.148142000
C	3.921850000	0.100404000	5.448821000
C	2.994694000	-0.899723000	5.316491000
H	4.087546000	2.736442000	7.737440000
H	2.300100000	2.931055000	6.058088000
H	2.206885000	4.907311000	4.558905000
H	5.679770000	6.545930000	6.491203000
H	8.411671000	1.452925000	8.867449000
H	9.980698000	2.700218000	7.432720000
H	10.557876000	1.848824000	5.178669000
H	9.586881000	-0.291768000	4.380979000
H	8.030559000	4.176972000	5.818676000
H	4.085179000	0.609796000	9.135958000
H	3.435468000	2.460383000	10.610442000
H	5.029461000	3.328379000	12.286671000
H	7.258556000	2.269282000	12.539892000
H	7.896715000	0.398570000	11.088104000
H	9.582099000	5.437696000	4.378565000
H	3.875353000	6.703790000	4.794999000
H	4.083081000	6.341445000	9.131844000
H	7.893184000	6.127121000	11.085799000
H	3.429363000	8.183786000	10.611832000
H	7.257771000	8.002178000	12.535632000
H	5.021639000	9.048066000	12.294752000
H	8.399025000	7.188253000	8.861763000
H	8.032369000	-1.549429000	5.817851000
H	10.555772000	7.579434000	5.180742000
H	9.969866000	8.432487000	7.430190000
H	4.085704000	-2.990246000	7.742112000
H	5.681615000	0.819748000	6.491301000
H	2.301555000	-2.794349000	6.057635000
H	3.879384000	0.979584000	4.801425000
H	2.211494000	-0.821626000	4.559180000
H	11.279186000	-0.399322000	7.391081000
H	10.773350000	-1.460451000	9.615118000
H	9.202503000	-2.704686000	11.046680000
H	8.616600000	-1.851637000	13.296137000
H	9.590272000	0.290103000	14.098317000
H	11.141798000	1.550831000	12.658202000
C	12.262879000	-0.857776000	7.517492000
C	10.508884000	-1.059926000	10.596754000
C	9.629595000	-1.763137000	11.401119000
C	9.305907000	-1.291699000	12.661336000
C	9.846388000	-0.093010000	13.107940000
C	10.716911000	0.611343000	12.292489000
C	13.141758000	-0.390778000	8.477714000
C	12.620340000	-1.906468000	6.689741000
H	15.089290000	-0.613645000	9.345037000
H	15.743008000	-2.455986000	7.865048000
H	14.150731000	-3.320267000	6.182129000

H	11.914600000	-2.274378000	5.941248000
C	11.059291000	0.126178000	11.039665000
Bi	12.490973000	1.355526000	9.771020000
C	14.384508000	-0.973611000	8.589851000
C	13.861605000	-2.489296000	6.829589000
C	14.754048000	-2.002244000	7.765779000
H	11.275656000	5.329229000	7.388776000
H	10.760703000	4.274878000	9.609431000
H	9.191672000	3.027583000	11.044151000
H	8.614496000	3.878973000	13.298210000
H	9.585490000	6.019566000	14.095902000
H	11.139989000	7.277231000	12.659027000
C	12.259169000	4.870023000	7.516365000
C	10.505174000	4.667873000	10.595626000
C	9.625885000	3.964661000	11.399992000
C	9.302197000	4.436100000	12.660208000
C	9.842678000	5.634788000	13.106812000
C	10.713201000	6.339142000	12.291361000
C	13.138048000	5.337021000	8.476586000
C	12.616630000	3.821331000	6.688613000
H	15.087192000	5.118004000	9.340922000
H	15.736903000	3.267417000	7.866439000
H	14.142910000	2.399420000	6.190210000
H	11.913815000	3.458518000	5.936989000
C	11.055581000	5.853977000	11.038537000
Bi	12.487263000	7.083325000	9.769892000
C	14.380798000	4.754188000	8.588723000
C	13.857895000	3.238502000	6.828461000
C	14.750338000	3.725555000	7.764651000
C	14.160283000	6.838917000	11.234324000
C	15.115199000	7.828752000	11.372171000
C	14.226295000	5.709593000	12.065971000
H	15.086658000	8.718045000	10.734758000
C	16.130936000	7.724209000	12.328739000
C	15.250521000	5.627396000	13.028060000
H	13.490755000	4.908052000	11.985578000
H	16.870804000	8.522149000	12.419248000
C	16.177667000	6.627522000	13.160390000
H	15.292988000	4.748215000	13.675455000
H	16.960867000	6.549432000	13.917702000
H	15.084816000	2.991357000	10.739430000
H	16.872259000	2.796746000	12.418795000
H	16.965476000	0.820495000	13.917977000
H	13.492601000	-0.818130000	11.985677000
C	15.118909000	2.100954000	11.373298000
C	16.134646000	1.996410000	12.329867000
C	14.163993000	1.111118000	11.235452000
C	14.230005000	-0.018206000	12.067098000
C	15.254231000	-0.100403000	13.029188000
C	16.181377000	0.899724000	13.161517000
H	15.297019000	-0.975991000	13.681880000

Tetramer **1b-1**

Bi	5.586225000	12.516271000	0.600177000
C	7.716869000	14.894117000	0.167242000
C	5.108771000	14.080533000	2.164330000
C	4.057257000	14.922971000	1.887715000
C	3.654009000	15.880283000	2.823879000
C	4.290587000	15.966029000	4.052040000
C	5.338876000	15.112853000	4.324953000
C	5.746121000	14.171938000	3.399313000
C	6.946575000	11.329271000	1.950393000
C	6.429918000	10.214110000	2.599157000
C	7.220959000	9.470197000	3.471463000
C	8.534725000	9.816353000	3.686076000
C	9.068148000	10.922955000	3.035105000
C	8.269130000	11.675252000	2.168778000
C	7.285567000	13.661006000	-0.317879000
C	8.761942000	15.577928000	-0.451369000
C	7.931614000	13.130035000	-1.451101000
C	9.401793000	15.033735000	-1.543574000
C	8.981552000	13.814021000	-2.048751000
C	2.800520000	9.957303000	0.227815000
C	-0.781968000	14.852775000	0.142302000
Bi	2.256104000	12.744181000	-3.304725000

C	0.826344000	13.686948000	-1.815463000
C	1.284214000	14.799991000	-1.099990000
C	0.482206000	15.360088000	-0.118909000
C	-1.233003000	13.765901000	-0.578787000
C	-0.428134000	13.185222000	-1.563979000
C	0.763995000	11.297928000	-4.188857000
C	0.536356000	10.043004000	-3.666724000
C	-0.321503000	9.146155000	-4.300263000
C	-0.965806000	9.513496000	-5.469058000
C	-0.763062000	10.758552000	-5.997888000
C	0.123998000	11.647768000	-5.378922000
C	2.997393000	11.217616000	-1.817464000
C	4.157035000	10.513094000	-2.108591000
C	4.638199000	9.531370000	-1.257830000
C	3.958037000	9.251615000	-0.091212000
C	2.802234000	9.958132000	-7.414685000
C	2.336376000	10.936271000	-0.632273000
C	5.110485000	14.081360000	-5.478170000
C	5.747834000	14.172766000	-4.243187000
C	6.948289000	11.330099000	-5.692107000
C	6.431632000	10.214938000	-5.043343000
C	7.222673000	9.471025000	-4.171036000
C	8.536438000	9.817182000	-3.956424000
C	9.069862000	10.923784000	-4.607394000
C	8.270844000	11.676079000	-5.473722000
C	3.959751000	9.252444000	-7.733712000
C	2.338089000	10.937098000	-8.274773000
Bi	5.587939000	12.517100000	-7.042323000
C	4.058971000	14.923800000	-5.754785000
C	5.340590000	15.113680000	-3.317547000
C	4.639913000	9.532198000	-8.900329000
C	2.999106000	11.218444000	-9.459964000
C	7.287281000	13.661835000	-7.960378000
C	3.655722000	15.881111000	-4.818621000
C	4.292300000	15.966858000	-3.590460000
C	4.158748000	10.513923000	-9.751091000
Bi	2.257818000	12.745008000	-10.947225000
C	7.933327000	13.130862000	-9.093601000
C	7.718583000	14.894945000	-7.475258000
C	0.828058000	13.687777000	-9.457963000
C	0.765708000	11.298755000	-11.831356000
C	8.983266000	13.814849000	-9.691251000
C	8.763655000	15.578757000	-8.093869000
C	1.285928000	14.800819000	-8.742490000
C	-0.426420000	13.186051000	-9.206479000
C	0.538070000	10.043833000	-11.309224000
C	0.125711000	11.648596000	-13.021422000
C	9.403507000	15.034563000	-9.186074000
C	0.483920000	15.360916000	-7.761409000
C	-0.319789000	9.146983000	-11.942763000
C	-0.761347000	10.759380000	-13.640388000
C	-0.780254000	14.853604000	-7.500197000
C	-0.964093000	9.514324000	-13.111557000
C	-1.231289000	13.766728000	-8.221287000
H	7.231438000	15.336250000	1.041329000
H	3.516039000	14.857400000	0.938553000
H	2.819004000	16.546018000	2.591790000
H	3.969931000	16.704694000	4.788863000
H	5.849034000	15.170793000	5.290169000
H	6.572251000	13.497956000	3.639204000
H	5.389285000	9.916214000	2.442411000
H	6.792587000	8.602508000	3.979337000
H	9.155976000	9.229585000	4.365761000
H	10.109893000	11.208272000	3.196758000
H	8.702220000	12.542327000	1.663308000
H	9.080822000	16.544334000	-0.053374000
H	7.617246000	12.169345000	-1.870416000
H	10.225545000	15.567989000	-2.021325000
H	9.472481000	13.382642000	-2.924001000
H	2.266111000	9.744488000	1.156088000
H	-1.406299000	15.303608000	0.915480000
H	2.274760000	15.219372000	-1.298491000
H	0.847262000	16.220948000	0.447795000
H	-2.226601000	13.354113000	-0.385815000
H	-0.801789000	12.321800000	-2.120720000
H	1.034373000	9.732753000	-2.744475000
H	-0.480288000	8.154245000	-3.871732000
H	-1.640247000	8.810097000	-5.962611000

H	-1.260706000	11.060548000	-6.922261000
H	0.287746000	12.627963000	-5.837056000
H	4.718633000	10.731824000	-3.022145000
H	5.557611000	8.996403000	-1.504428000
H	4.330188000	8.480797000	0.586888000
H	2.260288000	9.748548000	-6.489654000
H	1.430112000	11.487816000	-0.367620000
H	6.576281000	13.505000000	-3.998006000
H	5.394117000	9.910173000	-5.208533000
H	6.796519000	8.597961000	-3.670563000
H	9.157885000	9.230674000	-3.276991000
H	10.112804000	11.205675000	-4.447163000
H	8.703155000	12.543086000	-5.980052000
H	3.516860000	14.857322000	-6.703158000
H	2.819568000	16.545696000	-5.049551000
H	3.973328000	16.707044000	-2.854128000
H	5.859475000	15.170008000	-2.356643000
H	4.329460000	8.479014000	-7.057332000
H	1.429766000	11.481756000	-8.006535000
H	5.553369000	8.989342000	-9.151727000
H	4.710971000	10.723193000	-10.673308000
H	7.615620000	12.169327000	-9.508101000
H	7.234484000	15.337416000	-6.600737000
H	9.481639000	13.384741000	-10.563313000
H	9.082877000	16.544675000	-7.695171000
H	2.275633000	15.223439000	-8.937667000
H	-0.799997000	12.323210000	-9.764250000
H	1.036825000	9.732833000	-10.387741000
H	0.289979000	12.630080000	-13.475709000
H	10.228727000	15.567575000	-9.662547000
H	0.848512000	16.222100000	-7.195369000
H	-0.477542000	8.155609000	-11.513219000
H	-1.265062000	11.060248000	-14.561732000
H	-1.404082000	15.302945000	-6.725919000
H	-1.639352000	8.811465000	-13.604505000
H	-2.225135000	13.356100000	-8.027719000

Tetramer **1b-2**

Bi	5.587324000	12.517070000	0.599591000
C	7.717715000	14.894857000	0.165094000
C	5.110462000	14.081769000	2.163486000
C	4.058808000	14.924095000	1.887058000
C	3.655913000	15.881665000	2.823109000
C	4.292992000	15.967787000	4.050985000
C	5.341420000	15.114722000	4.323716000
C	5.748315000	14.173552000	3.398181000
C	6.948265000	11.330503000	1.949594000
C	6.431910000	10.215514000	2.598892000
C	7.223332000	9.471880000	3.471090000
C	8.537175000	9.818139000	3.685063000
C	9.070296000	10.924569000	3.033554000
C	8.270899000	11.676589000	2.167335000
C	7.286253000	13.661592000	-0.319492000
C	8.762512000	15.578522000	-0.454144000
C	7.931852000	13.130312000	-1.452826000
C	9.401933000	15.034032000	-1.546454000
C	8.981524000	13.814158000	-2.051105000
Bi	2.257015000	12.745129000	4.338489000
C	0.827836000	13.688284000	5.828063000
C	1.285964000	14.801548000	6.543026000
C	0.484340000	15.361904000	7.524273000
C	0.764589000	11.298572000	3.455389000
C	0.537204000	10.043794000	3.977979000
C	0.124093000	11.648048000	2.265484000
C	2.998962000	11.219019000	5.825888000
C	4.158506000	10.514449000	5.534491000
C	4.640051000	9.532987000	6.385339000
C	3.960376000	9.253549000	7.552316000
C	2.801548000	9.957906000	0.229113000
C	2.338440000	10.937996000	7.011432000
C	-0.781130000	14.853240000	0.143650000
C	-1.231007000	13.767529000	7.065561000
C	-0.426523000	13.186591000	6.080207000
C	-0.320886000	9.146734000	3.345051000
C	-0.965680000	9.513715000	2.176414000

C	-0.763191000	10.758624000	1.647140000
Bi	2.255596000	12.743742000	-3.304011000
C	0.826417000	13.686896000	-1.814437000
C	1.284545000	14.800160000	-1.099474000
C	0.482921000	15.360516000	-0.118227000
C	-0.779710000	14.854627000	7.786150000
C	-1.232426000	13.766142000	-0.576939000
C	-0.427943000	13.185203000	-1.562293000
C	0.763169000	11.297185000	-4.187111000
C	0.535785000	10.042407000	-3.664521000
C	-0.322306000	9.145346000	-4.297449000
C	-0.967100000	9.512328000	-5.466086000
C	-0.764611000	10.757236000	-5.995359000
C	0.122673000	11.646660000	-5.377016000
C	2.997542000	11.217632000	-1.816612000
C	4.157086000	10.513062000	-2.108009000
C	4.638631000	9.531599000	-1.257160000
C	3.958956000	9.252162000	-0.090183000
C	2.802968000	9.959293000	7.871613000
C	2.337020000	10.936608000	-0.631068000
C	5.109042000	14.080381000	-5.479014000
C	5.746895000	14.172165000	-4.244319000
C	6.946846000	11.329116000	-5.692906000
C	6.430490000	10.214127000	-5.043607000
C	7.221912000	9.470492000	-4.171409000
C	8.535755000	9.816751000	-3.957436000
C	9.068876000	10.923182000	-4.608946000
C	8.269479000	11.675202000	-5.475164000
Bi	5.585904000	12.515682000	-7.042908000
C	4.057388000	14.922707000	-5.755441000
C	5.340001000	15.113335000	-3.318784000
C	7.284834000	13.660204000	-7.961992000
C	3.654493000	15.880277000	-4.819390000
C	4.291572000	15.966400000	-3.591514000
C	7.930433000	13.128924000	-9.095325000
C	7.716295000	14.893469000	-7.477406000
C	8.980104000	13.812771000	-9.693604000
C	8.761093000	15.577134000	-8.096644000
C	9.400513000	15.032644000	-9.188954000
H	7.233401000	15.337897000	1.039185000
H	3.514978000	14.855298000	0.939741000
H	2.818225000	16.544819000	2.593899000
H	3.971241000	16.706559000	4.787417000
H	5.849583000	15.170471000	5.290002000
H	6.572886000	13.498247000	3.639714000
H	5.394182000	9.911094000	2.434821000
H	6.795937000	8.600464000	3.973289000
H	9.158949000	9.231081000	4.363962000
H	10.112186000	11.209557000	3.194309000
H	8.703965000	12.543219000	1.661138000
H	9.081505000	16.544974000	-0.056605000
H	7.616843000	12.169701000	-1.871739000
H	10.225608000	15.568169000	-2.024378000
H	9.472316000	13.382475000	-2.926249000
H	2.278688000	15.217330000	6.349723000
H	0.849254000	16.222583000	8.091037000
H	1.036950000	9.734363000	4.899675000
H	0.287765000	12.628016000	1.806784000
H	4.718443000	10.731799000	4.619751000
H	5.558123000	8.997022000	6.135353000
H	4.330134000	8.482253000	8.231674000
H	2.261230000	9.748559000	1.155289000
H	1.430994000	11.488299000	7.275142000
H	2.268577000	9.745723000	8.799713000
H	-1.404354000	15.302094000	0.918811000
H	-2.224494000	13.355581000	7.259050000
H	-0.800092000	12.322869000	5.523799000
H	-0.479294000	8.154879000	3.773945000
H	-1.640084000	8.810072000	1.682965000
H	-1.260707000	11.059960000	0.722504000
H	2.275394000	15.218924000	-1.297967000
H	0.847359000	16.221460000	0.448815000
H	-1.404539000	15.306298000	8.558651000
H	-2.225863000	13.355252000	-0.381249000
H	-0.800886000	12.321319000	-2.119033000
H	1.033407000	9.733054000	-2.741787000
H	-0.481251000	8.153998000	-3.867813000
H	-1.643513000	8.809759000	-5.958138000

H	-1.269380000	11.056914000	-6.916720000
H	0.287225000	12.628940000	-5.830325000
H	4.717490000	10.730142000	-3.022554000
H	5.556601000	8.994945000	-1.505832000
H	4.328652000	8.479001000	0.586778000
H	1.428238000	11.480649000	-0.362867000
H	6.574185000	13.503201000	-3.998500000
H	5.393982000	9.904060000	-5.208253000
H	6.795268000	8.597392000	-3.672073000
H	9.157591000	9.230052000	-3.278551000
H	10.111842000	11.205447000	-4.450286000
H	8.702074000	12.541368000	-5.982570000
H	3.523237000	14.865613000	-6.708931000
H	2.821298000	16.546945000	-5.053038000
H	3.973587000	16.708048000	-2.856297000
H	5.860588000	15.169377000	-2.358901000
H	7.614308000	12.167906000	-9.511166000
H	7.231498000	15.337588000	-6.604261000
H	9.476787000	13.381940000	-10.565965000
H	9.079245000	16.543176000	-7.698033000
H	10.224691000	15.566200000	-9.666462000

Tetramer **1b-3**

C	19.393794000	10.762562000	7.327299000
C	20.039879000	11.293367000	6.194020000
C	21.089341000	10.608927000	5.596052000
C	21.509057000	9.388912000	6.100962000
C	20.869152000	8.844899000	7.193220000
Bi	17.695223000	11.908039000	8.245875000
C	19.824560000	9.529148000	7.812149000
C	17.217297000	10.343795000	9.809897000
C	19.056476000	13.094140000	9.595983000
C	16.165291000	9.501939000	9.533377000
C	17.854856000	10.251880000	11.044734000
C	15.761743000	8.544698000	10.469484000
C	17.447318000	9.311039000	11.970321000
C	16.398532000	8.458443000	11.697501000
C	18.540529000	14.209472000	10.245017000
C	20.378898000	12.747445000	9.814042000
C	19.332134000	14.952848000	11.117269000
C	21.178473000	13.499191000	10.680314000
C	20.645776000	14.605983000	11.331557000
Bi	14.364058000	11.682128000	11.984131000
Bi	21.958508000	3.540578000	8.162696000
C	12.934121000	10.739879000	13.473551000
C	23.388372000	2.598293000	9.652163000
Bi	18.627336000	3.766281000	12.066829000
C	13.391565000	9.626496000	14.188766000
C	22.930876000	1.484921000	10.367363000
C	12.589462000	9.066669000	15.169931000
C	23.732922000	0.925074000	11.348555000
C	10.874990000	10.661808000	14.710644000
C	11.679954000	11.242215000	13.725370000
C	12.872511000	13.129281000	11.100522000
C	12.645628000	14.384244000	11.622886000
C	11.788100000	15.281629000	10.989656000
C	11.143355000	14.914791000	9.820941000
C	11.345358000	13.669709000	9.291887000
C	12.232086000	12.779944000	9.910538000
C	15.106443000	13.208092000	13.471462000
C	21.216113000	5.066560000	9.650002000
C	16.266386000	13.912058000	13.180198000
C	20.056197000	5.770556000	9.358699000
C	16.748235000	14.893409000	14.031003000
C	19.574345000	6.751920000	10.209489000
C	16.068459000	15.173343000	15.197803000
C	20.254089000	7.031837000	11.376311000
C	21.411875000	6.326670000	11.695513000
C	14.445817000	13.489605000	14.656831000
C	21.876707000	5.348057000	10.835393000
C	16.928777000	2.620847000	11.148197000
C	16.282743000	3.151669000	10.014897000
C	15.233284000	2.467255000	9.416893000
C	14.813510000	1.247261000	9.921789000
C	15.453375000	0.703221000	11.014069000

C	16.497963000	1.387454000	11.633033000
C	19.105181000	2.202025000	13.630867000
C	20.157175000	1.360142000	13.354382000
C	20.560667000	0.402891000	14.290503000
C	18.467579000	2.110126000	14.865683000
C	17.266079000	4.952416000	13.416892000
C	17.782032000	6.067735000	14.065943000
C	16.990417000	6.811132000	14.938168000
C	15.676759000	6.464300000	15.152413000
C	15.144056000	5.357522000	14.501152000
C	15.943641000	4.605755000	13.634907000
C	23.450111000	4.987693000	7.279136000
C	24.642544000	3.100597000	9.904023000
C	11.325614000	9.574595000	15.431480000
C	24.996784000	1.432968000	11.610146000
C	14.910645000	14.468196000	15.516967000
C	19.923835000	0.316652000	15.518499000
C	18.875062000	1.169275000	15.791284000
C	23.677019000	6.242650000	7.801508000
C	24.090577000	4.638340000	6.089173000
C	24.534590000	7.140013000	7.168306000
C	25.179355000	6.773160000	5.999613000
C	25.447450000	2.520169000	10.889325000
C	24.977348000	5.528083000	5.470552000
H	19.724447000	12.254704000	5.778201000
H	21.586429000	11.039893000	4.723868000
H	22.334197000	8.850449000	5.630029000
H	21.190031000	7.882385000	7.597546000
H	19.345580000	9.088358000	8.688183000
H	15.631433000	9.561339000	8.579817000
H	18.680538000	10.926696000	11.284329000
H	14.928588000	7.876727000	10.239718000
H	17.951579000	9.251975000	12.938444000
H	16.082489000	7.717553000	12.432744000
H	17.503967000	14.519500000	10.081041000
H	20.811385000	11.880793000	9.307409000
H	18.904736000	15.825242000	11.617142000
H	22.220231000	13.213655000	10.839855000
H	21.267847000	15.193628000	12.009519000
H	14.381645000	9.204705000	13.994274000
H	21.940848000	1.062592000	10.173097000
H	12.950501000	8.200972000	15.731649000
H	23.367747000	0.063837000	11.914119000
H	9.881316000	11.073567000	14.903101000
H	11.306290000	12.105350000	13.168148000
H	13.145003000	14.693498000	12.544709000
H	11.629249000	16.273177000	11.418934000
H	10.467042000	15.617451000	9.328813000
H	10.840602000	13.370453000	8.370318000
H	12.395782000	11.797185000	9.457938000
H	16.826208000	13.694063000	12.265690000
H	19.504192000	5.562433000	8.436071000
H	17.666103000	15.429691000	13.781093000
H	18.653353000	7.287497000	9.970488000
H	16.438216000	15.944893000	15.876800000
H	19.879876000	7.806360000	12.047453000
H	21.947917000	6.539858000	12.622615000
H	13.537965000	12.939873000	14.920218000
H	22.782398000	4.795713000	11.100102000
H	16.599058000	4.114392000	9.601642000
H	14.734874000	2.897154000	8.544748000
H	13.988768000	0.713771000	9.445045000
H	15.134070000	-0.262723000	11.412512000
H	16.983310000	0.945204000	12.506921000
H	20.698226000	1.425816000	12.405307000
H	21.395143000	-0.263240000	14.057893000
H	17.640518000	2.782816000	15.105959000
H	18.820973000	6.369186000	13.905979000
H	17.416160000	7.680014000	15.446430000
H	15.057422000	7.051709000	15.833050000
H	14.101205000	5.074952000	14.659910000
H	15.510968000	3.739319000	13.128098000
H	14.375921000	14.682313000	16.444692000
H	20.244430000	-0.422328000	16.254994000
H	18.364833000	1.110976000	16.756358000
H	25.016760000	3.963339000	9.346715000
H	10.699554000	9.122602000	16.202787000
H	25.621640000	0.981586000	12.382459000

H	23.182831000	6.551063000	8.726342000
H	23.926586000	3.656973000	5.634674000
H	24.688037000	8.132732000	7.596244000
H	25.854842000	7.476394000	5.507377000
H	26.440850000	2.932336000	11.081124000
H	25.481045000	5.227999000	4.549004000

Tetramer **1b-4**

C	19.393627000	10.762313000	7.327207000
C	20.039738000	11.293114000	6.193941000
C	21.089157000	10.608631000	5.595947000
C	21.508805000	9.388577000	6.100819000
C	20.868874000	8.844568000	7.193065000
Bi	17.695127000	11.907860000	8.245824000
C	19.824324000	9.528860000	7.812019000
C	17.217118000	10.343598000	9.809803000
C	19.056454000	13.093843000	9.595961000
C	16.165062000	9.501811000	9.533263000
C	17.854677000	10.251610000	11.044634000
C	15.761463000	8.544566000	10.469344000
C	17.447090000	9.310766000	11.970196000
C	16.398253000	8.458238000	11.697355000
C	18.540575000	14.209186000	10.245030000
C	20.378857000	12.747065000	9.814003000
C	19.332227000	14.952491000	11.117300000
C	21.178480000	13.498740000	10.680294000
C	20.645850000	14.605544000	11.331572000
Bi	14.363966000	11.682032000	11.984090000
Bi	2.256491000	12.742524000	11.984017000
C	12.933982000	10.739822000	13.473489000
C	0.826640000	13.684734000	13.473544000
C	13.391365000	9.626391000	14.188669000
C	1.284179000	14.798003000	14.188875000
C	12.589234000	9.066581000	15.169821000
C	0.482137000	15.357793000	15.170103000
C	10.874852000	10.661833000	14.710589000
C	-1.232480000	13.762845000	14.710654000
C	11.679845000	11.242223000	13.725328000
C	-0.427569000	13.182475000	13.725315000
C	12.872499000	13.129297000	11.100530000
C	0.764820000	11.295589000	11.100261000
C	12.645691000	14.384257000	11.622932000
C	0.537835000	10.040590000	11.622494000
C	11.788212000	15.281711000	10.989733000
C	-0.319771000	9.143343000	10.989173000
C	11.143440000	14.914945000	9.821010000
C	-0.964481000	9.510357000	9.820500000
C	11.345368000	13.669867000	9.291918000
C	-0.762387000	10.755479000	9.291576000
C	12.232048000	12.780032000	9.910538000
C	0.124418000	11.645105000	9.910317000
C	15.106446000	13.207909000	13.471462000
C	2.998756000	11.216341000	13.471183000
C	16.266429000	13.911817000	13.180213000
C	4.158640000	10.512309000	13.179839000
C	16.748338000	14.893115000	14.031045000
C	4.640410000	9.530828000	14.030538000
C	16.068584000	15.173054000	15.197856000
C	3.960617000	9.250827000	15.197311000
C	14.445842000	13.489425000	14.656843000
C	2.338112000	10.934758000	14.656526000
C	7.286190000	13.661648000	14.969759000
C	7.932226000	13.130909000	13.836421000
C	8.981742000	13.815326000	13.238520000
C	9.401561000	15.035242000	13.743557000
C	8.761707000	15.579203000	14.835877000
C	11.325417000	9.574572000	15.431391000
C	-0.781762000	14.849945000	15.431603000
C	14.910730000	14.467964000	15.517005000
C	2.802863000	9.956027000	15.516555000
Bi	5.587539000	12.516215000	15.888221000
C	7.717061000	14.894965000	15.454738000
C	5.109740000	14.080333000	17.452412000
C	6.948689000	11.329858000	17.238197000
C	4.057803000	14.922306000	17.175986000

C	5.747312000	14.172065000	18.687255000
C	6.432653000	10.214501000	17.887116000
C	8.271141000	11.676420000	17.456287000
C	3.654339000	15.879481000	18.112196000
C	5.339857000	15.112841000	19.612944000
C	7.224200000	9.470966000	18.759285000
C	9.070667000	10.924505000	18.322475000
C	4.291140000	15.965553000	19.340219000
C	8.537862000	9.817699000	18.973604000
H	19.724233000	12.254315000	5.778085000
H	21.586336000	11.039156000	4.723725000
H	22.333203000	8.854819000	5.623956000
H	21.186417000	7.878223000	7.591422000
H	19.338592000	9.084880000	8.684658000
H	15.630396000	9.560356000	8.580252000
H	18.678469000	10.927495000	11.286828000
H	14.926212000	7.879991000	10.237697000
H	17.956150000	9.255612000	12.935763000
H	16.075453000	7.720078000	12.433692000
H	17.504032000	14.519339000	10.081222000
H	20.812061000	11.881124000	9.306881000
H	18.904750000	15.824990000	11.616879000
H	22.220165000	13.213063000	10.839856000
H	21.267983000	15.192923000	12.009677000
H	14.384796000	9.211123000	13.997503000
H	2.274366000	15.220049000	13.994820000
H	12.954446000	8.205798000	15.736412000
H	0.847705000	16.218556000	15.736153000
H	9.886800000	11.082658000	14.912799000
H	-2.225839000	13.350545000	14.902487000
H	11.306658000	12.110329000	13.177395000
H	-0.801604000	12.319913000	13.167559000
H	13.144558000	14.692302000	12.545502000
H	1.037460000	9.729564000	12.543385000
H	11.636082000	16.277616000	11.411625000
H	-0.477149000	8.151858000	11.418600000
H	10.466915000	15.617682000	9.329559000
H	-1.639338000	8.807051000	9.327555000
H	10.839761000	13.370807000	8.370769000
H	-1.266479000	11.056047000	8.370372000
H	12.397041000	11.797809000	9.457273000
H	0.288521000	12.626559000	9.455889000
H	16.826851000	13.694023000	12.266016000
H	4.710644000	10.722055000	12.257757000
H	17.666547000	15.428949000	13.781357000
H	5.555064000	8.989607000	13.780321000
H	16.438465000	15.944460000	15.876975000
H	4.333146000	8.479329000	15.874179000
H	13.537335000	12.940412000	14.919737000
H	1.432250000	11.486795000	14.921261000
H	7.615209000	12.169685000	13.420054000
H	9.479239000	13.396961000	12.359931000
H	10.229785000	15.560112000	13.266787000
H	9.081804000	16.544360000	15.235935000
H	14.375064000	14.682483000	16.444064000
H	2.267990000	9.743480000	16.444406000
H	10.697522000	9.131058000	16.206428000
H	-1.406564000	15.301366000	16.203960000
H	7.231401000	15.336813000	16.328676000
H	3.517580000	14.857889000	16.226345000
H	6.574344000	13.499073000	18.926933000
H	5.391799000	9.916704000	17.731711000
H	8.703484000	12.544782000	16.952187000
H	2.820222000	16.546102000	17.879495000
H	5.850600000	15.171474000	20.577764000
H	6.796239000	8.603404000	19.267662000
H	10.113540000	11.208188000	18.478975000
H	3.970520000	16.704434000	20.076852000
H	9.159899000	9.231601000	19.653234000

Tetramer **1c-1**

C	1.160464000	8.092224000	0.619391000
C	0.708456000	7.090187000	1.475911000
C	0.626951000	7.300347000	2.851005000
C	1.012390000	8.522671000	3.383708000

C	1.479772000	9.528085000	2.541332000
C	1.541626000	9.313429000	1.165032000
Bi	4.434108000	6.229945000	2.036740000
C	4.199371000	5.653674000	-0.124163000
C	6.049361000	4.708585000	2.414976000
C	6.506968000	3.836479000	1.439316000
C	7.465707000	2.877534000	1.739894000
C	7.986953000	2.787326000	3.026313000
C	7.546397000	3.662807000	4.001118000
C	6.572251000	4.610513000	3.710021000
C	5.866101000	7.927771000	1.651684000
C	7.220664000	7.850112000	1.951777000
C	8.028431000	8.981932000	1.856082000
C	7.489760000	10.194787000	1.471686000
C	6.144009000	10.280296000	1.148651000
C	5.341926000	9.156340000	1.238652000
Bi	1.187069000	7.706276000	-1.603276000
C	2.187467000	9.665184000	-2.064448000
C	-0.916460000	8.480586000	-1.866131000
C	3.572152000	9.742299000	-1.949178000
C	1.477988000	10.803660000	-2.447667000
C	-1.663943000	7.989013000	-2.931964000
C	-1.521396000	9.381092000	-0.988171000
C	4.237202000	10.942613000	-2.190210000
C	2.147768000	12.000600000	-2.691444000
C	-2.973484000	8.398374000	-3.136227000
C	-2.839102000	9.787091000	-1.183806000
C	3.523149000	12.071207000	-2.549094000
C	-3.561128000	9.299663000	-2.261370000
C	3.184973000	4.759922000	-0.450264000
C	5.050628000	6.111398000	-1.125138000
C	3.022037000	4.329519000	-1.759584000
C	4.892411000	5.669752000	-2.437207000
C	3.872690000	4.782750000	-2.752445000
H	0.410380000	6.115826000	1.077628000
H	0.257721000	6.503483000	3.500443000
H	0.958958000	8.693178000	4.460607000
H	1.790772000	10.489264000	2.955842000
H	1.895704000	10.116856000	0.513892000
H	2.507811000	4.380824000	0.319119000
H	2.215678000	3.633255000	-2.000025000
H	3.743541000	4.441854000	-3.781686000
H	5.567300000	6.030915000	-3.216477000
H	5.856431000	6.809299000	-0.882236000
H	1.223612000	3.975056000	3.627205000
H	3.546763000	3.236978000	3.978141000
H	4.598866000	1.640064000	2.411265000
H	3.301789000	0.756815000	0.483074000
H	0.962185000	1.475261000	0.131817000
C	1.663954000	3.253026000	2.931977000
C	2.973495000	2.843666000	3.136240000
C	3.561139000	1.942376000	2.261383000
C	2.839112000	1.454949000	1.183819000
C	1.521406000	1.860947000	0.988194000
C	0.916471000	2.761453000	1.866144000
Bi	-1.187059000	3.535764000	1.603289000
C	-1.160454000	3.149815000	-0.619378000
C	-2.187456000	1.576855000	2.064471000
C	-0.708446000	4.151852000	-1.475898000
C	-1.541616000	1.928610000	-1.165019000
C	-3.572141000	1.499740000	1.949191000
C	-1.477977000	0.438380000	2.447680000
H	-0.410359000	5.126208000	-1.077610000
C	-0.626941000	3.941692000	-2.850992000
C	-1.479761000	1.713954000	-2.541319000
H	-1.895597000	1.125166000	-0.513846000
H	-4.155105000	2.380142000	1.663666000
C	-4.237191000	0.299427000	2.190214000
C	-2.147758000	-0.758560000	2.691457000
H	-0.390682000	0.475783000	2.547510000
H	-0.257708000	4.738554000	-3.500430000
C	-1.012379000	2.719368000	-3.383695000
H	-1.791028000	0.752875000	-2.955861000
H	-5.323185000	0.255426000	2.081792000
C	-3.523139000	-0.829168000	2.549107000
H	-1.580136000	-1.644253000	2.985710000
H	-0.958934000	2.548854000	-4.460592000
H	-4.044304000	-1.770989000	2.732993000

H	6.108187000	3.893011000	0.423628000
H	7.811278000	2.193845000	0.960982000
H	7.661885000	6.901689000	2.268825000
H	9.091025000	8.907593000	2.099174000
H	8.124507000	11.081450000	1.412138000
H	5.711645000	11.231161000	0.828477000
H	4.278172000	9.245522000	1.000906000
H	8.743344000	2.036350000	3.261748000
H	7.954537000	3.601884000	5.013049000
H	6.228119000	5.283553000	4.501144000
H	4.155140000	8.861874000	-1.663774000
H	5.323198000	10.986612000	-2.081815000
H	4.044313000	13.013028000	-2.732984000
H	1.580149000	12.886288000	-2.985715000
H	0.390711000	10.766204000	-2.547686000
H	-1.223597000	7.266984000	-3.627190000
H	-3.546751000	8.005061000	-3.978128000
H	-4.598855000	9.601977000	-2.411250000
H	-3.301783000	10.485224000	-0.483062000
H	-0.962176000	9.766773000	-0.131791000
H	-2.507867000	6.861295000	-0.319125000
C	-3.184963000	6.482117000	0.450277000
C	-4.199360000	5.588366000	0.124166000
C	-3.022026000	6.912520000	1.759597000
H	-2.215695000	7.608820000	2.000026000
H	-3.743547000	6.800197000	3.781697000
H	-5.567651000	5.211570000	3.216374000
H	-5.856637000	4.433006000	0.882202000
Bi	-4.434098000	5.012095000	-2.036726000
C	-5.050618000	5.130641000	1.125151000
C	-3.872679000	6.459289000	2.752459000
C	-4.892400000	5.572288000	2.437210000
C	-6.049351000	6.533455000	-2.414952000
C	-5.866090000	3.314269000	-1.651671000
C	-6.506958000	7.405560000	-1.439303000
C	-6.572241000	6.631526000	-3.710008000
C	-7.220654000	3.391927000	-1.951764000
C	-5.341916000	2.085699000	-1.238639000
H	-6.108174000	7.349034000	-0.423614000
C	-7.465696000	8.364506000	-1.739881000
C	-7.546387000	7.579233000	-4.001104000
H	-6.228107000	5.958485000	-4.501129000
H	-7.661873000	4.340349000	-2.268818000
C	-8.028421000	2.260107000	-1.856069000
C	-6.143999000	0.961753000	-1.148648000
H	-4.278127000	1.996446000	-1.001076000
H	-7.811267000	9.048196000	-0.960969000
C	-7.986943000	8.454713000	-3.026300000
H	-7.954526000	7.640156000	-5.013037000
H	-9.091017000	2.334451000	-2.099150000
C	-7.489750000	1.047262000	-1.471673000
H	-5.711597000	0.010858000	-0.828615000
H	-8.743334000	9.205689000	-3.261735000
H	-8.124485000	0.160588000	-1.412160000

Tetramer **1c-2**

C	1.159220000	8.090230000	0.617908000
C	0.707875000	7.087562000	1.474039000
C	0.626455000	7.297054000	2.849239000
C	1.011313000	8.519343000	3.382441000
C	1.478033000	9.525390000	2.540454000
C	1.539804000	9.311392000	1.164048000
Bi	4.434070000	6.229068000	2.033940000
C	4.199330000	5.653651000	-0.127191000
C	6.050195000	4.708406000	2.411253000
C	6.508130000	3.836989000	1.435132000
C	7.467428000	2.878423000	1.735136000
C	7.988908000	2.787912000	3.021439000
C	7.548022000	3.662713000	3.996704000
C	6.573324000	4.610028000	3.706178000
C	5.865093000	7.927839000	1.649448000
C	7.219742000	7.850773000	1.949311000
C	8.026887000	8.983070000	1.854014000
C	7.487508000	10.195809000	1.470245000
C	6.141664000	10.280741000	1.147443000

C	5.340199000	9.156313000	1.237050000
Bi	1.185712000	7.705304000	-1.604938000
C	2.184990000	9.664960000	-2.065365000
C	-0.918271000	8.478603000	-1.867139000
C	3.569649000	9.742767000	-1.950260000
C	1.474843000	10.803227000	-2.447965000
C	-1.665644000	7.987111000	-2.933087000
C	-1.523564000	9.378384000	-0.988683000
C	4.234019000	10.943547000	-2.190843000
C	2.143945000	12.000638000	-2.691296000
C	-2.975434000	8.395860000	-3.136976000
C	-2.841516000	9.783763000	-1.183944000
C	3.519308000	12.071920000	-2.549111000
C	-3.563436000	9.296436000	-2.261625000
C	3.185366000	4.759503000	-0.453551000
C	5.050197000	6.112288000	-1.128080000
C	3.022472000	4.329606000	-1.763042000
C	4.892028000	5.671152000	-2.440327000
C	3.872738000	4.783745000	-2.755821000
H	0.402341000	6.117052000	1.070676000
H	0.265542000	6.499573000	3.501866000
H	0.957001000	8.689641000	4.459325000
H	1.787573000	10.487027000	2.955276000
H	1.893436000	10.115696000	0.513628000
H	6.120864000	3.895297000	0.415831000
H	7.815757000	2.203008000	0.951753000
H	7.679472000	4.339771000	-2.257305000
H	6.254522000	2.339590000	-2.088881000
H	7.222787000	0.160467000	-1.406516000
H	9.636695000	0.011253000	-0.825384000
H	11.069393000	1.996729000	-0.999532000
C	8.126058000	3.391277000	-1.949311000
C	7.318914000	2.258980000	-1.854013000
C	7.858292000	1.046250000	-1.470245000
C	9.204136000	0.961319000	-1.147453000
C	10.005611000	2.085736000	-1.237049000
C	9.480707000	3.314211000	-1.649448000
Bi	10.911730000	5.012982000	-2.033939000
C	11.146470000	5.588398000	0.127181000
C	9.295606000	6.533644000	-2.411242000
C	12.160434000	6.482547000	0.453551000
C	10.295603000	5.129762000	1.128080000
C	8.837670000	7.405060000	-1.435132000
C	8.772476000	6.632022000	-3.706178000
H	12.841648000	6.854050000	-0.318127000
C	12.323328000	6.912444000	1.763043000
C	10.453773000	5.570898000	2.440317000
H	9.486786000	4.435072000	0.893140000
H	9.224935000	7.346757000	-0.415830000
C	7.878373000	8.363627000	-1.735136000
C	7.797779000	7.579337000	-3.996704000
H	9.116526000	5.960062000	-4.498127000
H	13.127730000	7.609501000	2.009576000
C	11.473062000	6.458305000	2.755821000
H	9.774476000	5.205466000	3.213074000
H	7.530044000	9.039042000	-0.951753000
C	7.356893000	8.454138000	-3.021439000
H	7.390880000	7.641603000	-5.009362000
H	11.602210000	6.798773000	3.785135000
H	6.594497000	9.199516000	-3.256182000
H	7.666329000	6.902279000	2.257305000
H	9.091277000	8.902461000	2.088883000
H	8.123018000	11.081590000	1.406520000
H	5.709100000	11.230803000	0.825370000
H	4.276416000	9.245319000	0.999530000
H	8.751303000	2.042534000	3.256182000
H	7.954921000	3.600447000	5.009362000
H	6.229277000	5.281987000	4.498130000
H	4.153981000	8.861817000	-1.668841000
H	0.387476000	10.764952000	-2.547064000
H	-1.221965000	7.270928000	-3.632345000
H	-3.543473000	8.003852000	-3.983552000
H	-4.596216000	9.614776000	-2.417690000
H	-3.301676000	10.487219000	-0.486560000
H	-0.964838000	9.765102000	-0.132558000
H	5.319783000	10.991734000	-2.078714000
H	1.576213000	12.886486000	-2.984946000
H	4.040023000	13.014156000	-2.732223000

H	2.504154000	4.388002000	0.318129000
H	2.218071000	3.632548000	-2.009577000
H	3.743586000	4.443274000	-3.785133000
H	5.571324000	6.036584000	-3.213084000
H	5.859014000	6.806978000	-0.893139000
Bi	14.160088000	3.536746000	1.604938000
C	14.186590000	3.151820000	-0.617907000
C	13.160811000	1.577090000	2.065375000
C	16.264082000	2.763447000	1.867139000
C	14.637925000	4.154488000	-1.474038000
C	13.805996000	1.930658000	-1.164048000
C	11.776151000	1.499283000	1.950260000
C	13.870957000	0.438823000	2.447965000
C	17.011444000	3.254938000	2.933087000
C	16.869365000	1.863666000	0.988693000
H	14.943459000	5.124999000	-1.070677000
C	14.719345000	3.944996000	-2.849239000
C	13.867767000	1.716660000	-2.540454000
H	13.452364000	1.126355000	-0.513627000
H	11.191821000	2.380234000	1.668840000
C	11.111781000	0.298503000	2.190833000
C	13.201865000	-0.758588000	2.691296000
H	14.958324000	0.477102000	2.547062000
H	16.567765000	3.971123000	3.632343000
C	18.321234000	2.846190000	3.136976000
C	18.187317000	1.458287000	1.183944000
H	16.310636000	1.476946000	0.132571000
H	15.080256000	4.742478000	-3.501866000
C	14.334487000	2.722707000	-3.382441000
H	13.558224000	0.755024000	-2.955276000
H	10.026018000	0.250313000	2.078697000
C	11.826492000	-0.829870000	2.549112000
H	13.769597000	-1.644436000	2.984946000
H	18.889273000	3.238196000	3.983552000
C	18.909237000	1.945614000	2.261625000
H	18.647474000	0.754829000	0.486560000
H	14.388800000	2.552408000	-4.459324000
H	11.305778000	-1.772106000	2.732222000
H	19.942017000	1.627276000	2.417690000

Tetramer **1c-3**

C	1.160598000	8.090436000	0.619707000
C	0.708764000	7.087830000	1.475652000
C	0.627518000	7.297082000	2.850900000
C	1.013044000	8.519058000	3.384336000
C	1.480256000	9.525032000	2.542534000
C	1.541851000	9.311285000	1.166082000
Bi	4.434531000	6.227258000	2.035205000
C	4.199389000	5.652410000	-0.126033000
C	6.049872000	4.705666000	2.412131000
C	6.507303000	3.834209000	1.435809000
C	7.466109000	2.875076000	1.735572000
C	7.987601000	2.784025000	3.021832000
C	7.547220000	3.658857000	3.997298000
C	6.573009000	4.606745000	3.707012000
C	5.866432000	7.925353000	1.650998000
C	7.221054000	7.847511000	1.950782000
C	8.028791000	8.979403000	1.855681000
C	7.490034000	10.192505000	1.472187000
C	6.144220000	10.278212000	1.149464000
C	5.342167000	9.154189000	1.238876000
Bi	1.186785000	7.705955000	-1.603218000
C	2.187074000	9.665178000	-2.063288000
C	-0.916802000	8.480416000	-1.865164000
C	3.571780000	9.742232000	-1.948230000
C	1.477510000	10.803898000	-2.445621000
C	-1.664483000	7.989538000	-2.931178000
C	-1.521581000	9.380336000	-0.986495000
C	4.236772000	10.942711000	-2.188597000
C	2.147232000	12.001006000	-2.688736000
C	-2.974067000	8.399019000	-3.134923000
C	-2.839328000	9.786449000	-1.181611000
C	3.522639000	12.071535000	-2.546600000
C	-3.561554000	9.299725000	-2.259360000
C	3.184940000	4.758863000	-0.452531000

C	5.050452000	6.110804000	-1.126867000
C	3.021759000	4.329322000	-1.762103000
C	4.891990000	5.670021000	-2.439197000
C	3.872219000	4.783217000	-2.754827000
H	4.135289000	9.226962000	3.619745000
C	4.547937000	9.280604000	4.627179000
H	4.279550000	9.242627000	0.994106000
C	4.107168000	8.406167000	5.602815000
C	5.522568000	10.228126000	4.917271000
C	4.628700000	8.497236000	6.889057000
H	3.349930000	7.656449000	5.363594000
C	6.045750000	10.327066000	6.212143000
H	5.873763000	10.885212000	4.116772000
C	5.587942000	9.456002000	7.188634000
H	4.284401000	7.814040000	7.669101000
Bi	7.661765000	11.848014000	6.588763000
H	5.981258000	9.515155000	8.206429000
C	7.896652000	11.273482000	8.750113000
C	6.230618000	13.546819000	6.972650000
C	8.910706000	10.379539000	9.076794000
C	7.045793000	11.732438000	9.750858000
C	4.875962000	13.469520000	6.672881000
C	6.755439000	14.775493000	7.384533000
H	9.591454000	10.008287000	8.304745000
C	9.073696000	9.950190000	10.386450000
C	7.204060000	11.291840000	11.063273000
H	6.241056000	12.430818000	9.506503000
H	4.435509000	12.520777000	6.355351000
C	4.068727000	14.601779000	6.767752000
C	5.953884000	15.899889000	7.473716000
H	7.819337000	14.864619000	7.621409000
H	9.877502000	9.252921000	10.633807000
C	8.223437000	10.404655000	11.379086000
H	6.530207000	11.653595000	11.842999000
H	3.006163000	14.527469000	6.524682000
C	4.608023000	15.814716000	7.151010000
H	6.387128000	16.850510000	7.793133000
H	8.353413000	10.065698000	12.408559000
H	3.973696000	16.701700000	7.209801000
H	2.503842000	4.388059000	0.319417000
H	2.217812000	3.632165000	-2.009180000
H	3.742171000	4.443961000	-3.784172000
H	5.566040000	6.031279000	-3.218949000
H	5.855798000	6.808511000	-0.882866000
H	6.112871000	3.892877000	0.418441000
H	7.810969000	2.191767000	0.956200000
H	7.660887000	6.899792000	2.271551000
H	9.090741000	8.905064000	2.100845000
H	8.124359000	11.079638000	1.414601000
H	5.711768000	11.228822000	0.828681000
H	8.743450000	2.032242000	3.256397000
H	7.954479000	3.598111000	5.009471000
H	6.230961000	5.281020000	4.498027000
H	0.402492000	6.117696000	1.072085000
H	0.265732000	6.499938000	3.503504000
H	0.962339000	8.691233000	4.460951000
H	1.790202000	10.485436000	2.959685000
H	1.895366000	10.115715000	0.515726000
Bi	10.910168000	13.325977000	10.226909000
H	11.693616000	11.736729000	7.551764000
H	11.829897000	12.118136000	5.120555000
H	11.138557000	14.308082000	4.162265000
H	10.307604000	16.105759000	5.665741000
H	10.201973000	15.735306000	8.107445000
C	10.936535000	13.710005000	8.003899000
C	9.910748000	15.285733000	10.686598000
C	13.014109000	14.099546000	10.488694000
C	11.387915000	12.707032000	7.148149000
C	10.555814000	14.930926000	7.457297000
C	11.469253000	12.915980000	5.772870000
C	10.617504000	15.144379000	6.080803000
C	11.084269000	14.138024000	5.239197000
C	8.526076000	15.363369000	10.571525000
C	10.620817000	16.424203000	11.068700000
H	7.942410000	14.483140000	10.286985000
C	7.861618000	16.564200000	10.811649000
C	9.951637000	17.621655000	11.311592000
H	11.708084000	16.385920000	11.168467000

H	6.775550000	16.608833000	10.703731000
C	8.576251000	17.692776000	11.169443000
H	10.519515000	18.507454000	11.605081000
H	8.055422000	18.634857000	11.352979000
C	13.761561000	13.608543000	11.554814000
C	13.619276000	14.999036000	9.609860000
H	13.318043000	12.892515000	12.254243000
C	15.071327000	14.017482000	11.758469000
C	14.937204000	15.404592000	9.804898000
H	13.060328000	15.385683000	8.753905000
H	15.639363000	13.626037000	12.605264000
C	15.659214000	14.917757000	10.882741000
H	15.397223000	16.107803000	9.107218000
H	16.691883000	15.236401000	11.038714000
H	-1.221354000	7.273150000	-3.630477000
H	-0.962531000	9.767157000	-0.130684000
H	-3.542225000	8.007654000	-3.981654000
H	-3.298954000	10.490027000	-0.484069000
H	-4.594059000	9.618829000	-2.415392000
H	4.155126000	8.861584000	-1.664163000
H	0.390240000	10.766051000	-2.545403000
H	5.322942000	10.986867000	-2.081260000
H	1.579791000	12.887026000	-2.982305000
H	4.043876000	13.013355000	-2.730189000

References:

1 Pfeiffer, P., *Ber. dtsch. chem. Ges.* **1904**, *37*, 4620-4623.
2 Supniewski, J. V.; Adams, R., *J. Am. Chem. Soc.* **1926**, *48*, 507-517.
3 *MestReNova*, Mestrelab Research S. L., A Coruña 15706, Santiago de Compostela.
4 Neese, F., *WIREs: Comput. Mol. Sci.* **2012**, *2*, 73-78.
5 Neese, F., *WIREs: Comput. Mol. Sci.* **2018**, *8*, e1327-n/a.
6 Perdew, J. P.; Burke, K.; Ernzerhof, M., *Phys. Rev. Lett.* **1996**, *77*, 3865-3868.
7 Perdew, J. P.; Burke, K.; Ernzerhof, M., *Phys. Rev. Lett.* **1997**, *78*, 1396-1396.
8 Weigend, F.; Ahlrichs, R., *Phys. Chem. Chem. Phys.* **2005**, *7*, 3297-3305.
9 Grimme, S.; Antony, J.; Ehrlich, S.; Krieg, H., *J. Chem. Phys.* **2010**, *132*, 154104.
10 Becke, A. D.; Johnson, E. R., *J. Chem. Phys.* **2005**, *123*, 154101.
11 Eichkorn, K.; Treutler, O.; Öhm, H.; Häser, M.; Ahlrichs, R., *Chem. Phys. Lett.* **1995**, *240*, 283-290.
12 Neese, F.; Hansen, A.; Liakos, D. G., *J. Chem. Phys.* **2009**, *131*, 064103.
13 Neese, F.; Hansen, A.; Wennmohs, F.; Grimme, S., *Acc. Chem. Res.* **2009**, *42*, 641-648.
14 Hansen, A.; Liakos, D. G.; Neese, F., *J. Chem. Phys.* **2011**, *135*, 214102.
15 Liakos, D. G.; Hansen, A.; Neese, F., *J. Chem. Theory Comput.* **2011**, *7*, 76-87.
16 Riplinger, C.; Neese, F., *J. Chem. Phys.* **2013**, *138*, 034106.
17 Riplinger, C.; Sandhoefer, B.; Hansen, A.; Neese, F., *J. Chem. Phys.* **2013**, *139*, 134101.
18 Riplinger, C.; Pinski, P.; Becker, U.; Valeev, E. F.; Neese, F., *J. Chem. Phys.* **2016**, *144*, 024109.
19 Dunning Jr., T. H., *J. Chem. Phys.* **1989**, *90*, 1007-1023.
20 Peterson, K. A.; Yousaf, K. E., *J. Chem. Phys.* **2010**, *133*, 174116.
21 Liakos, D. G.; Sparta, M.; Kesharwani, M. K.; Martin, J. M. L.; Neese, F., *J. Chem. Theory Comput.* **2015**, *11*, 1525-1539.
22 Liakos, D. G.; Neese, F., *J. Chem. Theory Comput.* **2015**, *11*, 4054-4063.
23 Schneider, W. B.; Bistoni, G.; Sparta, M.; Saitow, M.; Riplinger, C.; Auer, A. A.; Neese, F., *J. Chem. Theory Comput.* **2016**, *12*, 4778-4792.
24 Bistoni, G.; Auer, A. A.; Neese, F., *Chem. Eur. J.* **2017**, *23*, 865-873.
25 Wuttke, A.; Mata, R. A., *J. Comput. Chem.* **2017**, *38*, 15-23.
26 Altun, A.; Neese, F.; Bistoni, G., *J. Chem. Theory Comput.* **2018**, *to be submitted*.
27 Pettersen, E. F.; Goddard, T. D.; Huang, C. C.; Couch, G. S.; Greenblatt, D. M.; Meng, E. C.; Ferrin, T. E., *J. Comput. Chem.* **2004**, *25*, 1605-1612.
28 Chemcraft Version 1.8, h. w. c. c.
29 Wetzel, J., *Z. Kristallogr. Kristallgeom. Kristallphys. Kristallchem.* **1942**, *104*, 305.
30 Hawley, D. M.; Ferguson, G.; Harris, G. S., *Chem.Commun.* **1966**, 111-112.
31 Hawley, D. M.; Ferguson, G., *J. Chem. Soc. A* **1968**, 2059-2063.
32 Jones, P. G.; Blaschette, A.; Henschel, D.; Weitze, A., *Z. Kristallogr.* **1995**, *210*, 377-378.
33 Bučinský, L.; Jayatilaka, D.; Grabowsky, S., *J. Phys. Chem. A* **2016**, *120*, 6650-6669.
34 Andrews, P. C.; MacLellan, J. G., *personal communication*.
35 Stammler, H.-G.; Neumann, B., *Private Communication to the CSD (BITRPH11)* **2014**, DOI: 10.5517/cc13k8qn.
36 Flack, H., *Acta Crystallogr. A* **1983**, *39*, 876-881.
